# Supplementary material for: Tick-Borne Encephalitis Virus Vaccination among Tourists in a High-Prevalence Area (Italy, 2023): A Cross-Sectional Study
Source: Trop Med Infect Dis. 2023 Nov 2;8(11):491. doi: 10.3390/tropicalmed8110491 (PMC10674593; doi:10.3390/tropicalmed8110491)
Supplement: Supplementary file 1 [file tropicalmed-08-00491-s001.zip › Suppl File S2.pdf]

I

(Legislative acts)

REGULATIONS

REGULATION (EU) 2016/679 OF THE EUROPEAN PARLIAMENT AND OF THE COUNCIL

of 27 April 2016

on the protection of natural persons with regard to the processing of personal data and on the free movement of such data, and repealing Directive 95/46/EC (General Data Protection Regulation)

(Text with EEA relevance)

THE EUROPEAN PARLIAMENT AND THE COUNCIL OF THE EUROPEAN UNION,

Having regard to the Treaty on the Functioning of the European Union, and in particular Article 16 thereof,

Having regard to the proposal from the European Commission,

After transmission of the draft legislative act to the national parliaments,

Having regard to the opinion of the European Economic and Social Committee ([1]),

Having regard to the opinion of the Committee of the Regions ([2]),

Acting in accordance with the ordinary legislative procedure ([3]),

Whereas:

(1) The protection of natural persons in relation to the processing of personal data is a fundamental right. Article 8(1) of the Charter of Fundamental Rights of the European Union (the 'Charter') and Article 16(1) of the Treaty on the Functioning of the European Union (TFEU) provide that everyone has the right to the protection of personal data concerning him or her.

(2) The principles of, and rules on the protection of natural persons with regard to the processing of their personal data should, whatever their nationality or residence, respect their fundamental rights and freedoms, in particular their right to the protection of personal data. This Regulation is intended to contribute to the accomplishment of an area of freedom, security and justice and of an economic union, to economic and social progress, to the strengthening and the convergence of the economies within the internal market, and to the well-being of natural persons.

(3) Directive 95/46/EC of the European Parliament and of the Council ([4]) seeks to harmonise the protection of fundamental rights and freedoms of natural persons in respect of processing activities and to ensure the free flow of personal data between Member States.

([1]) OJ C 229, 31.7.2012, p. 90.
([2]) OJ C 391, 18.12.2012, p. 127.
([3]) Position of the European Parliament of 12 March 2014 (not yet published in the Official Journal) and position of the Council at first reading of 8 April 2016 (not yet published in the Official Journal). Position of the European Parliament of 14 April 2016.
([4]) Directive 95/46/EC of the European Parliament and of the Council of 24 October 1995 on the protection of individuals with regard to the processing of personal data and on the free movement of such data (OJ L 281, 23.11.1995, p. 31).

(4) The processing of personal data should be designed to serve mankind. The right to the protection of personal data is not an absolute right; it must be considered in relation to its function in society and be balanced against other fundamental rights, in accordance with the principle of proportionality. This Regulation respects all fundamental rights and observes the freedoms and principles recognised in the Charter as enshrined in the Treaties, in particular the respect for private and family life, home and communications, the protection of personal data, freedom of thought, conscience and religion, freedom of expression and information, freedom to conduct a business, the right to an effective remedy and to a fair trial, and cultural, religious and linguistic diversity.

(5) The economic and social integration resulting from the functioning of the internal market has led to a substantial increase in cross-border flows of personal data. The exchange of personal data between public and private actors, including natural persons, associations and undertakings across the Union has increased. National authorities in the Member States are being called upon by Union law to cooperate and exchange personal data so as to be able to perform their duties or carry out tasks on behalf of an authority in another Member State.

(6) Rapid technological developments and globalisation have brought new challenges for the protection of personal data. The scale of the collection and sharing of personal data has increased significantly. Technology allows both private companies and public authorities to make use of personal data on an unprecedented scale in order to pursue their activities. Natural persons increasingly make personal information available publicly and globally. Technology has transformed both the economy and social life, and should further facilitate the free flow of personal data within the Union and the transfer to third countries and international organisations, while ensuring a high level of the protection of personal data.

(7) Those developments require a strong and more coherent data protection framework in the Union, backed by strong enforcement, given the importance of creating the trust that will allow the digital economy to develop across the internal market. Natural persons should have control of their own personal data. Legal and practical certainty for natural persons, economic operators and public authorities should be enhanced.

(8) Where this Regulation provides for specifications or restrictions of its rules by Member State law, Member States may, as far as necessary for coherence and for making the national provisions comprehensible to the persons to whom they apply, incorporate elements of this Regulation into their national law.

(9) The objectives and principles of Directive 95/46/EC remain sound, but it has not prevented fragmentation in the implementation of data protection across the Union, legal uncertainty or a widespread public perception that there are significant risks to the protection of natural persons, in particular with regard to online activity. Differences in the level of protection of the rights and freedoms of natural persons, in particular the right to the protection of personal data, with regard to the processing of personal data in the Member States may prevent the free flow of personal data throughout the Union. Those differences may therefore constitute an obstacle to the pursuit of economic activities at the level of the Union, distort competition and impede authorities in the discharge of their responsibilities under Union law. Such a difference in levels of protection is due to the existence of differences in the implementation and application of Directive 95/46/EC.

(10) In order to ensure a consistent and high level of protection of natural persons and to remove the obstacles to flows of personal data within the Union, the level of protection of the rights and freedoms of natural persons with regard to the processing of such data should be equivalent in all Member States. Consistent and homogenous application of the rules for the protection of the fundamental rights and freedoms of natural persons with regard to the processing of personal data should be ensured throughout the Union. Regarding the processing of personal data for compliance with a legal obligation, for the performance of a task carried out in the public interest or in the exercise of official authority vested in the controller, Member States should be allowed to maintain or introduce national provisions to further specify the application of the rules of this Regulation. In conjunction with the general and horizontal law on data protection implementing Directive 95/46/EC, Member States have several sector-specific laws in areas that need more specific provisions. This Regulation also provides a margin of manoeuvre for Member States to specify its rules, including for the processing of special categories of personal data ('sensitive data'). To that extent, this Regulation does not exclude Member State law that sets out the circumstances for specific processing situations, including determining more precisely the conditions under which the processing of personal data is lawful.

(11) Effective protection of personal data throughout the Union requires the strengthening and setting out in detail of the rights of data subjects and the obligations of those who process and determine the processing of personal data, as well as equivalent powers for monitoring and ensuring compliance with the rules for the protection of personal data and equivalent sanctions for infringements in the Member States.

(12) Article 16(2) TFEU mandates the European Parliament and the Council to lay down the rules relating to the protection of natural persons with regard to the processing of personal data and the rules relating to the free movement of personal data.

(13) In order to ensure a consistent level of protection for natural persons throughout the Union and to prevent divergences hampering the free movement of personal data within the internal market, a Regulation is necessary to provide legal certainty and transparency for economic operators, including micro, small and medium-sized enterprises, and to provide natural persons in all Member States with the same level of legally enforceable rights and obligations and responsibilities for controllers and processors, to ensure consistent monitoring of the processing of personal data, and equivalent sanctions in all Member States as well as effective cooperation between the supervisory authorities of different Member States. The proper functioning of the internal market requires that the free movement of personal data within the Union is not restricted or prohibited for reasons connected with the protection of natural persons with regard to the processing of personal data. To take account of the specific situation of micro, small and medium-sized enterprises, this Regulation includes a derogation for organisations with fewer than 250 employees with regard to record-keeping. In addition, the Union institutions and bodies, and Member States and their supervisory authorities, are encouraged to take account of the specific needs of micro, small and medium-sized enterprises in the application of this Regulation. The notion of micro, small and medium-sized enterprises should draw from Article 2 of the Annex to Commission Recommendation 2003/361/EC ([1]).

(14) The protection afforded by this Regulation should apply to natural persons, whatever their nationality or place of residence, in relation to the processing of their personal data. This Regulation does not cover the processing of personal data which concerns legal persons and in particular undertakings established as legal persons, including the name and the form of the legal person and the contact details of the legal person.

(15) In order to prevent creating a serious risk of circumvention, the protection of natural persons should be technologically neutral and should not depend on the techniques used. The protection of natural persons should apply to the processing of personal data by automated means, as well as to manual processing, if the personal data are contained or are intended to be contained in a filing system. Files or sets of files, as well as their cover pages, which are not structured according to specific criteria should not fall within the scope of this Regulation.

(16) This Regulation does not apply to issues of protection of fundamental rights and freedoms or the free flow of personal data related to activities which fall outside the scope of Union law, such as activities concerning national security. This Regulation does not apply to the processing of personal data by the Member States when carrying out activities in relation to the common foreign and security policy of the Union.

(17) Regulation (EC) No 45/2001 of the European Parliament and of the Council ([2]) applies to the processing of personal data by the Union institutions, bodies, offices and agencies. Regulation (EC) No 45/2001 and other Union legal acts applicable to such processing of personal data should be adapted to the principles and rules established in this Regulation and applied in the light of this Regulation. In order to provide a strong and coherent data protection framework in the Union, the necessary adaptations of Regulation (EC) No 45/2001 should follow after the adoption of this Regulation, in order to allow application at the same time as this Regulation.

(18) This Regulation does not apply to the processing of personal data by a natural person in the course of a purely personal or household activity and thus with no connection to a professional or commercial activity. Personal or

---

([1]) Commission Recommendation of 6 May 2003 concerning the definition of micro, small and medium-sized enterprises (C(2003) 1422) (OJ L 124, 20.5.2003, p. 36).

([2]) Regulation (EC) No 45/2001 of the European Parliament and of the Council of 18 December 2000 on the protection of individuals with regard to the processing of personal data by the Community institutions and bodies and on the free movement of such data (OJ L 8, 12.1.2001, p. 1).

household activities could include correspondence and the holding of addresses, or social networking and online activity undertaken within the context of such activities. However, this Regulation applies to controllers or processors which provide the means for processing personal data for such personal or household activities.

(19) The protection of natural persons with regard to the processing of personal data by competent authorities for the purposes of the prevention, investigation, detection or prosecution of criminal offences or the execution of criminal penalties, including the safeguarding against and the prevention of threats to public security and the free movement of such data, is the subject of a specific Union legal act. This Regulation should not, therefore, apply to processing activities for those purposes. However, personal data processed by public authorities under this Regulation should, when used for those purposes, be governed by a more specific Union legal act, namely Directive (EU) 2016/680 of the European Parliament and of the Council ([1]). Member States may entrust competent authorities within the meaning of Directive (EU) 2016/680 with tasks which are not necessarily carried out for the purposes of the prevention, investigation, detection or prosecution of criminal offences or the execution of criminal penalties, including the safeguarding against and prevention of threats to public security, so that the processing of personal data for those other purposes, in so far as it is within the scope of Union law, falls within the scope of this Regulation.

With regard to the processing of personal data by those competent authorities for purposes falling within scope of this Regulation, Member States should be able to maintain or introduce more specific provisions to adapt the application of the rules of this Regulation. Such provisions may determine more precisely specific requirements for the processing of personal data by those competent authorities for those other purposes, taking into account the constitutional, organisational and administrative structure of the respective Member State. When the processing of personal data by private bodies falls within the scope of this Regulation, this Regulation should provide for the possibility for Member States under specific conditions to restrict by law certain obligations and rights when such a restriction constitutes a necessary and proportionate measure in a democratic society to safeguard specific important interests including public security and the prevention, investigation, detection or prosecution of criminal offences or the execution of criminal penalties, including the safeguarding against and the prevention of threats to public security. This is relevant for instance in the framework of anti-money laundering or the activities of forensic laboratories.

(20) While this Regulation applies, inter alia, to the activities of courts and other judicial authorities, Union or Member State law could specify the processing operations and processing procedures in relation to the processing of personal data by courts and other judicial authorities. The competence of the supervisory authorities should not cover the processing of personal data when courts are acting in their judicial capacity, in order to safeguard the independence of the judiciary in the performance of its judicial tasks, including decision-making. It should be possible to entrust supervision of such data processing operations to specific bodies within the judicial system of the Member State, which should, in particular ensure compliance with the rules of this Regulation, enhance awareness among members of the judiciary of their obligations under this Regulation and handle complaints in relation to such data processing operations.

(21) This Regulation is without prejudice to the application of Directive 2000/31/EC of the European Parliament and of the Council ([2]), in particular of the liability rules of intermediary service providers in Articles 12 to 15 of that Directive. That Directive seeks to contribute to the proper functioning of the internal market by ensuring the free movement of information society services between Member States.

(22) Any processing of personal data in the context of the activities of an establishment of a controller or a processor in the Union should be carried out in accordance with this Regulation, regardless of whether the processing itself takes place within the Union. Establishment implies the effective and real exercise of activity through stable arrangements. The legal form of such arrangements, whether through a branch or a subsidiary with a legal personality, is not the determining factor in that respect.

([1]) Directive (EU) 2016/680 of the European Parliament and of the Council of 27 April 2016 on the protection of natural persons with regard to the processing of personal data by competent authorities for the purposes of prevention, investigation, detection or prosecution of criminal offences or the execution of criminal penalties, and the free movement of such data and repealing Council Framework Decision 2008/977/JHA (see page 89 of this Official Journal).

([2]) Directive 2000/31/EC of the European Parliament and of the Council of 8 June 2000 on certain legal aspects of information society services, in particular electronic commerce, in the Internal Market ('Directive on electronic commerce') (OJ L 178, 17.7.2000, p. 1).

(23) In order to ensure that natural persons are not deprived of the protection to which they are entitled under this Regulation, the processing of personal data of data subjects who are in the Union by a controller or a processor not established in the Union should be subject to this Regulation where the processing activities are related to offering goods or services to such data subjects irrespective of whether connected to a payment. In order to determine whether such a controller or processor is offering goods or services to data subjects who are in the Union, it should be ascertained whether it is apparent that the controller or processor envisages offering services to data subjects in one or more Member States in the Union. Whereas the mere accessibility of the controller's, processor's or an intermediary's website in the Union, of an email address or of other contact details, or the use of a language generally used in the third country where the controller is established, is insufficient to ascertain such intention, factors such as the use of a language or a currency generally used in one or more Member States with the possibility of ordering goods and services in that other language, or the mentioning of customers or users who are in the Union, may make it apparent that the controller envisages offering goods or services to data subjects in the Union.

(24) The processing of personal data of data subjects who are in the Union by a controller or processor not established in the Union should also be subject to this Regulation when it is related to the monitoring of the behaviour of such data subjects in so far as their behaviour takes place within the Union. In order to determine whether a processing activity can be considered to monitor the behaviour of data subjects, it should be ascertained whether natural persons are tracked on the internet including potential subsequent use of personal data processing techniques which consist of profiling a natural person, particularly in order to take decisions concerning her or him or for analysing or predicting her or his personal preferences, behaviours and attitudes.

(25) Where Member State law applies by virtue of public international law, this Regulation should also apply to a controller not established in the Union, such as in a Member State's diplomatic mission or consular post.

(26) The principles of data protection should apply to any information concerning an identified or identifiable natural person. Personal data which have undergone pseudonymisation, which could be attributed to a natural person by the use of additional information should be considered to be information on an identifiable natural person. To determine whether a natural person is identifiable, account should be taken of all the means reasonably likely to be used, such as singling out, either by the controller or by another person to identify the natural person directly or indirectly. To ascertain whether means are reasonably likely to be used to identify the natural person, account should be taken of all objective factors, such as the costs of and the amount of time required for identification, taking into consideration the available technology at the time of the processing and technological developments. The principles of data protection should therefore not apply to anonymous information, namely information which does not relate to an identified or identifiable natural person or to personal data rendered anonymous in such a manner that the data subject is not or no longer identifiable. This Regulation does not therefore concern the processing of such anonymous information, including for statistical or research purposes.

(27) This Regulation does not apply to the personal data of deceased persons. Member States may provide for rules regarding the processing of personal data of deceased persons.

(28) The application of pseudonymisation to personal data can reduce the risks to the data subjects concerned and help controllers and processors to meet their data-protection obligations. The explicit introduction of 'pseudonymisation' in this Regulation is not intended to preclude any other measures of data protection.

(29) In order to create incentives to apply pseudonymisation when processing personal data, measures of pseudonymisation should, whilst allowing general analysis, be possible within the same controller when that controller has taken technical and organisational measures necessary to ensure, for the processing concerned, that this Regulation is implemented, and that additional information for attributing the personal data to a specific data subject is kept separately. The controller processing the personal data should indicate the authorised persons within the same controller.

(30) Natural persons may be associated with online identifiers provided by their devices, applications, tools and protocols, such as internet protocol addresses, cookie identifiers or other identifiers such as radio frequency identification tags. This may leave traces which, in particular when combined with unique identifiers and other information received by the servers, may be used to create profiles of the natural persons and identify them.

(31) Public authorities to which personal data are disclosed in accordance with a legal obligation for the exercise of their official mission, such as tax and customs authorities, financial investigation units, independent administrative authorities, or financial market authorities responsible for the regulation and supervision of securities markets should not be regarded as recipients if they receive personal data which are necessary to carry out a particular inquiry in the general interest, in accordance with Union or Member State law. The requests for disclosure sent by the public authorities should always be in writing, reasoned and occasional and should not concern the entirety of a filing system or lead to the interconnection of filing systems. The processing of personal data by those public authorities should comply with the applicable data-protection rules according to the purposes of the processing.

(32) Consent should be given by a clear affirmative act establishing a freely given, specific, informed and unambiguous indication of the data subject's agreement to the processing of personal data relating to him or her, such as by a written statement, including by electronic means, or an oral statement. This could include ticking a box when visiting an internet website, choosing technical settings for information society services or another statement or conduct which clearly indicates in this context the data subject's acceptance of the proposed processing of his or her personal data. Silence, pre-ticked boxes or inactivity should not therefore constitute consent. Consent should cover all processing activities carried out for the same purpose or purposes. When the processing has multiple purposes, consent should be given for all of them. If the data subject's consent is to be given following a request by electronic means, the request must be clear, concise and not unnecessarily disruptive to the use of the service for which it is provided.

(33) It is often not possible to fully identify the purpose of personal data processing for scientific research purposes at the time of data collection. Therefore, data subjects should be allowed to give their consent to certain areas of scientific research when in keeping with recognised ethical standards for scientific research. Data subjects should have the opportunity to give their consent only to certain areas of research or parts of research projects to the extent allowed by the intended purpose.

(34) Genetic data should be defined as personal data relating to the inherited or acquired genetic characteristics of a natural person which result from the analysis of a biological sample from the natural person in question, in particular chromosomal, deoxyribonucleic acid (DNA) or ribonucleic acid (RNA) analysis, or from the analysis of another element enabling equivalent information to be obtained.

(35) Personal data concerning health should include all data pertaining to the health status of a data subject which reveal information relating to the past, current or future physical or mental health status of the data subject. This includes information about the natural person collected in the course of the registration for, or the provision of, health care services as referred to in Directive 2011/24/EU of the European Parliament and of the Council ([1]) to that natural person; a number, symbol or particular assigned to a natural person to uniquely identify the natural person for health purposes; information derived from the testing or examination of a body part or bodily substance, including from genetic data and biological samples; and any information on, for example, a disease, disability, disease risk, medical history, clinical treatment or the physiological or biomedical state of the data subject independent of its source, for example from a physician or other health professional, a hospital, a medical device or an in vitro diagnostic test.

(36) The main establishment of a controller in the Union should be the place of its central administration in the Union, unless the decisions on the purposes and means of the processing of personal data are taken in another establishment of the controller in the Union, in which case that other establishment should be considered to be

([1]) Directive 2011/24/EU of the European Parliament and of the Council of 9 March 2011 on the application of patients' rights in cross-border healthcare (OJ L 88, 4.4.2011, p. 45).

the main establishment. The main establishment of a controller in the Union should be determined according to objective criteria and should imply the effective and real exercise of management activities determining the main decisions as to the purposes and means of processing through stable arrangements. That criterion should not depend on whether the processing of personal data is carried out at that location. The presence and use of technical means and technologies for processing personal data or processing activities do not, in themselves, constitute a main establishment and are therefore not determining criteria for a main establishment. The main establishment of the processor should be the place of its central administration in the Union or, if it has no central administration in the Union, the place where the main processing activities take place in the Union. In cases involving both the controller and the processor, the competent lead supervisory authority should remain the supervisory authority of the Member State where the controller has its main establishment, but the supervisory authority of the processor should be considered to be a supervisory authority concerned and that supervisory authority should participate in the cooperation procedure provided for by this Regulation. In any case, the supervisory authorities of the Member State or Member States where the processor has one or more establishments should not be considered to be supervisory authorities concerned where the draft decision concerns only the controller. Where the processing is carried out by a group of undertakings, the main establishment of the controlling undertaking should be considered to be the main establishment of the group of undertakings, except where the purposes and means of processing are determined by another undertaking.

(37) A group of undertakings should cover a controlling undertaking and its controlled undertakings, whereby the controlling undertaking should be the undertaking which can exert a dominant influence over the other undertakings by virtue, for example, of ownership, financial participation or the rules which govern it or the power to have personal data protection rules implemented. An undertaking which controls the processing of personal data in undertakings affiliated to it should be regarded, together with those undertakings, as a group of undertakings.

(38) Children merit specific protection with regard to their personal data, as they may be less aware of the risks, consequences and safeguards concerned and their rights in relation to the processing of personal data. Such specific protection should, in particular, apply to the use of personal data of children for the purposes of marketing or creating personality or user profiles and the collection of personal data with regard to children when using services offered directly to a child. The consent of the holder of parental responsibility should not be necessary in the context of preventive or counselling services offered directly to a child.

(39) Any processing of personal data should be lawful and fair. It should be transparent to natural persons that personal data concerning them are collected, used, consulted or otherwise processed and to what extent the personal data are or will be processed. The principle of transparency requires that any information and communication relating to the processing of those personal data be easily accessible and easy to understand, and that clear and plain language be used. That principle concerns, in particular, information to the data subjects on the identity of the controller and the purposes of the processing and further information to ensure fair and transparent processing in respect of the natural persons concerned and their right to obtain confirmation and communication of personal data concerning them which are being processed. Natural persons should be made aware of risks, rules, safeguards and rights in relation to the processing of personal data and how to exercise their rights in relation to such processing. In particular, the specific purposes for which personal data are processed should be explicit and legitimate and determined at the time of the collection of the personal data. The personal data should be adequate, relevant and limited to what is necessary for the purposes for which they are processed. This requires, in particular, ensuring that the period for which the personal data are stored is limited to a strict minimum. Personal data should be processed only if the purpose of the processing could not reasonably be fulfilled by other means. In order to ensure that the personal data are not kept longer than necessary, time limits should be established by the controller for erasure or for a periodic review. Every reasonable step should be taken to ensure that personal data which are inaccurate are rectified or deleted. Personal data should be processed in a manner that ensures appropriate security and confidentiality of the personal data, including for preventing unauthorised access to or use of personal data and the equipment used for the processing.

(40) In order for processing to be lawful, personal data should be processed on the basis of the consent of the data subject concerned or some other legitimate basis, laid down by law, either in this Regulation or in other Union or

Member State law as referred to in this Regulation, including the necessity for compliance with the legal obligation to which the controller is subject or the necessity for the performance of a contract to which the data subject is party or in order to take steps at the request of the data subject prior to entering into a contract.

(41) Where this Regulation refers to a legal basis or a legislative measure, this does not necessarily require a legislative act adopted by a parliament, without prejudice to requirements pursuant to the constitutional order of the Member State concerned. However, such a legal basis or legislative measure should be clear and precise and its application should be foreseeable to persons subject to it, in accordance with the case-law of the Court of Justice of the European Union (the 'Court of Justice') and the European Court of Human Rights.

(42) Where processing is based on the data subject's consent, the controller should be able to demonstrate that the data subject has given consent to the processing operation. In particular in the context of a written declaration on another matter, safeguards should ensure that the data subject is aware of the fact that and the extent to which consent is given. In accordance with Council Directive 93/13/EEC (1) a declaration of consent pre-formulated by the controller should be provided in an intelligible and easily accessible form, using clear and plain language and it should not contain unfair terms. For consent to be informed, the data subject should be aware at least of the identity of the controller and the purposes of the processing for which the personal data are intended. Consent should not be regarded as freely given if the data subject has no genuine or free choice or is unable to refuse or withdraw consent without detriment.

(43) In order to ensure that consent is freely given, consent should not provide a valid legal ground for the processing of personal data in a specific case where there is a clear imbalance between the data subject and the controller, in particular where the controller is a public authority and it is therefore unlikely that consent was freely given in all the circumstances of that specific situation. Consent is presumed not to be freely given if it does not allow separate consent to be given to different personal data processing operations despite it being appropriate in the individual case, or if the performance of a contract, including the provision of a service, is dependent on the consent despite such consent not being necessary for such performance.

(44) Processing should be lawful where it is necessary in the context of a contract or the intention to enter into a contract.

(45) Where processing is carried out in accordance with a legal obligation to which the controller is subject or where processing is necessary for the performance of a task carried out in the public interest or in the exercise of official authority, the processing should have a basis in Union or Member State law. This Regulation does not require a specific law for each individual processing. A law as a basis for several processing operations based on a legal obligation to which the controller is subject or where processing is necessary for the performance of a task carried out in the public interest or in the exercise of an official authority may be sufficient. It should also be for Union or Member State law to determine the purpose of processing. Furthermore, that law could specify the general conditions of this Regulation governing the lawfulness of personal data processing, establish specifications for determining the controller, the type of personal data which are subject to the processing, the data subjects concerned, the entities to which the personal data may be disclosed, the purpose limitations, the storage period and other measures to ensure lawful and fair processing. It should also be for Union or Member State law to determine whether the controller performing a task carried out in the public interest or in the exercise of official authority should be a public authority or another natural or legal person governed by public law, or, where it is in the public interest to do so, including for health purposes such as public health and social protection and the management of health care services, by private law, such as a professional association.

(46) The processing of personal data should also be regarded to be lawful where it is necessary to protect an interest which is essential for the life of the data subject or that of another natural person. Processing of personal data

(1) Council Directive 93/13/EEC of 5 April 1993 on unfair terms in consumer contracts (OJ L 95, 21.4.1993, p. 29).

based on the vital interest of another natural person should in principle take place only where the processing cannot be manifestly based on another legal basis. Some types of processing may serve both important grounds of public interest and the vital interests of the data subject as for instance when processing is necessary for humanitarian purposes, including for monitoring epidemics and their spread or in situations of humanitarian emergencies, in particular in situations of natural and man-made disasters.

(47) The legitimate interests of a controller, including those of a controller to which the personal data may be disclosed, or of a third party, may provide a legal basis for processing, provided that the interests or the fundamental rights and freedoms of the data subject are not overriding, taking into consideration the reasonable expectations of data subjects based on their relationship with the controller. Such legitimate interest could exist for example where there is a relevant and appropriate relationship between the data subject and the controller in situations such as where the data subject is a client or in the service of the controller. At any rate the existence of a legitimate interest would need careful assessment including whether a data subject can reasonably expect at the time and in the context of the collection of the personal data that processing for that purpose may take place. The interests and fundamental rights of the data subject could in particular override the interest of the data controller where personal data are processed in circumstances where data subjects do not reasonably expect further processing. Given that it is for the legislator to provide by law for the legal basis for public authorities to process personal data, that legal basis should not apply to the processing by public authorities in the performance of their tasks. The processing of personal data strictly necessary for the purposes of preventing fraud also constitutes a legitimate interest of the data controller concerned. The processing of personal data for direct marketing purposes may be regarded as carried out for a legitimate interest.

(48) Controllers that are part of a group of undertakings or institutions affiliated to a central body may have a legitimate interest in transmitting personal data within the group of undertakings for internal administrative purposes, including the processing of clients' or employees' personal data. The general principles for the transfer of personal data, within a group of undertakings, to an undertaking located in a third country remain unaffected.

(49) The processing of personal data to the extent strictly necessary and proportionate for the purposes of ensuring network and information security, i.e. the ability of a network or an information system to resist, at a given level of confidence, accidental events or unlawful or malicious actions that compromise the availability, authenticity, integrity and confidentiality of stored or transmitted personal data, and the security of the related services offered by, or accessible via, those networks and systems, by public authorities, by computer emergency response teams (CERTs), computer security incident response teams (CSIRTs), by providers of electronic communications networks and services and by providers of security technologies and services, constitutes a legitimate interest of the data controller concerned. This could, for example, include preventing unauthorised access to electronic communications networks and malicious code distribution and stopping 'denial of service' attacks and damage to computer and electronic communication systems.

(50) The processing of personal data for purposes other than those for which the personal data were initially collected should be allowed only where the processing is compatible with the purposes for which the personal data were initially collected. In such a case, no legal basis separate from that which allowed the collection of the personal data is required. If the processing is necessary for the performance of a task carried out in the public interest or in the exercise of official authority vested in the controller, Union or Member State law may determine and specify the tasks and purposes for which the further processing should be regarded as compatible and lawful. Further processing for archiving purposes in the public interest, scientific or historical research purposes or statistical purposes should be considered to be compatible lawful processing operations. The legal basis provided by Union or Member State law for the processing of personal data may also provide a legal basis for further processing. In order to ascertain whether a purpose of further processing is compatible with the purpose for which the personal data are initially collected, the controller, after having met all the requirements for the lawfulness of the original processing, should take into account, inter alia: any link between those purposes and the purposes of the intended further processing; the context in which the personal data have been collected, in particular the reasonable expectations of data subjects based on their relationship with the controller as to their

further use; the nature of the personal data; the consequences of the intended further processing for data subjects; and the existence of appropriate safeguards in both the original and intended further processing operations.

Where the data subject has given consent or the processing is based on Union or Member State law which constitutes a necessary and proportionate measure in a democratic society to safeguard, in particular, important objectives of general public interest, the controller should be allowed to further process the personal data irrespective of the compatibility of the purposes. In any case, the application of the principles set out in this Regulation and in particular the information of the data subject on those other purposes and on his or her rights including the right to object, should be ensured. Indicating possible criminal acts or threats to public security by the controller and transmitting the relevant personal data in individual cases or in several cases relating to the same criminal act or threats to public security to a competent authority should be regarded as being in the legitimate interest pursued by the controller. However, such transmission in the legitimate interest of the controller or further processing of personal data should be prohibited if the processing is not compatible with a legal, professional or other binding obligation of secrecy.

(51) Personal data which are, by their nature, particularly sensitive in relation to fundamental rights and freedoms merit specific protection as the context of their processing could create significant risks to the fundamental rights and freedoms. Those personal data should include personal data revealing racial or ethnic origin, whereby the use of the term 'racial origin' in this Regulation does not imply an acceptance by the Union of theories which attempt to determine the existence of separate human races. The processing of photographs should not systematically be considered to be processing of special categories of personal data as they are covered by the definition of biometric data only when processed through a specific technical means allowing the unique identification or authentication of a natural person. Such personal data should not be processed, unless processing is allowed in specific cases set out in this Regulation, taking into account that Member States law may lay down specific provisions on data protection in order to adapt the application of the rules of this Regulation for compliance with a legal obligation or for the performance of a task carried out in the public interest or in the exercise of official authority vested in the controller. In addition to the specific requirements for such processing, the general principles and other rules of this Regulation should apply, in particular as regards the conditions for lawful processing. Derogations from the general prohibition for processing such special categories of personal data should be explicitly provided, inter alia, where the data subject gives his or her explicit consent or in respect of specific needs in particular where the processing is carried out in the course of legitimate activities by certain associations or foundations the purpose of which is to permit the exercise of fundamental freedoms.

(52) Derogating from the prohibition on processing special categories of personal data should also be allowed when provided for in Union or Member State law and subject to suitable safeguards, so as to protect personal data and other fundamental rights, where it is in the public interest to do so, in particular processing personal data in the field of employment law, social protection law including pensions and for health security, monitoring and alert purposes, the prevention or control of communicable diseases and other serious threats to health. Such a derogation may be made for health purposes, including public health and the management of health-care services, especially in order to ensure the quality and cost-effectiveness of the procedures used for settling claims for benefits and services in the health insurance system, or for archiving purposes in the public interest, scientific or historical research purposes or statistical purposes. A derogation should also allow the processing of such personal data where necessary for the establishment, exercise or defence of legal claims, whether in court proceedings or in an administrative or out-of-court procedure.

(53) Special categories of personal data which merit higher protection should be processed for health-related purposes only where necessary to achieve those purposes for the benefit of natural persons and society as a whole, in particular in the context of the management of health or social care services and systems, including processing by the management and central national health authorities of such data for the purpose of quality control, management information and the general national and local supervision of the health or social care system, and ensuring continuity of health or social care and cross-border healthcare or health security, monitoring and alert purposes, or for archiving purposes in the public interest, scientific or historical research purposes or statistical purposes, based on Union or Member State law which has to meet an objective of public interest, as well as for studies conducted in the public interest in the area of public health. Therefore, this Regulation should provide for harmonised conditions for the processing of special categories of personal data concerning health, in respect of specific needs, in particular where the processing of such data is carried out for certain health-related purposes

by persons subject to a legal obligation of professional secrecy. Union or Member State law should provide for specific and suitable measures so as to protect the fundamental rights and the personal data of natural persons. Member States should be allowed to maintain or introduce further conditions, including limitations, with regard to the processing of genetic data, biometric data or data concerning health. However, this should not hamper the free flow of personal data within the Union when those conditions apply to cross-border processing of such data.

(54) The processing of special categories of personal data may be necessary for reasons of public interest in the areas of public health without consent of the data subject. Such processing should be subject to suitable and specific measures so as to protect the rights and freedoms of natural persons. In that context, 'public health' should be interpreted as defined in Regulation (EC) No 1338/2008 of the European Parliament and of the Council ([1]), namely all elements related to health, namely health status, including morbidity and disability, the determinants having an effect on that health status, health care needs, resources allocated to health care, the provision of, and universal access to, health care as well as health care expenditure and financing, and the causes of mortality. Such processing of data concerning health for reasons of public interest should not result in personal data being processed for other purposes by third parties such as employers or insurance and banking companies.

(55) Moreover, the processing of personal data by official authorities for the purpose of achieving the aims, laid down by constitutional law or by international public law, of officially recognised religious associations, is carried out on grounds of public interest.

(56) Where in the course of electoral activities, the operation of the democratic system in a Member State requires that political parties compile personal data on people's political opinions, the processing of such data may be permitted for reasons of public interest, provided that appropriate safeguards are established.

(57) If the personal data processed by a controller do not permit the controller to identify a natural person, the data controller should not be obliged to acquire additional information in order to identify the data subject for the sole purpose of complying with any provision of this Regulation. However, the controller should not refuse to take additional information provided by the data subject in order to support the exercise of his or her rights. Identification should include the digital identification of a data subject, for example through authentication mechanism such as the same credentials, used by the data subject to log-in to the on-line service offered by the data controller.

(58) The principle of transparency requires that any information addressed to the public or to the data subject be concise, easily accessible and easy to understand, and that clear and plain language and, additionally, where appropriate, visualisation be used. Such information could be provided in electronic form, for example, when addressed to the public, through a website. This is of particular relevance in situations where the proliferation of actors and the technological complexity of practice make it difficult for the data subject to know and understand whether, by whom and for what purpose personal data relating to him or her are being collected, such as in the case of online advertising. Given that children merit specific protection, any information and communication, where processing is addressed to a child, should be in such a clear and plain language that the child can easily understand.

(59) Modalities should be provided for facilitating the exercise of the data subject's rights under this Regulation, including mechanisms to request and, if applicable, obtain, free of charge, in particular, access to and rectification or erasure of personal data and the exercise of the right to object. The controller should also provide means for requests to be made electronically, especially where personal data are processed by electronic means. The controller should be obliged to respond to requests from the data subject without undue delay and at the latest within one month and to give reasons where the controller does not intend to comply with any such requests.

([1]) Regulation (EC) No 1338/2008 of the European Parliament and of the Council of 16 December 2008 on Community statistics on public health and health and safety at work (OJ L 354, 31.12.2008, p. 70).

(60) The principles of fair and transparent processing require that the data subject be informed of the existence of the processing operation and its purposes. The controller should provide the data subject with any further information necessary to ensure fair and transparent processing taking into account the specific circumstances and context in which the personal data are processed. Furthermore, the data subject should be informed of the existence of profiling and the consequences of such profiling. Where the personal data are collected from the data subject, the data subject should also be informed whether he or she is obliged to provide the personal data and of the consequences, where he or she does not provide such data. That information may be provided in combination with standardised icons in order to give in an easily visible, intelligible and clearly legible manner, a meaningful overview of the intended processing. Where the icons are presented electronically, they should be machine-readable.

(61) The information in relation to the processing of personal data relating to the data subject should be given to him or her at the time of collection from the data subject, or, where the personal data are obtained from another source, within a reasonable period, depending on the circumstances of the case. Where personal data can be legitimately disclosed to another recipient, the data subject should be informed when the personal data are first disclosed to the recipient. Where the controller intends to process the personal data for a purpose other than that for which they were collected, the controller should provide the data subject prior to that further processing with information on that other purpose and other necessary information. Where the origin of the personal data cannot be provided to the data subject because various sources have been used, general information should be provided.

(62) However, it is not necessary to impose the obligation to provide information where the data subject already possesses the information, where the recording or disclosure of the personal data is expressly laid down by law or where the provision of information to the data subject proves to be impossible or would involve a disproportionate effort. The latter could in particular be the case where processing is carried out for archiving purposes in the public interest, scientific or historical research purposes or statistical purposes. In that regard, the number of data subjects, the age of the data and any appropriate safeguards adopted should be taken into consideration.

(63) A data subject should have the right of access to personal data which have been collected concerning him or her, and to exercise that right easily and at reasonable intervals, in order to be aware of, and verify, the lawfulness of the processing. This includes the right for data subjects to have access to data concerning their health, for example the data in their medical records containing information such as diagnoses, examination results, assessments by treating physicians and any treatment or interventions provided. Every data subject should therefore have the right to know and obtain communication in particular with regard to the purposes for which the personal data are processed, where possible the period for which the personal data are processed, the recipients of the personal data, the logic involved in any automatic personal data processing and, at least when based on profiling, the consequences of such processing. Where possible, the controller should be able to provide remote access to a secure system which would provide the data subject with direct access to his or her personal data. That right should not adversely affect the rights or freedoms of others, including trade secrets or intellectual property and in particular the copyright protecting the software. However, the result of those considerations should not be a refusal to provide all information to the data subject. Where the controller processes a large quantity of information concerning the data subject, the controller should be able to request that, before the information is delivered, the data subject specify the information or processing activities to which the request relates.

(64) The controller should use all reasonable measures to verify the identity of a data subject who requests access, in particular in the context of online services and online identifiers. A controller should not retain personal data for the sole purpose of being able to react to potential requests.

(65) A data subject should have the right to have personal data concerning him or her rectified and a 'right to be forgotten' where the retention of such data infringes this Regulation or Union or Member State law to which the controller is subject. In particular, a data subject should have the right to have his or her personal data erased and no longer processed where the personal data are no longer necessary in relation to the purposes for which they are collected or otherwise processed, where a data subject has withdrawn his or her consent or objects to the processing of personal data concerning him or her, or where the processing of his or her personal data does not otherwise comply with this Regulation. That right is relevant in particular where the data subject has given

his or her consent as a child and is not fully aware of the risks involved by the processing, and later wants to remove such personal data, especially on the internet. The data subject should be able to exercise that right notwithstanding the fact that he or she is no longer a child. However, the further retention of the personal data should be lawful where it is necessary, for exercising the right of freedom of expression and information, for compliance with a legal obligation, for the performance of a task carried out in the public interest or in the exercise of official authority vested in the controller, on the grounds of public interest in the area of public health, for archiving purposes in the public interest, scientific or historical research purposes or statistical purposes, or for the establishment, exercise or defence of legal claims.

(66) To strengthen the right to be forgotten in the online environment, the right to erasure should also be extended in such a way that a controller who has made the personal data public should be obliged to inform the controllers which are processing such personal data to erase any links to, or copies or replications of those personal data. In doing so, that controller should take reasonable steps, taking into account available technology and the means available to the controller, including technical measures, to inform the controllers which are processing the personal data of the data subject's request.

(67) Methods by which to restrict the processing of personal data could include, inter alia, temporarily moving the selected data to another processing system, making the selected personal data unavailable to users, or temporarily removing published data from a website. In automated filing systems, the restriction of processing should in principle be ensured by technical means in such a manner that the personal data are not subject to further processing operations and cannot be changed. The fact that the processing of personal data is restricted should be clearly indicated in the system.

(68) To further strengthen the control over his or her own data, where the processing of personal data is carried out by automated means, the data subject should also be allowed to receive personal data concerning him or her which he or she has provided to a controller in a structured, commonly used, machine-readable and interoperable format, and to transmit it to another controller. Data controllers should be encouraged to develop interoperable formats that enable data portability. That right should apply where the data subject provided the personal data on the basis of his or her consent or the processing is necessary for the performance of a contract. It should not apply where processing is based on a legal ground other than consent or contract. By its very nature, that right should not be exercised against controllers processing personal data in the exercise of their public duties. It should therefore not apply where the processing of the personal data is necessary for compliance with a legal obligation to which the controller is subject or for the performance of a task carried out in the public interest or in the exercise of an official authority vested in the controller. The data subject's right to transmit or receive personal data concerning him or her should not create an obligation for the controllers to adopt or maintain processing systems which are technically compatible. Where, in a certain set of personal data, more than one data subject is concerned, the right to receive the personal data should be without prejudice to the rights and freedoms of other data subjects in accordance with this Regulation. Furthermore, that right should not prejudice the right of the data subject to obtain the erasure of personal data and the limitations of that right as set out in this Regulation and should, in particular, not imply the erasure of personal data concerning the data subject which have been provided by him or her for the performance of a contract to the extent that and for as long as the personal data are necessary for the performance of that contract. Where technically feasible, the data subject should have the right to have the personal data transmitted directly from one controller to another.

(69) Where personal data might lawfully be processed because processing is necessary for the performance of a task carried out in the public interest or in the exercise of official authority vested in the controller, or on grounds of the legitimate interests of a controller or a third party, a data subject should, nevertheless, be entitled to object to the processing of any personal data relating to his or her particular situation. It should be for the controller to demonstrate that its compelling legitimate interest overrides the interests or the fundamental rights and freedoms of the data subject.

(70) Where personal data are processed for the purposes of direct marketing, the data subject should have the right to object to such processing, including profiling to the extent that it is related to such direct marketing, whether with regard to initial or further processing, at any time and free of charge. That right should be explicitly brought to the attention of the data subject and presented clearly and separately from any other information.

(71) The data subject should have the right not to be subject to a decision, which may include a measure, evaluating personal aspects relating to him or her which is based solely on automated processing and which produces legal effects concerning him or her or similarly significantly affects him or her, such as automatic refusal of an online credit application or e-recruiting practices without any human intervention. Such processing includes 'profiling' that consists of any form of automated processing of personal data evaluating the personal aspects relating to a natural person, in particular to analyse or predict aspects concerning the data subject's performance at work, economic situation, health, personal preferences or interests, reliability or behaviour, location or movements, where it produces legal effects concerning him or her or similarly significantly affects him or her. However, decision-making based on such processing, including profiling, should be allowed where expressly authorised by Union or Member State law to which the controller is subject, including for fraud and tax-evasion monitoring and prevention purposes conducted in accordance with the regulations, standards and recommendations of Union institutions or national oversight bodies and to ensure the security and reliability of a service provided by the controller, or necessary for the entering or performance of a contract between the data subject and a controller, or when the data subject has given his or her explicit consent. In any case, such processing should be subject to suitable safeguards, which should include specific information to the data subject and the right to obtain human intervention, to express his or her point of view, to obtain an explanation of the decision reached after such assessment and to challenge the decision. Such measure should not concern a child.

In order to ensure fair and transparent processing in respect of the data subject, taking into account the specific circumstances and context in which the personal data are processed, the controller should use appropriate mathematical or statistical procedures for the profiling, implement technical and organisational measures appropriate to ensure, in particular, that factors which result in inaccuracies in personal data are corrected and the risk of errors is minimised, secure personal data in a manner that takes account of the potential risks involved for the interests and rights of the data subject and that prevents, inter alia, discriminatory effects on natural persons on the basis of racial or ethnic origin, political opinion, religion or beliefs, trade union membership, genetic or health status or sexual orientation, or that result in measures having such an effect. Automated decision-making and profiling based on special categories of personal data should be allowed only under specific conditions.

(72) Profiling is subject to the rules of this Regulation governing the processing of personal data, such as the legal grounds for processing or data protection principles. The European Data Protection Board established by this Regulation (the 'Board') should be able to issue guidance in that context.

(73) Restrictions concerning specific principles and the rights of information, access to and rectification or erasure of personal data, the right to data portability, the right to object, decisions based on profiling, as well as the communication of a personal data breach to a data subject and certain related obligations of the controllers may be imposed by Union or Member State law, as far as necessary and proportionate in a democratic society to safeguard public security, including the protection of human life especially in response to natural or manmade disasters, the prevention, investigation and prosecution of criminal offences or the execution of criminal penalties, including the safeguarding against and the prevention of threats to public security, or of breaches of ethics for regulated professions, other important objectives of general public interest of the Union or of a Member State, in particular an important economic or financial interest of the Union or of a Member State, the keeping of public registers kept for reasons of general public interest, further processing of archived personal data to provide specific information related to the political behaviour under former totalitarian state regimes or the protection of the data subject or the rights and freedoms of others, including social protection, public health and humanitarian purposes. Those restrictions should be in accordance with the requirements set out in the Charter and in the European Convention for the Protection of Human Rights and Fundamental Freedoms.

(74) The responsibility and liability of the controller for any processing of personal data carried out by the controller or on the controller's behalf should be established. In particular, the controller should be obliged to implement appropriate and effective measures and be able to demonstrate the compliance of processing activities with this Regulation, including the effectiveness of the measures. Those measures should take into account the nature, scope, context and purposes of the processing and the risk to the rights and freedoms of natural persons.

(75) The risk to the rights and freedoms of natural persons, of varying likelihood and severity, may result from personal data processing which could lead to physical, material or non-material damage, in particular: where the processing may give rise to discrimination, identity theft or fraud, financial loss, damage to the reputation, loss of confidentiality of personal data protected by professional secrecy, unauthorised reversal of pseudonymisation, or any other significant economic or social disadvantage; where data subjects might be deprived of their rights and freedoms or prevented from exercising control over their personal data; where personal data are processed which reveal racial or ethnic origin, political opinions, religion or philosophical beliefs, trade union membership, and the processing of genetic data, data concerning health or data concerning sex life or criminal convictions and offences or related security measures; where personal aspects are evaluated, in particular analysing or predicting aspects concerning performance at work, economic situation, health, personal preferences or interests, reliability or behaviour, location or movements, in order to create or use personal profiles; where personal data of vulnerable natural persons, in particular of children, are processed; or where processing involves a large amount of personal data and affects a large number of data subjects.

(76) The likelihood and severity of the risk to the rights and freedoms of the data subject should be determined by reference to the nature, scope, context and purposes of the processing. Risk should be evaluated on the basis of an objective assessment, by which it is established whether data processing operations involve a risk or a high risk.

(77) Guidance on the implementation of appropriate measures and on the demonstration of compliance by the controller or the processor, especially as regards the identification of the risk related to the processing, their assessment in terms of origin, nature, likelihood and severity, and the identification of best practices to mitigate the risk, could be provided in particular by means of approved codes of conduct, approved certifications, guidelines provided by the Board or indications provided by a data protection officer. The Board may also issue guidelines on processing operations that are considered to be unlikely to result in a high risk to the rights and freedoms of natural persons and indicate what measures may be sufficient in such cases to address such risk.

(78) The protection of the rights and freedoms of natural persons with regard to the processing of personal data require that appropriate technical and organisational measures be taken to ensure that the requirements of this Regulation are met. In order to be able to demonstrate compliance with this Regulation, the controller should adopt internal policies and implement measures which meet in particular the principles of data protection by design and data protection by default. Such measures could consist, inter alia, of minimising the processing of personal data, pseudonymising personal data as soon as possible, transparency with regard to the functions and processing of personal data, enabling the data subject to monitor the data processing, enabling the controller to create and improve security features. When developing, designing, selecting and using applications, services and products that are based on the processing of personal data or process personal data to fulfil their task, producers of the products, services and applications should be encouraged to take into account the right to data protection when developing and designing such products, services and applications and, with due regard to the state of the art, to make sure that controllers and processors are able to fulfil their data protection obligations. The principles of data protection by design and by default should also be taken into consideration in the context of public tenders.

(79) The protection of the rights and freedoms of data subjects as well as the responsibility and liability of controllers and processors, also in relation to the monitoring by and measures of supervisory authorities, requires a clear allocation of the responsibilities under this Regulation, including where a controller determines the purposes and means of the processing jointly with other controllers or where a processing operation is carried out on behalf of a controller.

(80) Where a controller or a processor not established in the Union is processing personal data of data subjects who are in the Union whose processing activities are related to the offering of goods or services, irrespective of whether a payment of the data subject is required, to such data subjects in the Union, or to the monitoring of their behaviour as far as their behaviour takes place within the Union, the controller or the processor should designate a representative, unless the processing is occasional, does not include processing, on a large scale, of special categories of personal data or the processing of personal data relating to criminal convictions and offences, and is unlikely to result in a risk to the rights and freedoms of natural persons, taking into account the

nature, context, scope and purposes of the processing or if the controller is a public authority or body. The representative should act on behalf of the controller or the processor and may be addressed by any supervisory authority. The representative should be explicitly designated by a written mandate of the controller or of the processor to act on its behalf with regard to its obligations under this Regulation. The designation of such a representative does not affect the responsibility or liability of the controller or of the processor under this Regulation. Such a representative should perform its tasks according to the mandate received from the controller or processor, including cooperating with the competent supervisory authorities with regard to any action taken to ensure compliance with this Regulation. The designated representative should be subject to enforcement proceedings in the event of non-compliance by the controller or processor.

(81) To ensure compliance with the requirements of this Regulation in respect of the processing to be carried out by the processor on behalf of the controller, when entrusting a processor with processing activities, the controller should use only processors providing sufficient guarantees, in particular in terms of expert knowledge, reliability and resources, to implement technical and organisational measures which will meet the requirements of this Regulation, including for the security of processing. The adherence of the processor to an approved code of conduct or an approved certification mechanism may be used as an element to demonstrate compliance with the obligations of the controller. The carrying-out of processing by a processor should be governed by a contract or other legal act under Union or Member State law, binding the processor to the controller, setting out the subject-matter and duration of the processing, the nature and purposes of the processing, the type of personal data and categories of data subjects, taking into account the specific tasks and responsibilities of the processor in the context of the processing to be carried out and the risk to the rights and freedoms of the data subject. The controller and processor may choose to use an individual contract or standard contractual clauses which are adopted either directly by the Commission or by a supervisory authority in accordance with the consistency mechanism and then adopted by the Commission. After the completion of the processing on behalf of the controller, the processor should, at the choice of the controller, return or delete the personal data, unless there is a requirement to store the personal data under Union or Member State law to which the processor is subject.

(82) In order to demonstrate compliance with this Regulation, the controller or processor should maintain records of processing activities under its responsibility. Each controller and processor should be obliged to cooperate with the supervisory authority and make those records, on request, available to it, so that it might serve for monitoring those processing operations.

(83) In order to maintain security and to prevent processing in infringement of this Regulation, the controller or processor should evaluate the risks inherent in the processing and implement measures to mitigate those risks, such as encryption. Those measures should ensure an appropriate level of security, including confidentiality, taking into account the state of the art and the costs of implementation in relation to the risks and the nature of the personal data to be protected. In assessing data security risk, consideration should be given to the risks that are presented by personal data processing, such as accidental or unlawful destruction, loss, alteration, unauthorised disclosure of, or access to, personal data transmitted, stored or otherwise processed which may in particular lead to physical, material or non-material damage.

(84) In order to enhance compliance with this Regulation where processing operations are likely to result in a high risk to the rights and freedoms of natural persons, the controller should be responsible for the carrying-out of a data protection impact assessment to evaluate, in particular, the origin, nature, particularity and severity of that risk. The outcome of the assessment should be taken into account when determining the appropriate measures to be taken in order to demonstrate that the processing of personal data complies with this Regulation. Where a data-protection impact assessment indicates that processing operations involve a high risk which the controller cannot mitigate by appropriate measures in terms of available technology and costs of implementation, a consultation of the supervisory authority should take place prior to the processing.

(85) A personal data breach may, if not addressed in an appropriate and timely manner, result in physical, material or non-material damage to natural persons such as loss of control over their personal data or limitation of their rights, discrimination, identity theft or fraud, financial loss, unauthorised reversal of pseudonymisation, damage to reputation, loss of confidentiality of personal data protected by professional secrecy or any other significant economic or social disadvantage to the natural person concerned. Therefore, as soon as the controller becomes

aware that a personal data breach has occurred, the controller should notify the personal data breach to the supervisory authority without undue delay and, where feasible, not later than 72 hours after having become aware of it, unless the controller is able to demonstrate, in accordance with the accountability principle, that the personal data breach is unlikely to result in a risk to the rights and freedoms of natural persons. Where such notification cannot be achieved within 72 hours, the reasons for the delay should accompany the notification and information may be provided in phases without undue further delay.

(86) The controller should communicate to the data subject a personal data breach, without undue delay, where that personal data breach is likely to result in a high risk to the rights and freedoms of the natural person in order to allow him or her to take the necessary precautions. The communication should describe the nature of the personal data breach as well as recommendations for the natural person concerned to mitigate potential adverse effects. Such communications to data subjects should be made as soon as reasonably feasible and in close cooperation with the supervisory authority, respecting guidance provided by it or by other relevant authorities such as law-enforcement authorities. For example, the need to mitigate an immediate risk of damage would call for prompt communication with data subjects whereas the need to implement appropriate measures against continuing or similar personal data breaches may justify more time for communication.

(87) It should be ascertained whether all appropriate technological protection and organisational measures have been implemented to establish immediately whether a personal data breach has taken place and to inform promptly the supervisory authority and the data subject. The fact that the notification was made without undue delay should be established taking into account in particular the nature and gravity of the personal data breach and its consequences and adverse effects for the data subject. Such notification may result in an intervention of the supervisory authority in accordance with its tasks and powers laid down in this Regulation.

(88) In setting detailed rules concerning the format and procedures applicable to the notification of personal data breaches, due consideration should be given to the circumstances of that breach, including whether or not personal data had been protected by appropriate technical protection measures, effectively limiting the likelihood of identity fraud or other forms of misuse. Moreover, such rules and procedures should take into account the legitimate interests of law-enforcement authorities where early disclosure could unnecessarily hamper the investigation of the circumstances of a personal data breach.

(89) Directive 95/46/EC provided for a general obligation to notify the processing of personal data to the supervisory authorities. While that obligation produces administrative and financial burdens, it did not in all cases contribute to improving the protection of personal data. Such indiscriminate general notification obligations should therefore be abolished, and replaced by effective procedures and mechanisms which focus instead on those types of processing operations which are likely to result in a high risk to the rights and freedoms of natural persons by virtue of their nature, scope, context and purposes. Such types of processing operations may be those which in, particular, involve using new technologies, or are of a new kind and where no data protection impact assessment has been carried out before by the controller, or where they become necessary in the light of the time that has elapsed since the initial processing.

(90) In such cases, a data protection impact assessment should be carried out by the controller prior to the processing in order to assess the particular likelihood and severity of the high risk, taking into account the nature, scope, context and purposes of the processing and the sources of the risk. That impact assessment should include, in particular, the measures, safeguards and mechanisms envisaged for mitigating that risk, ensuring the protection of personal data and demonstrating compliance with this Regulation.

(91) This should in particular apply to large-scale processing operations which aim to process a considerable amount of personal data at regional, national or supranational level and which could affect a large number of data subjects and which are likely to result in a high risk, for example, on account of their sensitivity, where in accordance with the achieved state of technological knowledge a new technology is used on a large scale as well as to other processing operations which result in a high risk to the rights and freedoms of data subjects, in particular where those operations render it more difficult for data subjects to exercise their rights. A data

protection impact assessment should also be made where personal data are processed for taking decisions regarding specific natural persons following any systematic and extensive evaluation of personal aspects relating to natural persons based on profiling those data or following the processing of special categories of personal data, biometric data, or data on criminal convictions and offences or related security measures. A data protection impact assessment is equally required for monitoring publicly accessible areas on a large scale, especially when using optic-electronic devices or for any other operations where the competent supervisory authority considers that the processing is likely to result in a high risk to the rights and freedoms of data subjects, in particular because they prevent data subjects from exercising a right or using a service or a contract, or because they are carried out systematically on a large scale. The processing of personal data should not be considered to be on a large scale if the processing concerns personal data from patients or clients by an individual physician, other health care professional or lawyer. In such cases, a data protection impact assessment should not be mandatory.

(92) There are circumstances under which it may be reasonable and economical for the subject of a data protection impact assessment to be broader than a single project, for example where public authorities or bodies intend to establish a common application or processing platform or where several controllers plan to introduce a common application or processing environment across an industry sector or segment or for a widely used horizontal activity.

(93) In the context of the adoption of the Member State law on which the performance of the tasks of the public authority or public body is based and which regulates the specific processing operation or set of operations in question, Member States may deem it necessary to carry out such assessment prior to the processing activities.

(94) Where a data protection impact assessment indicates that the processing would, in the absence of safeguards, security measures and mechanisms to mitigate the risk, result in a high risk to the rights and freedoms of natural persons and the controller is of the opinion that the risk cannot be mitigated by reasonable means in terms of available technologies and costs of implementation, the supervisory authority should be consulted prior to the start of processing activities. Such high risk is likely to result from certain types of processing and the extent and frequency of processing, which may result also in a realisation of damage or interference with the rights and freedoms of the natural person. The supervisory authority should respond to the request for consultation within a specified period. However, the absence of a reaction of the supervisory authority within that period should be without prejudice to any intervention of the supervisory authority in accordance with its tasks and powers laid down in this Regulation, including the power to prohibit processing operations. As part of that consultation process, the outcome of a data protection impact assessment carried out with regard to the processing at issue may be submitted to the supervisory authority, in particular the measures envisaged to mitigate the risk to the rights and freedoms of natural persons.

(95) The processor should assist the controller, where necessary and upon request, in ensuring compliance with the obligations deriving from the carrying out of data protection impact assessments and from prior consultation of the supervisory authority.

(96) A consultation of the supervisory authority should also take place in the course of the preparation of a legislative or regulatory measure which provides for the processing of personal data, in order to ensure compliance of the intended processing with this Regulation and in particular to mitigate the risk involved for the data subject.

(97) Where the processing is carried out by a public authority, except for courts or independent judicial authorities when acting in their judicial capacity, where, in the private sector, processing is carried out by a controller whose core activities consist of processing operations that require regular and systematic monitoring of the data subjects on a large scale, or where the core activities of the controller or the processor consist of processing on a large scale of special categories of personal data and data relating to criminal convictions and offences, a person with expert knowledge of data protection law and practices should assist the controller or processor to monitor internal compliance with this Regulation. In the private sector, the core activities of a controller relate to its primary activities and do not relate to the processing of personal data as ancillary activities. The necessary level of expert knowledge should be determined in particular according to the data processing operations carried out

and the protection required for the personal data processed by the controller or the processor. Such data protection officers, whether or not they are an employee of the controller, should be in a position to perform their duties and tasks in an independent manner.

(98) Associations or other bodies representing categories of controllers or processors should be encouraged to draw up codes of conduct, within the limits of this Regulation, so as to facilitate the effective application of this Regulation, taking account of the specific characteristics of the processing carried out in certain sectors and the specific needs of micro, small and medium enterprises. In particular, such codes of conduct could calibrate the obligations of controllers and processors, taking into account the risk likely to result from the processing for the rights and freedoms of natural persons.

(99) When drawing up a code of conduct, or when amending or extending such a code, associations and other bodies representing categories of controllers or processors should consult relevant stakeholders, including data subjects where feasible, and have regard to submissions received and views expressed in response to such consultations.

(100) In order to enhance transparency and compliance with this Regulation, the establishment of certification mechanisms and data protection seals and marks should be encouraged, allowing data subjects to quickly assess the level of data protection of relevant products and services.

(101) Flows of personal data to and from countries outside the Union and international organisations are necessary for the expansion of international trade and international cooperation. The increase in such flows has raised new challenges and concerns with regard to the protection of personal data. However, when personal data are transferred from the Union to controllers, processors or other recipients in third countries or to international organisations, the level of protection of natural persons ensured in the Union by this Regulation should not be undermined, including in cases of onward transfers of personal data from the third country or international organisation to controllers, processors in the same or another third country or international organisation. In any event, transfers to third countries and international organisations may only be carried out in full compliance with this Regulation. A transfer could take place only if, subject to the other provisions of this Regulation, the conditions laid down in the provisions of this Regulation relating to the transfer of personal data to third countries or international organisations are complied with by the controller or processor.

(102) This Regulation is without prejudice to international agreements concluded between the Union and third countries regulating the transfer of personal data including appropriate safeguards for the data subjects. Member States may conclude international agreements which involve the transfer of personal data to third countries or international organisations, as far as such agreements do not affect this Regulation or any other provisions of Union law and include an appropriate level of protection for the fundamental rights of the data subjects.

(103) The Commission may decide with effect for the entire Union that a third country, a territory or specified sector within a third country, or an international organisation, offers an adequate level of data protection, thus providing legal certainty and uniformity throughout the Union as regards the third country or international organisation which is considered to provide such level of protection. In such cases, transfers of personal data to that third country or international organisation may take place without the need to obtain any further authorisation. The Commission may also decide, having given notice and a full statement setting out the reasons to the third country or international organisation, to revoke such a decision.

(104) In line with the fundamental values on which the Union is founded, in particular the protection of human rights, the Commission should, in its assessment of the third country, or of a territory or specified sector within a third country, take into account how a particular third country respects the rule of law, access to justice as well as international human rights norms and standards and its general and sectoral law, including legislation concerning public security, defence and national security as well as public order and criminal law. The adoption of an adequacy decision with regard to a territory or a specified sector in a third country should take into account clear and objective criteria, such as specific processing activities and the scope of applicable legal standards and legislation in force in the third country. The third country should offer guarantees ensuring an adequate level of

protection essentially equivalent to that ensured within the Union, in particular where personal data are processed in one or several specific sectors. In particular, the third country should ensure effective independent data protection supervision and should provide for cooperation mechanisms with the Member States' data protection authorities, and the data subjects should be provided with effective and enforceable rights and effective administrative and judicial redress.

(105) Apart from the international commitments the third country or international organisation has entered into, the Commission should take account of obligations arising from the third country's or international organisation's participation in multilateral or regional systems in particular in relation to the protection of personal data, as well as the implementation of such obligations. In particular, the third country's accession to the Council of Europe Convention of 28 January 1981 for the Protection of Individuals with regard to the Automatic Processing of Personal Data and its Additional Protocol should be taken into account. The Commission should consult the Board when assessing the level of protection in third countries or international organisations.

(106) The Commission should monitor the functioning of decisions on the level of protection in a third country, a territory or specified sector within a third country, or an international organisation, and monitor the functioning of decisions adopted on the basis of Article 25(6) or Article 26(4) of Directive 95/46/EC. In its adequacy decisions, the Commission should provide for a periodic review mechanism of their functioning. That periodic review should be conducted in consultation with the third country or international organisation in question and take into account all relevant developments in the third country or international organisation. For the purposes of monitoring and of carrying out the periodic reviews, the Commission should take into consideration the views and findings of the European Parliament and of the Council as well as of other relevant bodies and sources. The Commission should evaluate, within a reasonable time, the functioning of the latter decisions and report any relevant findings to the Committee within the meaning of Regulation (EU) No 182/2011 of the European Parliament and of the Council ([1]) as established under this Regulation, to the European Parliament and to the Council.

(107) The Commission may recognise that a third country, a territory or a specified sector within a third country, or an international organisation no longer ensures an adequate level of data protection. Consequently the transfer of personal data to that third country or international organisation should be prohibited, unless the requirements in this Regulation relating to transfers subject to appropriate safeguards, including binding corporate rules, and derogations for specific situations are fulfilled. In that case, provision should be made for consultations between the Commission and such third countries or international organisations. The Commission should, in a timely manner, inform the third country or international organisation of the reasons and enter into consultations with it in order to remedy the situation.

(108) In the absence of an adequacy decision, the controller or processor should take measures to compensate for the lack of data protection in a third country by way of appropriate safeguards for the data subject. Such appropriate safeguards may consist of making use of binding corporate rules, standard data protection clauses adopted by the Commission, standard data protection clauses adopted by a supervisory authority or contractual clauses authorised by a supervisory authority. Those safeguards should ensure compliance with data protection requirements and the rights of the data subjects appropriate to processing within the Union, including the availability of enforceable data subject rights and of effective legal remedies, including to obtain effective administrative or judicial redress and to claim compensation, in the Union or in a third country. They should relate in particular to compliance with the general principles relating to personal data processing, the principles of data protection by design and by default. Transfers may also be carried out by public authorities or bodies with public authorities or bodies in third countries or with international organisations with corresponding duties or functions, including on the basis of provisions to be inserted into administrative arrangements, such as a memorandum of understanding, providing for enforceable and effective rights for data subjects. Authorisation by the competent supervisory authority should be obtained when the safeguards are provided for in administrative arrangements that are not legally binding.

(109) The possibility for the controller or processor to use standard data-protection clauses adopted by the Commission or by a supervisory authority should prevent controllers or processors neither from including the

([1]) Regulation (EU) No 182/2011 of the European Parliament and of the Council of 16 February 2011 laying down the rules and general principles concerning mechanisms for control by Member States of the Commission's exercise of implementing powers (OJ L 55, 28.2.2011, p. 13).

standard data-protection clauses in a wider contract, such as a contract between the processor and another processor, nor from adding other clauses or additional safeguards provided that they do not contradict, directly or indirectly, the standard contractual clauses adopted by the Commission or by a supervisory authority or prejudice the fundamental rights or freedoms of the data subjects. Controllers and processors should be encouraged to provide additional safeguards via contractual commitments that supplement standard protection clauses.

(110) A group of undertakings, or a group of enterprises engaged in a joint economic activity, should be able to make use of approved binding corporate rules for its international transfers from the Union to organisations within the same group of undertakings, or group of enterprises engaged in a joint economic activity, provided that such corporate rules include all essential principles and enforceable rights to ensure appropriate safeguards for transfers or categories of transfers of personal data.

(111) Provisions should be made for the possibility for transfers in certain circumstances where the data subject has given his or her explicit consent, where the transfer is occasional and necessary in relation to a contract or a legal claim, regardless of whether in a judicial procedure or whether in an administrative or any out-of-court procedure, including procedures before regulatory bodies. Provision should also be made for the possibility for transfers where important grounds of public interest laid down by Union or Member State law so require or where the transfer is made from a register established by law and intended for consultation by the public or persons having a legitimate interest. In the latter case, such a transfer should not involve the entirety of the personal data or entire categories of the data contained in the register and, when the register is intended for consultation by persons having a legitimate interest, the transfer should be made only at the request of those persons or, if they are to be the recipients, taking into full account the interests and fundamental rights of the data subject.

(112) Those derogations should in particular apply to data transfers required and necessary for important reasons of public interest, for example in cases of international data exchange between competition authorities, tax or customs administrations, between financial supervisory authorities, between services competent for social security matters, or for public health, for example in the case of contact tracing for contagious diseases or in order to reduce and/or eliminate doping in sport. A transfer of personal data should also be regarded as lawful where it is necessary to protect an interest which is essential for the data subject's or another person's vital interests, including physical integrity or life, if the data subject is incapable of giving consent. In the absence of an adequacy decision, Union or Member State law may, for important reasons of public interest, expressly set limits to the transfer of specific categories of data to a third country or an international organisation. Member States should notify such provisions to the Commission. Any transfer to an international humanitarian organisation of personal data of a data subject who is physically or legally incapable of giving consent, with a view to accomplishing a task incumbent under the Geneva Conventions or to complying with international humanitarian law applicable in armed conflicts, could be considered to be necessary for an important reason of public interest or because it is in the vital interest of the data subject.

(113) Transfers which can be qualified as not repetitive and that only concern a limited number of data subjects, could also be possible for the purposes of the compelling legitimate interests pursued by the controller, when those interests are not overridden by the interests or rights and freedoms of the data subject and when the controller has assessed all the circumstances surrounding the data transfer. The controller should give particular consideration to the nature of the personal data, the purpose and duration of the proposed processing operation or operations, as well as the situation in the country of origin, the third country and the country of final destination, and should provide suitable safeguards to protect fundamental rights and freedoms of natural persons with regard to the processing of their personal data. Such transfers should be possible only in residual cases where none of the other grounds for transfer are applicable. For scientific or historical research purposes or statistical purposes, the legitimate expectations of society for an increase of knowledge should be taken into consideration. The controller should inform the supervisory authority and the data subject about the transfer.

(114) In any case, where the Commission has taken no decision on the adequate level of data protection in a third country, the controller or processor should make use of solutions that provide data subjects with enforceable and effective rights as regards the processing of their data in the Union once those data have been transferred so that that they will continue to benefit from fundamental rights and safeguards.

(115) Some third countries adopt laws, regulations and other legal acts which purport to directly regulate the processing activities of natural and legal persons under the jurisdiction of the Member States. This may include judgments of courts or tribunals or decisions of administrative authorities in third countries requiring a controller or processor to transfer or disclose personal data, and which are not based on an international agreement, such as a mutual legal assistance treaty, in force between the requesting third country and the Union or a Member State. The extraterritorial application of those laws, regulations and other legal acts may be in breach of international law and may impede the attainment of the protection of natural persons ensured in the Union by this Regulation. Transfers should only be allowed where the conditions of this Regulation for a transfer to third countries are met. This may be the case, inter alia, where disclosure is necessary for an important ground of public interest recognised in Union or Member State law to which the controller is subject.

(116) When personal data moves across borders outside the Union it may put at increased risk the ability of natural persons to exercise data protection rights in particular to protect themselves from the unlawful use or disclosure of that information. At the same time, supervisory authorities may find that they are unable to pursue complaints or conduct investigations relating to the activities outside their borders. Their efforts to work together in the cross-border context may also be hampered by insufficient preventative or remedial powers, inconsistent legal regimes, and practical obstacles like resource constraints. Therefore, there is a need to promote closer cooperation among data protection supervisory authorities to help them exchange information and carry out investigations with their international counterparts. For the purposes of developing international cooperation mechanisms to facilitate and provide international mutual assistance for the enforcement of legislation for the protection of personal data, the Commission and the supervisory authorities should exchange information and cooperate in activities related to the exercise of their powers with competent authorities in third countries, based on reciprocity and in accordance with this Regulation.

(117) The establishment of supervisory authorities in Member States, empowered to perform their tasks and exercise their powers with complete independence, is an essential component of the protection of natural persons with regard to the processing of their personal data. Member States should be able to establish more than one supervisory authority, to reflect their constitutional, organisational and administrative structure.

(118) The independence of supervisory authorities should not mean that the supervisory authorities cannot be subject to control or monitoring mechanisms regarding their financial expenditure or to judicial review.

(119) Where a Member State establishes several supervisory authorities, it should establish by law mechanisms for ensuring the effective participation of those supervisory authorities in the consistency mechanism. That Member State should in particular designate the supervisory authority which functions as a single contact point for the effective participation of those authorities in the mechanism, to ensure swift and smooth cooperation with other supervisory authorities, the Board and the Commission.

(120) Each supervisory authority should be provided with the financial and human resources, premises and infrastructure necessary for the effective performance of their tasks, including those related to mutual assistance and cooperation with other supervisory authorities throughout the Union. Each supervisory authority should have a separate, public annual budget, which may be part of the overall state or national budget.

(121) The general conditions for the member or members of the supervisory authority should be laid down by law in each Member State and should in particular provide that those members are to be appointed, by means of a transparent procedure, either by the parliament, government or the head of State of the Member State on the basis of a proposal from the government, a member of the government, the parliament or a chamber of the parliament, or by an independent body entrusted under Member State law. In order to ensure the independence of the supervisory authority, the member or members should act with integrity, refrain from any action that is incompatible with their duties and should not, during their term of office, engage in any incompatible occupation, whether gainful or not. The supervisory authority should have its own staff, chosen by the supervisory authority or an independent body established by Member State law, which should be subject to the exclusive direction of the member or members of the supervisory authority.

(122) Each supervisory authority should be competent on the territory of its own Member State to exercise the powers and to perform the tasks conferred on it in accordance with this Regulation. This should cover in particular the

processing in the context of the activities of an establishment of the controller or processor on the territory of its own Member State, the processing of personal data carried out by public authorities or private bodies acting in the public interest, processing affecting data subjects on its territory or processing carried out by a controller or processor not established in the Union when targeting data subjects residing on its territory. This should include handling complaints lodged by a data subject, conducting investigations on the application of this Regulation and promoting public awareness of the risks, rules, safeguards and rights in relation to the processing of personal data.

(123) The supervisory authorities should monitor the application of the provisions pursuant to this Regulation and contribute to its consistent application throughout the Union, in order to protect natural persons in relation to the processing of their personal data and to facilitate the free flow of personal data within the internal market. For that purpose, the supervisory authorities should cooperate with each other and with the Commission, without the need for any agreement between Member States on the provision of mutual assistance or on such cooperation.

(124) Where the processing of personal data takes place in the context of the activities of an establishment of a controller or a processor in the Union and the controller or processor is established in more than one Member State, or where processing taking place in the context of the activities of a single establishment of a controller or processor in the Union substantially affects or is likely to substantially affect data subjects in more than one Member State, the supervisory authority for the main establishment of the controller or processor or for the single establishment of the controller or processor should act as lead authority. It should cooperate with the other authorities concerned, because the controller or processor has an establishment on the territory of their Member State, because data subjects residing on their territory are substantially affected, or because a complaint has been lodged with them. Also where a data subject not residing in that Member State has lodged a complaint, the supervisory authority with which such complaint has been lodged should also be a supervisory authority concerned. Within its tasks to issue guidelines on any question covering the application of this Regulation, the Board should be able to issue guidelines in particular on the criteria to be taken into account in order to ascertain whether the processing in question substantially affects data subjects in more than one Member State and on what constitutes a relevant and reasoned objection.

(125) The lead authority should be competent to adopt binding decisions regarding measures applying the powers conferred on it in accordance with this Regulation. In its capacity as lead authority, the supervisory authority should closely involve and coordinate the supervisory authorities concerned in the decision-making process. Where the decision is to reject the complaint by the data subject in whole or in part, that decision should be adopted by the supervisory authority with which the complaint has been lodged.

(126) The decision should be agreed jointly by the lead supervisory authority and the supervisory authorities concerned and should be directed towards the main or single establishment of the controller or processor and be binding on the controller and processor. The controller or processor should take the necessary measures to ensure compliance with this Regulation and the implementation of the decision notified by the lead supervisory authority to the main establishment of the controller or processor as regards the processing activities in the Union.

(127) Each supervisory authority not acting as the lead supervisory authority should be competent to handle local cases where the controller or processor is established in more than one Member State, but the subject matter of the specific processing concerns only processing carried out in a single Member State and involves only data subjects in that single Member State, for example, where the subject matter concerns the processing of employees' personal data in the specific employment context of a Member State. In such cases, the supervisory authority should inform the lead supervisory authority without delay about the matter. After being informed, the lead supervisory authority should decide, whether it will handle the case pursuant to the provision on cooperation between the lead supervisory authority and other supervisory authorities concerned ('one-stop-shop mechanism'), or whether the supervisory authority which informed it should handle the case at local level. When deciding whether it will handle the case, the lead supervisory authority should take into account whether there is an establishment of the controller or processor in the Member State of the supervisory authority which informed it in order to ensure effective enforcement of a decision *vis-à-vis* the controller or processor. Where the lead supervisory authority decides to handle the case, the supervisory authority which informed it should have the

possibility to submit a draft for a decision, of which the lead supervisory authority should take utmost account when preparing its draft decision in that one-stop-shop mechanism.

(128) The rules on the lead supervisory authority and the one-stop-shop mechanism should not apply where the processing is carried out by public authorities or private bodies in the public interest. In such cases the only supervisory authority competent to exercise the powers conferred to it in accordance with this Regulation should be the supervisory authority of the Member State where the public authority or private body is established.

(129) In order to ensure consistent monitoring and enforcement of this Regulation throughout the Union, the supervisory authorities should have in each Member State the same tasks and effective powers, including powers of investigation, corrective powers and sanctions, and authorisation and advisory powers, in particular in cases of complaints from natural persons, and without prejudice to the powers of prosecutorial authorities under Member State law, to bring infringements of this Regulation to the attention of the judicial authorities and engage in legal proceedings. Such powers should also include the power to impose a temporary or definitive limitation, including a ban, on processing. Member States may specify other tasks related to the protection of personal data under this Regulation. The powers of supervisory authorities should be exercised in accordance with appropriate procedural safeguards set out in Union and Member State law, impartially, fairly and within a reasonable time. In particular each measure should be appropriate, necessary and proportionate in view of ensuring compliance with this Regulation, taking into account the circumstances of each individual case, respect the right of every person to be heard before any individual measure which would affect him or her adversely is taken and avoid superfluous costs and excessive inconveniences for the persons concerned. Investigatory powers as regards access to premises should be exercised in accordance with specific requirements in Member State procedural law, such as the requirement to obtain a prior judicial authorisation. Each legally binding measure of the supervisory authority should be in writing, be clear and unambiguous, indicate the supervisory authority which has issued the measure, the date of issue of the measure, bear the signature of the head, or a member of the supervisory authority authorised by him or her, give the reasons for the measure, and refer to the right of an effective remedy. This should not preclude additional requirements pursuant to Member State procedural law. The adoption of a legally binding decision implies that it may give rise to judicial review in the Member State of the supervisory authority that adopted the decision.

(130) Where the supervisory authority with which the complaint has been lodged is not the lead supervisory authority, the lead supervisory authority should closely cooperate with the supervisory authority with which the complaint has been lodged in accordance with the provisions on cooperation and consistency laid down in this Regulation. In such cases, the lead supervisory authority should, when taking measures intended to produce legal effects, including the imposition of administrative fines, take utmost account of the view of the supervisory authority with which the complaint has been lodged and which should remain competent to carry out any investigation on the territory of its own Member State in liaison with the competent supervisory authority.

(131) Where another supervisory authority should act as a lead supervisory authority for the processing activities of the controller or processor but the concrete subject matter of a complaint or the possible infringement concerns only processing activities of the controller or processor in the Member State where the complaint has been lodged or the possible infringement detected and the matter does not substantially affect or is not likely to substantially affect data subjects in other Member States, the supervisory authority receiving a complaint or detecting or being informed otherwise of situations that entail possible infringements of this Regulation should seek an amicable settlement with the controller and, if this proves unsuccessful, exercise its full range of powers. This should include: specific processing carried out in the territory of the Member State of the supervisory authority or with regard to data subjects on the territory of that Member State; processing that is carried out in the context of an offer of goods or services specifically aimed at data subjects in the territory of the Member State of the supervisory authority; or processing that has to be assessed taking into account relevant legal obligations under Member State law.

(132) Awareness-raising activities by supervisory authorities addressed to the public should include specific measures directed at controllers and processors, including micro, small and medium-sized enterprises, as well as natural persons in particular in the educational context.

(133) The supervisory authorities should assist each other in performing their tasks and provide mutual assistance, so as to ensure the consistent application and enforcement of this Regulation in the internal market. A supervisory authority requesting mutual assistance may adopt a provisional measure if it receives no response to a request for mutual assistance within one month of the receipt of that request by the other supervisory authority.

(134) Each supervisory authority should, where appropriate, participate in joint operations with other supervisory authorities. The requested supervisory authority should be obliged to respond to the request within a specified time period.

(135) In order to ensure the consistent application of this Regulation throughout the Union, a consistency mechanism for cooperation between the supervisory authorities should be established. That mechanism should in particular apply where a supervisory authority intends to adopt a measure intended to produce legal effects as regards processing operations which substantially affect a significant number of data subjects in several Member States. It should also apply where any supervisory authority concerned or the Commission requests that such matter should be handled in the consistency mechanism. That mechanism should be without prejudice to any measures that the Commission may take in the exercise of its powers under the Treaties.

(136) In applying the consistency mechanism, the Board should, within a determined period of time, issue an opinion, if a majority of its members so decides or if so requested by any supervisory authority concerned or the Commission. The Board should also be empowered to adopt legally binding decisions where there are disputes between supervisory authorities. For that purpose, it should issue, in principle by a two-thirds majority of its members, legally binding decisions in clearly specified cases where there are conflicting views among supervisory authorities, in particular in the cooperation mechanism between the lead supervisory authority and supervisory authorities concerned on the merits of the case, in particular whether there is an infringement of this Regulation.

(137) There may be an urgent need to act in order to protect the rights and freedoms of data subjects, in particular when the danger exists that the enforcement of a right of a data subject could be considerably impeded. A supervisory authority should therefore be able to adopt duly justified provisional measures on its territory with a specified period of validity which should not exceed three months.

(138) The application of such mechanism should be a condition for the lawfulness of a measure intended to produce legal effects by a supervisory authority in those cases where its application is mandatory. In other cases of cross-border relevance, the cooperation mechanism between the lead supervisory authority and supervisory authorities concerned should be applied and mutual assistance and joint operations might be carried out between the supervisory authorities concerned on a bilateral or multilateral basis without triggering the consistency mechanism.

(139) In order to promote the consistent application of this Regulation, the Board should be set up as an independent body of the Union. To fulfil its objectives, the Board should have legal personality. The Board should be represented by its Chair. It should replace the Working Party on the Protection of Individuals with Regard to the Processing of Personal Data established by Directive 95/46/EC. It should consist of the head of a supervisory authority of each Member State and the European Data Protection Supervisor or their respective representatives. The Commission should participate in the Board's activities without voting rights and the European Data Protection Supervisor should have specific voting rights. The Board should contribute to the consistent application of this Regulation throughout the Union, including by advising the Commission, in particular on the level of protection in third countries or international organisations, and promoting cooperation of the supervisory authorities throughout the Union. The Board should act independently when performing its tasks.

(140) The Board should be assisted by a secretariat provided by the European Data Protection Supervisor. The staff of the European Data Protection Supervisor involved in carrying out the tasks conferred on the Board by this Regulation should perform its tasks exclusively under the instructions of, and report to, the Chair of the Board.

(141) Every data subject should have the right to lodge a complaint with a single supervisory authority, in particular in the Member State of his or her habitual residence, and the right to an effective judicial remedy in accordance

with Article 47 of the Charter if the data subject considers that his or her rights under this Regulation are infringed or where the supervisory authority does not act on a complaint, partially or wholly rejects or dismisses a complaint or does not act where such action is necessary to protect the rights of the data subject. The investigation following a complaint should be carried out, subject to judicial review, to the extent that is appropriate in the specific case. The supervisory authority should inform the data subject of the progress and the outcome of the complaint within a reasonable period. If the case requires further investigation or coordination with another supervisory authority, intermediate information should be given to the data subject. In order to facilitate the submission of complaints, each supervisory authority should take measures such as providing a complaint submission form which can also be completed electronically, without excluding other means of communication.

(142) Where a data subject considers that his or her rights under this Regulation are infringed, he or she should have the right to mandate a not-for-profit body, organisation or association which is constituted in accordance with the law of a Member State, has statutory objectives which are in the public interest and is active in the field of the protection of personal data to lodge a complaint on his or her behalf with a supervisory authority, exercise the right to a judicial remedy on behalf of data subjects or, if provided for in Member State law, exercise the right to receive compensation on behalf of data subjects. A Member State may provide for such a body, organisation or association to have the right to lodge a complaint in that Member State, independently of a data subject's mandate, and the right to an effective judicial remedy where it has reasons to consider that the rights of a data subject have been infringed as a result of the processing of personal data which infringes this Regulation. That body, organisation or association may not be allowed to claim compensation on a data subject's behalf independently of the data subject's mandate.

(143) Any natural or legal person has the right to bring an action for annulment of decisions of the Board before the Court of Justice under the conditions provided for in Article 263 TFEU. As addressees of such decisions, the supervisory authorities concerned which wish to challenge them have to bring action within two months of being notified of them, in accordance with Article 263 TFEU. Where decisions of the Board are of direct and individual concern to a controller, processor or complainant, the latter may bring an action for annulment against those decisions within two months of their publication on the website of the Board, in accordance with Article 263 TFEU. Without prejudice to this right under Article 263 TFEU, each natural or legal person should have an effective judicial remedy before the competent national court against a decision of a supervisory authority which produces legal effects concerning that person. Such a decision concerns in particular the exercise of investigative, corrective and authorisation powers by the supervisory authority or the dismissal or rejection of complaints. However, the right to an effective judicial remedy does not encompass measures taken by supervisory authorities which are not legally binding, such as opinions issued by or advice provided by the supervisory authority. Proceedings against a supervisory authority should be brought before the courts of the Member State where the supervisory authority is established and should be conducted in accordance with that Member State's procedural law. Those courts should exercise full jurisdiction, which should include jurisdiction to examine all questions of fact and law relevant to the dispute before them.

Where a complaint has been rejected or dismissed by a supervisory authority, the complainant may bring proceedings before the courts in the same Member State. In the context of judicial remedies relating to the application of this Regulation, national courts which consider a decision on the question necessary to enable them to give judgment, may, or in the case provided for in Article 267 TFEU, must, request the Court of Justice to give a preliminary ruling on the interpretation of Union law, including this Regulation. Furthermore, where a decision of a supervisory authority implementing a decision of the Board is challenged before a national court and the validity of the decision of the Board is at issue, that national court does not have the power to declare the Board's decision invalid but must refer the question of validity to the Court of Justice in accordance with Article 267 TFEU as interpreted by the Court of Justice, where it considers the decision invalid. However, a national court may not refer a question on the validity of the decision of the Board at the request of a natural or legal person which had the opportunity to bring an action for annulment of that decision, in particular if it was directly and individually concerned by that decision, but had not done so within the period laid down in Article 263 TFEU.

(144) Where a court seized of proceedings against a decision by a supervisory authority has reason to believe that proceedings concerning the same processing, such as the same subject matter as regards processing by the same controller or processor, or the same cause of action, are brought before a competent court in another Member State, it should contact that court in order to confirm the existence of such related proceedings. If related proceedings are pending before a court in another Member State, any court other than the court first

seized may stay its proceedings or may, on request of one of the parties, decline jurisdiction in favour of the court first seized if that court has jurisdiction over the proceedings in question and its law permits the consolidation of such related proceedings. Proceedings are deemed to be related where they are so closely connected that it is expedient to hear and determine them together in order to avoid the risk of irreconcilable judgments resulting from separate proceedings.

(145) For proceedings against a controller or processor, the plaintiff should have the choice to bring the action before the courts of the Member States where the controller or processor has an establishment or where the data subject resides, unless the controller is a public authority of a Member State acting in the exercise of its public powers.

(146) The controller or processor should compensate any damage which a person may suffer as a result of processing that infringes this Regulation. The controller or processor should be exempt from liability if it proves that it is not in any way responsible for the damage. The concept of damage should be broadly interpreted in the light of the case-law of the Court of Justice in a manner which fully reflects the objectives of this Regulation. This is without prejudice to any claims for damage deriving from the violation of other rules in Union or Member State law. Processing that infringes this Regulation also includes processing that infringes delegated and implementing acts adopted in accordance with this Regulation and Member State law specifying rules of this Regulation. Data subjects should receive full and effective compensation for the damage they have suffered. Where controllers or processors are involved in the same processing, each controller or processor should be held liable for the entire damage. However, where they are joined to the same judicial proceedings, in accordance with Member State law, compensation may be apportioned according to the responsibility of each controller or processor for the damage caused by the processing, provided that full and effective compensation of the data subject who suffered the damage is ensured. Any controller or processor which has paid full compensation may subsequently institute recourse proceedings against other controllers or processors involved in the same processing.

(147) Where specific rules on jurisdiction are contained in this Regulation, in particular as regards proceedings seeking a judicial remedy including compensation, against a controller or processor, general jurisdiction rules such as those of Regulation (EU) No 1215/2012 of the European Parliament and of the Council (1) should not prejudice the application of such specific rules.

(148) In order to strengthen the enforcement of the rules of this Regulation, penalties including administrative fines should be imposed for any infringement of this Regulation, in addition to, or instead of appropriate measures imposed by the supervisory authority pursuant to this Regulation. In a case of a minor infringement or if the fine likely to be imposed would constitute a disproportionate burden to a natural person, a reprimand may be issued instead of a fine. Due regard should however be given to the nature, gravity and duration of the infringement, the intentional character of the infringement, actions taken to mitigate the damage suffered, degree of responsibility or any relevant previous infringements, the manner in which the infringement became known to the supervisory authority, compliance with measures ordered against the controller or processor, adherence to a code of conduct and any other aggravating or mitigating factor. The imposition of penalties including administrative fines should be subject to appropriate procedural safeguards in accordance with the general principles of Union law and the Charter, including effective judicial protection and due process.

(149) Member States should be able to lay down the rules on criminal penalties for infringements of this Regulation, including for infringements of national rules adopted pursuant to and within the limits of this Regulation. Those criminal penalties may also allow for the deprivation of the profits obtained through infringements of this Regulation. However, the imposition of criminal penalties for infringements of such national rules and of administrative penalties should not lead to a breach of the principle of *ne bis in idem*, as interpreted by the Court of Justice.

(150) In order to strengthen and harmonise administrative penalties for infringements of this Regulation, each supervisory authority should have the power to impose administrative fines. This Regulation should indicate

(1) Regulation (EU) No 1215/2012 of the European Parliament and of the Council of 12 December 2012 on jurisdiction and the recognition and enforcement of judgments in civil and commercial matters (OJ L 351, 20.12.2012, p. 1).

infringements and the upper limit and criteria for setting the related administrative fines, which should be determined by the competent supervisory authority in each individual case, taking into account all relevant circumstances of the specific situation, with due regard in particular to the nature, gravity and duration of the infringement and of its consequences and the measures taken to ensure compliance with the obligations under this Regulation and to prevent or mitigate the consequences of the infringement. Where administrative fines are imposed on an undertaking, an undertaking should be understood to be an undertaking in accordance with Articles 101 and 102 TFEU for those purposes. Where administrative fines are imposed on persons that are not an undertaking, the supervisory authority should take account of the general level of income in the Member State as well as the economic situation of the person in considering the appropriate amount of the fine. The consistency mechanism may also be used to promote a consistent application of administrative fines. It should be for the Member States to determine whether and to which extent public authorities should be subject to administrative fines. Imposing an administrative fine or giving a warning does not affect the application of other powers of the supervisory authorities or of other penalties under this Regulation.

(151) The legal systems of Denmark and Estonia do not allow for administrative fines as set out in this Regulation. The rules on administrative fines may be applied in such a manner that in Denmark the fine is imposed by competent national courts as a criminal penalty and in Estonia the fine is imposed by the supervisory authority in the framework of a misdemeanour procedure, provided that such an application of the rules in those Member States has an equivalent effect to administrative fines imposed by supervisory authorities. Therefore the competent national courts should take into account the recommendation by the supervisory authority initiating the fine. In any event, the fines imposed should be effective, proportionate and dissuasive.

(152) Where this Regulation does not harmonise administrative penalties or where necessary in other cases, for example in cases of serious infringements of this Regulation, Member States should implement a system which provides for effective, proportionate and dissuasive penalties. The nature of such penalties, criminal or administrative, should be determined by Member State law.

(153) Member States law should reconcile the rules governing freedom of expression and information, including journalistic, academic, artistic and or literary expression with the right to the protection of personal data pursuant to this Regulation. The processing of personal data solely for journalistic purposes, or for the purposes of academic, artistic or literary expression should be subject to derogations or exemptions from certain provisions of this Regulation if necessary to reconcile the right to the protection of personal data with the right to freedom of expression and information, as enshrined in Article 11 of the Charter. This should apply in particular to the processing of personal data in the audiovisual field and in news archives and press libraries. Therefore, Member States should adopt legislative measures which lay down the exemptions and derogations necessary for the purpose of balancing those fundamental rights. Member States should adopt such exemptions and derogations on general principles, the rights of the data subject, the controller and the processor, the transfer of personal data to third countries or international organisations, the independent supervisory authorities, cooperation and consistency, and specific data-processing situations. Where such exemptions or derogations differ from one Member State to another, the law of the Member State to which the controller is subject should apply. In order to take account of the importance of the right to freedom of expression in every democratic society, it is necessary to interpret notions relating to that freedom, such as journalism, broadly.

(154) This Regulation allows the principle of public access to official documents to be taken into account when applying this Regulation. Public access to official documents may be considered to be in the public interest. Personal data in documents held by a public authority or a public body should be able to be publicly disclosed by that authority or body if the disclosure is provided for by Union or Member State law to which the public authority or public body is subject. Such laws should reconcile public access to official documents and the reuse of public sector information with the right to the protection of personal data and may therefore provide for the necessary reconciliation with the right to the protection of personal data pursuant to this Regulation. The reference to public authorities and bodies should in that context include all authorities or other bodies covered by Member State law on public access to documents. Directive 2003/98/EC of the European Parliament and of the Council [1] leaves intact and in no way affects the level of protection of natural persons with regard to the

(1) Directive 2003/98/EC of the European Parliament and of the Council of 17 November 2003 on the re-use of public sector information (OJ L 345, 31.12.2003, p. 90).

processing of personal data under the provisions of Union and Member State law, and in particular does not alter the obligations and rights set out in this Regulation. In particular, that Directive should not apply to documents to which access is excluded or restricted by virtue of the access regimes on the grounds of protection of personal data, and parts of documents accessible by virtue of those regimes which contain personal data the re-use of which has been provided for by law as being incompatible with the law concerning the protection of natural persons with regard to the processing of personal data.

(155) Member State law or collective agreements, including 'works agreements', may provide for specific rules on the processing of employees' personal data in the employment context, in particular for the conditions under which personal data in the employment context may be processed on the basis of the consent of the employee, the purposes of the recruitment, the performance of the contract of employment, including discharge of obligations laid down by law or by collective agreements, management, planning and organisation of work, equality and diversity in the workplace, health and safety at work, and for the purposes of the exercise and enjoyment, on an individual or collective basis, of rights and benefits related to employment, and for the purpose of the termination of the employment relationship.

(156) The processing of personal data for archiving purposes in the public interest, scientific or historical research purposes or statistical purposes should be subject to appropriate safeguards for the rights and freedoms of the data subject pursuant to this Regulation. Those safeguards should ensure that technical and organisational measures are in place in order to ensure, in particular, the principle of data minimisation. The further processing of personal data for archiving purposes in the public interest, scientific or historical research purposes or statistical purposes is to be carried out when the controller has assessed the feasibility to fulfil those purposes by processing data which do not permit or no longer permit the identification of data subjects, provided that appropriate safeguards exist (such as, for instance, pseudonymisation of the data). Member States should provide for appropriate safeguards for the processing of personal data for archiving purposes in the public interest, scientific or historical research purposes or statistical purposes. Member States should be authorised to provide, under specific conditions and subject to appropriate safeguards for data subjects, specifications and derogations with regard to the information requirements and rights to rectification, to erasure, to be forgotten, to restriction of processing, to data portability, and to object when processing personal data for archiving purposes in the public interest, scientific or historical research purposes or statistical purposes. The conditions and safeguards in question may entail specific procedures for data subjects to exercise those rights if this is appropriate in the light of the purposes sought by the specific processing along with technical and organisational measures aimed at minimising the processing of personal data in pursuance of the proportionality and necessity principles. The processing of personal data for scientific purposes should also comply with other relevant legislation such as on clinical trials.

(157) By coupling information from registries, researchers can obtain new knowledge of great value with regard to widespread medical conditions such as cardiovascular disease, cancer and depression. On the basis of registries, research results can be enhanced, as they draw on a larger population. Within social science, research on the basis of registries enables researchers to obtain essential knowledge about the long-term correlation of a number of social conditions such as unemployment and education with other life conditions. Research results obtained through registries provide solid, high-quality knowledge which can provide the basis for the formulation and implementation of knowledge-based policy, improve the quality of life for a number of people and improve the efficiency of social services. In order to facilitate scientific research, personal data can be processed for scientific research purposes, subject to appropriate conditions and safeguards set out in Union or Member State law.

(158) Where personal data are processed for archiving purposes, this Regulation should also apply to that processing, bearing in mind that this Regulation should not apply to deceased persons. Public authorities or public or private bodies that hold records of public interest should be services which, pursuant to Union or Member State law, have a legal obligation to acquire, preserve, appraise, arrange, describe, communicate, promote, disseminate and provide access to records of enduring value for general public interest. Member States should also be authorised to provide for the further processing of personal data for archiving purposes, for example with a view to providing specific information related to the political behaviour under former totalitarian state regimes, genocide, crimes against humanity, in particular the Holocaust, or war crimes.

(159) Where personal data are processed for scientific research purposes, this Regulation should also apply to that processing. For the purposes of this Regulation, the processing of personal data for scientific research purposes should be interpreted in a broad manner including for example technological development and demonstration, fundamental research, applied research and privately funded research. In addition, it should take into account the Union's objective under Article 179(1) TFEU of achieving a European Research Area. Scientific research purposes should also include studies conducted in the public interest in the area of public health. To meet the specificities of processing personal data for scientific research purposes, specific conditions should apply in particular as regards the publication or otherwise disclosure of personal data in the context of scientific research purposes. If the result of scientific research in particular in the health context gives reason for further measures in the interest of the data subject, the general rules of this Regulation should apply in view of those measures.

(160) Where personal data are processed for historical research purposes, this Regulation should also apply to that processing. This should also include historical research and research for genealogical purposes, bearing in mind that this Regulation should not apply to deceased persons.

(161) For the purpose of consenting to the participation in scientific research activities in clinical trials, the relevant provisions of Regulation (EU) No 536/2014 of the European Parliament and of the Council ([1]) should apply.

(162) Where personal data are processed for statistical purposes, this Regulation should apply to that processing. Union or Member State law should, within the limits of this Regulation, determine statistical content, control of access, specifications for the processing of personal data for statistical purposes and appropriate measures to safeguard the rights and freedoms of the data subject and for ensuring statistical confidentiality. Statistical purposes mean any operation of collection and the processing of personal data necessary for statistical surveys or for the production of statistical results. Those statistical results may further be used for different purposes, including a scientific research purpose. The statistical purpose implies that the result of processing for statistical purposes is not personal data, but aggregate data, and that this result or the personal data are not used in support of measures or decisions regarding any particular natural person.

(163) The confidential information which the Union and national statistical authorities collect for the production of official European and official national statistics should be protected. European statistics should be developed, produced and disseminated in accordance with the statistical principles as set out in Article 338(2) TFEU, while national statistics should also comply with Member State law. Regulation (EC) No 223/2009 of the European Parliament and of the Council ([2]) provides further specifications on statistical confidentiality for European statistics.

(164) As regards the powers of the supervisory authorities to obtain from the controller or processor access to personal data and access to their premises, Member States may adopt by law, within the limits of this Regulation, specific rules in order to safeguard the professional or other equivalent secrecy obligations, in so far as necessary to reconcile the right to the protection of personal data with an obligation of professional secrecy. This is without prejudice to existing Member State obligations to adopt rules on professional secrecy where required by Union law.

(165) This Regulation respects and does not prejudice the status under existing constitutional law of churches and religious associations or communities in the Member States, as recognised in Article 17 TFEU.

(166) In order to fulfil the objectives of this Regulation, namely to protect the fundamental rights and freedoms of natural persons and in particular their right to the protection of personal data and to ensure the free movement

([1]) Regulation (EU) No 536/2014 of the European Parliament and of the Council of 16 April 2014 on clinical trials on medicinal products for human use, and repealing Directive 2001/20/EC (OJ L 158, 27.5.2014, p. 1).

([2]) Regulation (EC) No 223/2009 of the European Parliament and of the Council of 11 March 2009 on European statistics and repealing Regulation (EC, Euratom) No 1101/2008 of the European Parliament and of the Council on the transmission of data subject to statistical confidentiality to the Statistical Office of the European Communities, Council Regulation (EC) No 322/97 on Community Statistics, and Council Decision 89/382/EEC, Euratom establishing a Committee on the Statistical Programmes of the European Communities (OJ L 87, 31.3.2009, p. 164).

of personal data within the Union, the power to adopt acts in accordance with Article 290 TFEU should be delegated to the Commission. In particular, delegated acts should be adopted in respect of criteria and requirements for certification mechanisms, information to be presented by standardised icons and procedures for providing such icons. It is of particular importance that the Commission carry out appropriate consultations during its preparatory work, including at expert level. The Commission, when preparing and drawing-up delegated acts, should ensure a simultaneous, timely and appropriate transmission of relevant documents to the European Parliament and to the Council.

(167) In order to ensure uniform conditions for the implementation of this Regulation, implementing powers should be conferred on the Commission when provided for by this Regulation. Those powers should be exercised in accordance with Regulation (EU) No 182/2011. In that context, the Commission should consider specific measures for micro, small and medium-sized enterprises.

(168) The examination procedure should be used for the adoption of implementing acts on standard contractual clauses between controllers and processors and between processors; codes of conduct; technical standards and mechanisms for certification; the adequate level of protection afforded by a third country, a territory or a specified sector within that third country, or an international organisation; standard protection clauses; formats and procedures for the exchange of information by electronic means between controllers, processors and supervisory authorities for binding corporate rules; mutual assistance; and arrangements for the exchange of information by electronic means between supervisory authorities, and between supervisory authorities and the Board.

(169) The Commission should adopt immediately applicable implementing acts where available evidence reveals that a third country, a territory or a specified sector within that third country, or an international organisation does not ensure an adequate level of protection, and imperative grounds of urgency so require.

(170) Since the objective of this Regulation, namely to ensure an equivalent level of protection of natural persons and the free flow of personal data throughout the Union, cannot be sufficiently achieved by the Member States and can rather, by reason of the scale or effects of the action, be better achieved at Union level, the Union may adopt measures, in accordance with the principle of subsidiarity as set out in Article 5 of the Treaty on European Union (TEU). In accordance with the principle of proportionality as set out in that Article, this Regulation does not go beyond what is necessary in order to achieve that objective.

(171) Directive 95/46/EC should be repealed by this Regulation. Processing already under way on the date of application of this Regulation should be brought into conformity with this Regulation within the period of two years after which this Regulation enters into force. Where processing is based on consent pursuant to Directive 95/46/EC, it is not necessary for the data subject to give his or her consent again if the manner in which the consent has been given is in line with the conditions of this Regulation, so as to allow the controller to continue such processing after the date of application of this Regulation. Commission decisions adopted and authorisations by supervisory authorities based on Directive 95/46/EC remain in force until amended, replaced or repealed.

(172) The European Data Protection Supervisor was consulted in accordance with Article 28(2) of Regulation (EC) No 45/2001 and delivered an opinion on 7 March 2012 ([1]).

(173) This Regulation should apply to all matters concerning the protection of fundamental rights and freedoms *vis-à-vis* the processing of personal data which are not subject to specific obligations with the same objective set out in Directive 2002/58/EC of the European Parliament and of the Council ([2]), including the obligations on the controller and the rights of natural persons. In order to clarify the relationship between this Regulation and Directive 2002/58/EC, that Directive should be amended accordingly. Once this Regulation is adopted, Directive 2002/58/EC should be reviewed in particular in order to ensure consistency with this Regulation,

---

([1]) OJ C 192, 30.6.2012, p. 7.

([2]) Directive 2002/58/EC of the European Parliament and of the Council of 12 July 2002 concerning the processing of personal data and the protection of privacy in the electronic communications sector (Directive on privacy and electronic communications) (OJ L 201, 31.7.2002, p. 37).

HAVE ADOPTED THIS REGULATION:

CHAPTER I

General provisions

Article 1

Subject-matter and objectives

1. This Regulation lays down rules relating to the protection of natural persons with regard to the processing of personal data and rules relating to the free movement of personal data.

2. This Regulation protects fundamental rights and freedoms of natural persons and in particular their right to the protection of personal data.

3. The free movement of personal data within the Union shall be neither restricted nor prohibited for reasons connected with the protection of natural persons with regard to the processing of personal data.

Article 2

Material scope

1. This Regulation applies to the processing of personal data wholly or partly by automated means and to the processing other than by automated means of personal data which form part of a filing system or are intended to form part of a filing system.

2. This Regulation does not apply to the processing of personal data:

(a) in the course of an activity which falls outside the scope of Union law;

(b) by the Member States when carrying out activities which fall within the scope of Chapter 2 of Title V of the TEU;

(c) by a natural person in the course of a purely personal or household activity;

(d) by competent authorities for the purposes of the prevention, investigation, detection or prosecution of criminal offences or the execution of criminal penalties, including the safeguarding against and the prevention of threats to public security.

3. For the processing of personal data by the Union institutions, bodies, offices and agencies, Regulation (EC) No 45/2001 applies. Regulation (EC) No 45/2001 and other Union legal acts applicable to such processing of personal data shall be adapted to the principles and rules of this Regulation in accordance with Article 98.

4. This Regulation shall be without prejudice to the application of Directive 2000/31/EC, in particular of the liability rules of intermediary service providers in Articles 12 to 15 of that Directive.

Article 3

Territorial scope

1. This Regulation applies to the processing of personal data in the context of the activities of an establishment of a controller or a processor in the Union, regardless of whether the processing takes place in the Union or not.

2. This Regulation applies to the processing of personal data of data subjects who are in the Union by a controller or processor not established in the Union, where the processing activities are related to:

(a) the offering of goods or services, irrespective of whether a payment of the data subject is required, to such data subjects in the Union; or

(b) the monitoring of their behaviour as far as their behaviour takes place within the Union.

3. This Regulation applies to the processing of personal data by a controller not established in the Union, but in a place where Member State law applies by virtue of public international law.

Article 4

Definitions

For the purposes of this Regulation:

(1) 'personal data' means any information relating to an identified or identifiable natural person ('data subject'); an identifiable natural person is one who can be identified, directly or indirectly, in particular by reference to an identifier such as a name, an identification number, location data, an online identifier or to one or more factors specific to the physical, physiological, genetic, mental, economic, cultural or social identity of that natural person;

(2) 'processing' means any operation or set of operations which is performed on personal data or on sets of personal data, whether or not by automated means, such as collection, recording, organisation, structuring, storage, adaptation or alteration, retrieval, consultation, use, disclosure by transmission, dissemination or otherwise making available, alignment or combination, restriction, erasure or destruction;

(3) 'restriction of processing' means the marking of stored personal data with the aim of limiting their processing in the future;

(4) 'profiling' means any form of automated processing of personal data consisting of the use of personal data to evaluate certain personal aspects relating to a natural person, in particular to analyse or predict aspects concerning that natural person's performance at work, economic situation, health, personal preferences, interests, reliability, behaviour, location or movements;

(5) 'pseudonymisation' means the processing of personal data in such a manner that the personal data can no longer be attributed to a specific data subject without the use of additional information, provided that such additional information is kept separately and is subject to technical and organisational measures to ensure that the personal data are not attributed to an identified or identifiable natural person;

(6) 'filing system' means any structured set of personal data which are accessible according to specific criteria, whether centralised, decentralised or dispersed on a functional or geographical basis;

(7) 'controller' means the natural or legal person, public authority, agency or other body which, alone or jointly with others, determines the purposes and means of the processing of personal data; where the purposes and means of such processing are determined by Union or Member State law, the controller or the specific criteria for its nomination may be provided for by Union or Member State law;

(8) 'processor' means a natural or legal person, public authority, agency or other body which processes personal data on behalf of the controller;

(9) 'recipient' means a natural or legal person, public authority, agency or another body, to which the personal data are disclosed, whether a third party or not. However, public authorities which may receive personal data in the

framework of a particular inquiry in accordance with Union or Member State law shall not be regarded as recipients; the processing of those data by those public authorities shall be in compliance with the applicable data protection rules according to the purposes of the processing;

(10) 'third party' means a natural or legal person, public authority, agency or body other than the data subject, controller, processor and persons who, under the direct authority of the controller or processor, are authorised to process personal data;

(11) 'consent' of the data subject means any freely given, specific, informed and unambiguous indication of the data subject's wishes by which he or she, by a statement or by a clear affirmative action, signifies agreement to the processing of personal data relating to him or her;

(12) 'personal data breach' means a breach of security leading to the accidental or unlawful destruction, loss, alteration, unauthorised disclosure of, or access to, personal data transmitted, stored or otherwise processed;

(13) 'genetic data' means personal data relating to the inherited or acquired genetic characteristics of a natural person which give unique information about the physiology or the health of that natural person and which result, in particular, from an analysis of a biological sample from the natural person in question;

(14) 'biometric data' means personal data resulting from specific technical processing relating to the physical, physiological or behavioural characteristics of a natural person, which allow or confirm the unique identification of that natural person, such as facial images or dactyloscopic data;

(15) 'data concerning health' means personal data related to the physical or mental health of a natural person, including the provision of health care services, which reveal information about his or her health status;

(16) 'main establishment' means:

(a) as regards a controller with establishments in more than one Member State, the place of its central administration in the Union, unless the decisions on the purposes and means of the processing of personal data are taken in another establishment of the controller in the Union and the latter establishment has the power to have such decisions implemented, in which case the establishment having taken such decisions is to be considered to be the main establishment;

(b) as regards a processor with establishments in more than one Member State, the place of its central administration in the Union, or, if the processor has no central administration in the Union, the establishment of the processor in the Union where the main processing activities in the context of the activities of an establishment of the processor take place to the extent that the processor is subject to specific obligations under this Regulation;

(17) 'representative' means a natural or legal person established in the Union who, designated by the controller or processor in writing pursuant to Article 27, represents the controller or processor with regard to their respective obligations under this Regulation;

(18) 'enterprise' means a natural or legal person engaged in an economic activity, irrespective of its legal form, including partnerships or associations regularly engaged in an economic activity;

(19) 'group of undertakings' means a controlling undertaking and its controlled undertakings;

(20) 'binding corporate rules' means personal data protection policies which are adhered to by a controller or processor established on the territory of a Member State for transfers or a set of transfers of personal data to a controller or processor in one or more third countries within a group of undertakings, or group of enterprises engaged in a joint economic activity;

(21) 'supervisory authority' means an independent public authority which is established by a Member State pursuant to Article 51;

(22) 'supervisory authority concerned' means a supervisory authority which is concerned by the processing of personal data because:

(a) the controller or processor is established on the territory of the Member State of that supervisory authority;

(b) data subjects residing in the Member State of that supervisory authority are substantially affected or likely to be substantially affected by the processing; or

(c) a complaint has been lodged with that supervisory authority;

(23) 'cross-border processing' means either:

(a) processing of personal data which takes place in the context of the activities of establishments in more than one Member State of a controller or processor in the Union where the controller or processor is established in more than one Member State; or

(b) processing of personal data which takes place in the context of the activities of a single establishment of a controller or processor in the Union but which substantially affects or is likely to substantially affect data subjects in more than one Member State.

(24) 'relevant and reasoned objection' means an objection to a draft decision as to whether there is an infringement of this Regulation, or whether envisaged action in relation to the controller or processor complies with this Regulation, which clearly demonstrates the significance of the risks posed by the draft decision as regards the fundamental rights and freedoms of data subjects and, where applicable, the free flow of personal data within the Union;

(25) 'information society service' means a service as defined in point (b) of Article 1(1) of Directive (EU) 2015/1535 of the European Parliament and of the Council ([1]);

(26) 'international organisation' means an organisation and its subordinate bodies governed by public international law, or any other body which is set up by, or on the basis of, an agreement between two or more countries.

CHAPTER II

Principles

Article 5

Principles relating to processing of personal data

1. Personal data shall be:

(a) processed lawfully, fairly and in a transparent manner in relation to the data subject ('lawfulness, fairness and transparency');

(b) collected for specified, explicit and legitimate purposes and not further processed in a manner that is incompatible with those purposes; further processing for archiving purposes in the public interest, scientific or historical research purposes or statistical purposes shall, in accordance with Article 89(1), not be considered to be incompatible with the initial purposes ('purpose limitation');

(c) adequate, relevant and limited to what is necessary in relation to the purposes for which they are processed ('data minimisation');

(d) accurate and, where necessary, kept up to date; every reasonable step must be taken to ensure that personal data that are inaccurate, having regard to the purposes for which they are processed, are erased or rectified without delay ('accuracy');

([1]) Directive (EU) 2015/1535 of the European Parliament and of the Council of 9 September 2015 laying down a procedure for the provision of information in the field of technical regulations and of rules on Information Society services (OJ L 241, 17.9.2015, p. 1).

(e) kept in a form which permits identification of data subjects for no longer than is necessary for the purposes for which the personal data are processed; personal data may be stored for longer periods insofar as the personal data will be processed solely for archiving purposes in the public interest, scientific or historical research purposes or statistical purposes in accordance with Article 89(1) subject to implementation of the appropriate technical and organisational measures required by this Regulation in order to safeguard the rights and freedoms of the data subject ('storage limitation');

(f) processed in a manner that ensures appropriate security of the personal data, including protection against unauthorised or unlawful processing and against accidental loss, destruction or damage, using appropriate technical or organisational measures ('integrity and confidentiality').

2. The controller shall be responsible for, and be able to demonstrate compliance with, paragraph 1 ('accountability').

Article 6

Lawfulness of processing

1. Processing shall be lawful only if and to the extent that at least one of the following applies:

(a) the data subject has given consent to the processing of his or her personal data for one or more specific purposes;

(b) processing is necessary for the performance of a contract to which the data subject is party or in order to take steps at the request of the data subject prior to entering into a contract;

(c) processing is necessary for compliance with a legal obligation to which the controller is subject;

(d) processing is necessary in order to protect the vital interests of the data subject or of another natural person;

(e) processing is necessary for the performance of a task carried out in the public interest or in the exercise of official authority vested in the controller;

(f) processing is necessary for the purposes of the legitimate interests pursued by the controller or by a third party, except where such interests are overridden by the interests or fundamental rights and freedoms of the data subject which require protection of personal data, in particular where the data subject is a child.

Point (f) of the first subparagraph shall not apply to processing carried out by public authorities in the performance of their tasks.

2. Member States may maintain or introduce more specific provisions to adapt the application of the rules of this Regulation with regard to processing for compliance with points (c) and (e) of paragraph 1 by determining more precisely specific requirements for the processing and other measures to ensure lawful and fair processing including for other specific processing situations as provided for in Chapter IX.

3. The basis for the processing referred to in point (c) and (e) of paragraph 1 shall be laid down by:

(a) Union law; or

(b) Member State law to which the controller is subject.

The purpose of the processing shall be determined in that legal basis or, as regards the processing referred to in point (e) of paragraph 1, shall be necessary for the performance of a task carried out in the public interest or in the exercise of official authority vested in the controller. That legal basis may contain specific provisions to adapt the application of rules of this Regulation, inter alia: the general conditions governing the lawfulness of processing by the controller; the types of data which are subject to the processing; the data subjects concerned; the entities to, and the purposes for which, the personal data may be disclosed; the purpose limitation; storage periods; and processing operations and processing procedures, including measures to ensure lawful and fair processing such as those for other specific

processing situations as provided for in Chapter IX. The Union or the Member State law shall meet an objective of public interest and be proportionate to the legitimate aim pursued.

4. Where the processing for a purpose other than that for which the personal data have been collected is not based on the data subject's consent or on a Union or Member State law which constitutes a necessary and proportionate measure in a democratic society to safeguard the objectives referred to in Article 23(1), the controller shall, in order to ascertain whether processing for another purpose is compatible with the purpose for which the personal data are initially collected, take into account, inter alia:

(a) any link between the purposes for which the personal data have been collected and the purposes of the intended further processing;

(b) the context in which the personal data have been collected, in particular regarding the relationship between data subjects and the controller;

(c) the nature of the personal data, in particular whether special categories of personal data are processed, pursuant to Article 9, or whether personal data related to criminal convictions and offences are processed, pursuant to Article 10;

(d) the possible consequences of the intended further processing for data subjects;

(e) the existence of appropriate safeguards, which may include encryption or pseudonymisation.

Article 7

Conditions for consent

1. Where processing is based on consent, the controller shall be able to demonstrate that the data subject has consented to processing of his or her personal data.

2. If the data subject's consent is given in the context of a written declaration which also concerns other matters, the request for consent shall be presented in a manner which is clearly distinguishable from the other matters, in an intelligible and easily accessible form, using clear and plain language. Any part of such a declaration which constitutes an infringement of this Regulation shall not be binding.

3. The data subject shall have the right to withdraw his or her consent at any time. The withdrawal of consent shall not affect the lawfulness of processing based on consent before its withdrawal. Prior to giving consent, the data subject shall be informed thereof. It shall be as easy to withdraw as to give consent.

4. When assessing whether consent is freely given, utmost account shall be taken of whether, *inter alia*, the performance of a contract, including the provision of a service, is conditional on consent to the processing of personal data that is not necessary for the performance of that contract.

Article 8

Conditions applicable to child's consent in relation to information society services

1. Where point (a) of Article 6(1) applies, in relation to the offer of information society services directly to a child, the processing of the personal data of a child shall be lawful where the child is at least 16 years old. Where the child is below the age of 16 years, such processing shall be lawful only if and to the extent that consent is given or authorised by the holder of parental responsibility over the child.

Member States may provide by law for a lower age for those purposes provided that such lower age is not below 13 years.

2. The controller shall make reasonable efforts to verify in such cases that consent is given or authorised by the holder of parental responsibility over the child, taking into consideration available technology.

3. Paragraph 1 shall not affect the general contract law of Member States such as the rules on the validity, formation or effect of a contract in relation to a child.

Article 9

Processing of special categories of personal data

1. Processing of personal data revealing racial or ethnic origin, political opinions, religious or philosophical beliefs, or trade union membership, and the processing of genetic data, biometric data for the purpose of uniquely identifying a natural person, data concerning health or data concerning a natural person's sex life or sexual orientation shall be prohibited.

2. Paragraph 1 shall not apply if one of the following applies:

(a) the data subject has given explicit consent to the processing of those personal data for one or more specified purposes, except where Union or Member State law provide that the prohibition referred to in paragraph 1 may not be lifted by the data subject;

(b) processing is necessary for the purposes of carrying out the obligations and exercising specific rights of the controller or of the data subject in the field of employment and social security and social protection law in so far as it is authorised by Union or Member State law or a collective agreement pursuant to Member State law providing for appropriate safeguards for the fundamental rights and the interests of the data subject;

(c) processing is necessary to protect the vital interests of the data subject or of another natural person where the data subject is physically or legally incapable of giving consent;

(d) processing is carried out in the course of its legitimate activities with appropriate safeguards by a foundation, association or any other not-for-profit body with a political, philosophical, religious or trade union aim and on condition that the processing relates solely to the members or to former members of the body or to persons who have regular contact with it in connection with its purposes and that the personal data are not disclosed outside that body without the consent of the data subjects;

(e) processing relates to personal data which are manifestly made public by the data subject;

(f) processing is necessary for the establishment, exercise or defence of legal claims or whenever courts are acting in their judicial capacity;

(g) processing is necessary for reasons of substantial public interest, on the basis of Union or Member State law which shall be proportionate to the aim pursued, respect the essence of the right to data protection and provide for suitable and specific measures to safeguard the fundamental rights and the interests of the data subject;

(h) processing is necessary for the purposes of preventive or occupational medicine, for the assessment of the working capacity of the employee, medical diagnosis, the provision of health or social care or treatment or the management of health or social care systems and services on the basis of Union or Member State law or pursuant to contract with a health professional and subject to the conditions and safeguards referred to in paragraph 3;

(i) processing is necessary for reasons of public interest in the area of public health, such as protecting against serious cross-border threats to health or ensuring high standards of quality and safety of health care and of medicinal products or medical devices, on the basis of Union or Member State law which provides for suitable and specific measures to safeguard the rights and freedoms of the data subject, in particular professional secrecy;

(j) processing is necessary for archiving purposes in the public interest, scientific or historical research purposes or statistical purposes in accordance with Article 89(1) based on Union or Member State law which shall be proportionate to the aim pursued, respect the essence of the right to data protection and provide for suitable and specific measures to safeguard the fundamental rights and the interests of the data subject.

3. Personal data referred to in paragraph 1 may be processed for the purposes referred to in point (h) of paragraph 2 when those data are processed by or under the responsibility of a professional subject to the obligation of professional secrecy under Union or Member State law or rules established by national competent bodies or by another person also subject to an obligation of secrecy under Union or Member State law or rules established by national competent bodies.

4. Member States may maintain or introduce further conditions, including limitations, with regard to the processing of genetic data, biometric data or data concerning health.

Article 10

Processing of personal data relating to criminal convictions and offences

Processing of personal data relating to criminal convictions and offences or related security measures based on Article 6(1) shall be carried out only under the control of official authority or when the processing is authorised by Union or Member State law providing for appropriate safeguards for the rights and freedoms of data subjects. Any comprehensive register of criminal convictions shall be kept only under the control of official authority.

Article 11

Processing which does not require identification

1. If the purposes for which a controller processes personal data do not or do no longer require the identification of a data subject by the controller, the controller shall not be obliged to maintain, acquire or process additional information in order to identify the data subject for the sole purpose of complying with this Regulation.

2. Where, in cases referred to in paragraph 1 of this Article, the controller is able to demonstrate that it is not in a position to identify the data subject, the controller shall inform the data subject accordingly, if possible. In such cases, Articles 15 to 20 shall not apply except where the data subject, for the purpose of exercising his or her rights under those articles, provides additional information enabling his or her identification.

CHAPTER III

Rights of the data subject

Section 1

Transparency and modalities

Article 12

Transparent information, communication and modalities for the exercise of the rights of the data subject

1. The controller shall take appropriate measures to provide any information referred to in Articles 13 and 14 and any communication under Articles 15 to 22 and 34 relating to processing to the data subject in a concise, transparent, intelligible and easily accessible form, using clear and plain language, in particular for any information addressed specifically to a child. The information shall be provided in writing, or by other means, including, where appropriate, by electronic means. When requested by the data subject, the information may be provided orally, provided that the identity of the data subject is proven by other means.

2. The controller shall facilitate the exercise of data subject rights under Articles 15 to 22. In the cases referred to in Article 11(2), the controller shall not refuse to act on the request of the data subject for exercising his or her rights under Articles 15 to 22, unless the controller demonstrates that it is not in a position to identify the data subject.

3. The controller shall provide information on action taken on a request under Articles 15 to 22 to the data subject without undue delay and in any event within one month of receipt of the request. That period may be extended by two further months where necessary, taking into account the complexity and number of the requests. The controller shall inform the data subject of any such extension within one month of receipt of the request, together with the reasons for the delay. Where the data subject makes the request by electronic form means, the information shall be provided by electronic means where possible, unless otherwise requested by the data subject.

4. If the controller does not take action on the request of the data subject, the controller shall inform the data subject without delay and at the latest within one month of receipt of the request of the reasons for not taking action and on the possibility of lodging a complaint with a supervisory authority and seeking a judicial remedy.

5. Information provided under Articles 13 and 14 and any communication and any actions taken under Articles 15 to 22 and 34 shall be provided free of charge. Where requests from a data subject are manifestly unfounded or excessive, in particular because of their repetitive character, the controller may either:

(a) charge a reasonable fee taking into account the administrative costs of providing the information or communication or taking the action requested; or

(b) refuse to act on the request.

The controller shall bear the burden of demonstrating the manifestly unfounded or excessive character of the request.

6. Without prejudice to Article 11, where the controller has reasonable doubts concerning the identity of the natural person making the request referred to in Articles 15 to 21, the controller may request the provision of additional information necessary to confirm the identity of the data subject.

7. The information to be provided to data subjects pursuant to Articles 13 and 14 may be provided in combination with standardised icons in order to give in an easily visible, intelligible and clearly legible manner a meaningful overview of the intended processing. Where the icons are presented electronically they shall be machine-readable.

8. The Commission shall be empowered to adopt delegated acts in accordance with Article 92 for the purpose of determining the information to be presented by the icons and the procedures for providing standardised icons.

Section 2

Information and access to personal data

Article 13

Information to be provided where personal data are collected from the data subject

1. Where personal data relating to a data subject are collected from the data subject, the controller shall, at the time when personal data are obtained, provide the data subject with all of the following information:

(a) the identity and the contact details of the controller and, where applicable, of the controller's representative;

(b) the contact details of the data protection officer, where applicable;

(c) the purposes of the processing for which the personal data are intended as well as the legal basis for the processing;

(d) where the processing is based on point (f) of Article 6(1), the legitimate interests pursued by the controller or by a third party;

(e) the recipients or categories of recipients of the personal data, if any;

(f) where applicable, the fact that the controller intends to transfer personal data to a third country or international organisation and the existence or absence of an adequacy decision by the Commission, or in the case of transfers referred to in Article 46 or 47, or the second subparagraph of Article 49(1), reference to the appropriate or suitable safeguards and the means by which to obtain a copy of them or where they have been made available.

2. In addition to the information referred to in paragraph 1, the controller shall, at the time when personal data are obtained, provide the data subject with the following further information necessary to ensure fair and transparent processing:

(a) the period for which the personal data will be stored, or if that is not possible, the criteria used to determine that period;

(b) the existence of the right to request from the controller access to and rectification or erasure of personal data or restriction of processing concerning the data subject or to object to processing as well as the right to data portability;

(c) where the processing is based on point (a) of Article 6(1) or point (a) of Article 9(2), the existence of the right to withdraw consent at any time, without affecting the lawfulness of processing based on consent before its withdrawal;

(d) the right to lodge a complaint with a supervisory authority;

(e) whether the provision of personal data is a statutory or contractual requirement, or a requirement necessary to enter into a contract, as well as whether the data subject is obliged to provide the personal data and of the possible consequences of failure to provide such data;

(f) the existence of automated decision-making, including profiling, referred to in Article 22(1) and (4) and, at least in those cases, meaningful information about the logic involved, as well as the significance and the envisaged consequences of such processing for the data subject.

3. Where the controller intends to further process the personal data for a purpose other than that for which the personal data were collected, the controller shall provide the data subject prior to that further processing with information on that other purpose and with any relevant further information as referred to in paragraph 2.

4. Paragraphs 1, 2 and 3 shall not apply where and insofar as the data subject already has the information.

Article 14

Information to be provided where personal data have not been obtained from the data subject

1. Where personal data have not been obtained from the data subject, the controller shall provide the data subject with the following information:

(a) the identity and the contact details of the controller and, where applicable, of the controller's representative;

(b) the contact details of the data protection officer, where applicable;

(c) the purposes of the processing for which the personal data are intended as well as the legal basis for the processing;

(d) the categories of personal data concerned;

(e) the recipients or categories of recipients of the personal data, if any;

(f) where applicable, that the controller intends to transfer personal data to a recipient in a third country or international organisation and the existence or absence of an adequacy decision by the Commission, or in the case of transfers referred to in Article 46 or 47, or the second subparagraph of Article 49(1), reference to the appropriate or suitable safeguards and the means to obtain a copy of them or where they have been made available.

2. In addition to the information referred to in paragraph 1, the controller shall provide the data subject with the following information necessary to ensure fair and transparent processing in respect of the data subject:

(a) the period for which the personal data will be stored, or if that is not possible, the criteria used to determine that period;

(b) where the processing is based on point (f) of Article 6(1), the legitimate interests pursued by the controller or by a third party;

(c) the existence of the right to request from the controller access to and rectification or erasure of personal data or restriction of processing concerning the data subject and to object to processing as well as the right to data portability;

(d) where processing is based on point (a) of Article 6(1) or point (a) of Article 9(2), the existence of the right to withdraw consent at any time, without affecting the lawfulness of processing based on consent before its withdrawal;

(e) the right to lodge a complaint with a supervisory authority;

(f) from which source the personal data originate, and if applicable, whether it came from publicly accessible sources;

(g) the existence of automated decision-making, including profiling, referred to in Article 22(1) and (4) and, at least in those cases, meaningful information about the logic involved, as well as the significance and the envisaged consequences of such processing for the data subject.

3. The controller shall provide the information referred to in paragraphs 1 and 2:

(a) within a reasonable period after obtaining the personal data, but at the latest within one month, having regard to the specific circumstances in which the personal data are processed;

(b) if the personal data are to be used for communication with the data subject, at the latest at the time of the first communication to that data subject; or

(c) if a disclosure to another recipient is envisaged, at the latest when the personal data are first disclosed.

4. Where the controller intends to further process the personal data for a purpose other than that for which the personal data were obtained, the controller shall provide the data subject prior to that further processing with information on that other purpose and with any relevant further information as referred to in paragraph 2.

5. Paragraphs 1 to 4 shall not apply where and insofar as:

(a) the data subject already has the information;

(b) the provision of such information proves impossible or would involve a disproportionate effort, in particular for processing for archiving purposes in the public interest, scientific or historical research purposes or statistical purposes, subject to the conditions and safeguards referred to in Article 89(1) or in so far as the obligation referred to in paragraph 1 of this Article is likely to render impossible or seriously impair the achievement of the objectives of that processing. In such cases the controller shall take appropriate measures to protect the data subject's rights and freedoms and legitimate interests, including making the information publicly available;

(c) obtaining or disclosure is expressly laid down by Union or Member State law to which the controller is subject and which provides appropriate measures to protect the data subject's legitimate interests; or

(d) where the personal data must remain confidential subject to an obligation of professional secrecy regulated by Union or Member State law, including a statutory obligation of secrecy.

Article 15

Right of access by the data subject

1. The data subject shall have the right to obtain from the controller confirmation as to whether or not personal data concerning him or her are being processed, and, where that is the case, access to the personal data and the following information:

(a) the purposes of the processing;

(b) the categories of personal data concerned;

(c) the recipients or categories of recipient to whom the personal data have been or will be disclosed, in particular recipients in third countries or international organisations;

(d) where possible, the envisaged period for which the personal data will be stored, or, if not possible, the criteria used to determine that period;

(e) the existence of the right to request from the controller rectification or erasure of personal data or restriction of processing of personal data concerning the data subject or to object to such processing;

(f) the right to lodge a complaint with a supervisory authority;

(g) where the personal data are not collected from the data subject, any available information as to their source;

(h) the existence of automated decision-making, including profiling, referred to in Article 22(1) and (4) and, at least in those cases, meaningful information about the logic involved, as well as the significance and the envisaged consequences of such processing for the data subject.

2. Where personal data are transferred to a third country or to an international organisation, the data subject shall have the right to be informed of the appropriate safeguards pursuant to Article 46 relating to the transfer.

3. The controller shall provide a copy of the personal data undergoing processing. For any further copies requested by the data subject, the controller may charge a reasonable fee based on administrative costs. Where the data subject makes the request by electronic means, and unless otherwise requested by the data subject, the information shall be provided in a commonly used electronic form.

4. The right to obtain a copy referred to in paragraph 3 shall not adversely affect the rights and freedoms of others.

Section 3

Rectification and erasure

Article 16

Right to rectification

The data subject shall have the right to obtain from the controller without undue delay the rectification of inaccurate personal data concerning him or her. Taking into account the purposes of the processing, the data subject shall have the right to have incomplete personal data completed, including by means of providing a supplementary statement.

Article 17

Right to erasure ('right to be forgotten')

1. The data subject shall have the right to obtain from the controller the erasure of personal data concerning him or her without undue delay and the controller shall have the obligation to erase personal data without undue delay where one of the following grounds applies:

(a) the personal data are no longer necessary in relation to the purposes for which they were collected or otherwise processed;

(b) the data subject withdraws consent on which the processing is based according to point (a) of Article 6(1), or point (a) of Article 9(2), and where there is no other legal ground for the processing;

(c) the data subject objects to the processing pursuant to Article 21(1) and there are no overriding legitimate grounds for the processing, or the data subject objects to the processing pursuant to Article 21(2);

(d) the personal data have been unlawfully processed;

(e) the personal data have to be erased for compliance with a legal obligation in Union or Member State law to which the controller is subject;

(f) the personal data have been collected in relation to the offer of information society services referred to in Article 8(1).

2. Where the controller has made the personal data public and is obliged pursuant to paragraph 1 to erase the personal data, the controller, taking account of available technology and the cost of implementation, shall take reasonable steps, including technical measures, to inform controllers which are processing the personal data that the data subject has requested the erasure by such controllers of any links to, or copy or replication of, those personal data.

3. Paragraphs 1 and 2 shall not apply to the extent that processing is necessary:

(a) for exercising the right of freedom of expression and information;

(b) for compliance with a legal obligation which requires processing by Union or Member State law to which the controller is subject or for the performance of a task carried out in the public interest or in the exercise of official authority vested in the controller;

(c) for reasons of public interest in the area of public health in accordance with points (h) and (i) of Article 9(2) as well as Article 9(3);

(d) for archiving purposes in the public interest, scientific or historical research purposes or statistical purposes in accordance with Article 89(1) in so far as the right referred to in paragraph 1 is likely to render impossible or seriously impair the achievement of the objectives of that processing; or

(e) for the establishment, exercise or defence of legal claims.

Article 18

Right to restriction of processing

1. The data subject shall have the right to obtain from the controller restriction of processing where one of the following applies:

(a) the accuracy of the personal data is contested by the data subject, for a period enabling the controller to verify the accuracy of the personal data;

(b) the processing is unlawful and the data subject opposes the erasure of the personal data and requests the restriction of their use instead;

(c) the controller no longer needs the personal data for the purposes of the processing, but they are required by the data subject for the establishment, exercise or defence of legal claims;

(d) the data subject has objected to processing pursuant to Article 21(1) pending the verification whether the legitimate grounds of the controller override those of the data subject.

2. Where processing has been restricted under paragraph 1, such personal data shall, with the exception of storage, only be processed with the data subject's consent or for the establishment, exercise or defence of legal claims or for the protection of the rights of another natural or legal person or for reasons of important public interest of the Union or of a Member State.

3. A data subject who has obtained restriction of processing pursuant to paragraph 1 shall be informed by the controller before the restriction of processing is lifted.

Article 19

Notification obligation regarding rectification or erasure of personal data or restriction of processing

The controller shall communicate any rectification or erasure of personal data or restriction of processing carried out in accordance with Article 16, Article 17(1) and Article 18 to each recipient to whom the personal data have been disclosed, unless this proves impossible or involves disproportionate effort. The controller shall inform the data subject about those recipients if the data subject requests it.

Article 20

Right to data portability

1. The data subject shall have the right to receive the personal data concerning him or her, which he or she has provided to a controller, in a structured, commonly used and machine-readable format and have the right to transmit those data to another controller without hindrance from the controller to which the personal data have been provided, where:

(a) the processing is based on consent pursuant to point (a) of Article 6(1) or point (a) of Article 9(2) or on a contract pursuant to point (b) of Article 6(1); and

(b) the processing is carried out by automated means.

2. In exercising his or her right to data portability pursuant to paragraph 1, the data subject shall have the right to have the personal data transmitted directly from one controller to another, where technically feasible.

3. The exercise of the right referred to in paragraph 1 of this Article shall be without prejudice to Article 17. That right shall not apply to processing necessary for the performance of a task carried out in the public interest or in the exercise of official authority vested in the controller.

4. The right referred to in paragraph 1 shall not adversely affect the rights and freedoms of others.

Section 4

Right to object and automated individual decision-making

Article 21

Right to object

1. The data subject shall have the right to object, on grounds relating to his or her particular situation, at any time to processing of personal data concerning him or her which is based on point (e) or (f) of Article 6(1), including profiling based on those provisions. The controller shall no longer process the personal data unless the controller demonstrates compelling legitimate grounds for the processing which override the interests, rights and freedoms of the data subject or for the establishment, exercise or defence of legal claims.

2. Where personal data are processed for direct marketing purposes, the data subject shall have the right to object at any time to processing of personal data concerning him or her for such marketing, which includes profiling to the extent that it is related to such direct marketing.

3. Where the data subject objects to processing for direct marketing purposes, the personal data shall no longer be processed for such purposes.

4. At the latest at the time of the first communication with the data subject, the right referred to in paragraphs 1 and 2 shall be explicitly brought to the attention of the data subject and shall be presented clearly and separately from any other information.

5. In the context of the use of information society services, and notwithstanding Directive 2002/58/EC, the data subject may exercise his or her right to object by automated means using technical specifications.

6. Where personal data are processed for scientific or historical research purposes or statistical purposes pursuant to Article 89(1), the data subject, on grounds relating to his or her particular situation, shall have the right to object to processing of personal data concerning him or her, unless the processing is necessary for the performance of a task carried out for reasons of public interest.

Article 22

Automated individual decision-making, including profiling

1. The data subject shall have the right not to be subject to a decision based solely on automated processing, including profiling, which produces legal effects concerning him or her or similarly significantly affects him or her.

2. Paragraph 1 shall not apply if the decision:

(a) is necessary for entering into, or performance of, a contract between the data subject and a data controller;

(b) is authorised by Union or Member State law to which the controller is subject and which also lays down suitable measures to safeguard the data subject's rights and freedoms and legitimate interests; or

(c) is based on the data subject's explicit consent.

3. In the cases referred to in points (a) and (c) of paragraph 2, the data controller shall implement suitable measures to safeguard the data subject's rights and freedoms and legitimate interests, at least the right to obtain human intervention on the part of the controller, to express his or her point of view and to contest the decision.

4. Decisions referred to in paragraph 2 shall not be based on special categories of personal data referred to in Article 9(1), unless point (a) or (g) of Article 9(2) applies and suitable measures to safeguard the data subject's rights and freedoms and legitimate interests are in place.

Section 5

Restrictions

Article 23

Restrictions

1. Union or Member State law to which the data controller or processor is subject may restrict by way of a legislative measure the scope of the obligations and rights provided for in Articles 12 to 22 and Article 34, as well as Article 5 in so far as its provisions correspond to the rights and obligations provided for in Articles 12 to 22, when such a restriction respects the essence of the fundamental rights and freedoms and is a necessary and proportionate measure in a democratic society to safeguard:

(a) national security;

(b) defence;

(c) public security;

(d) the prevention, investigation, detection or prosecution of criminal offences or the execution of criminal penalties, including the safeguarding against and the prevention of threats to public security;

(e) other important objectives of general public interest of the Union or of a Member State, in particular an important economic or financial interest of the Union or of a Member State, including monetary, budgetary and taxation a matters, public health and social security;

(f) the protection of judicial independence and judicial proceedings;

(g) the prevention, investigation, detection and prosecution of breaches of ethics for regulated professions;

(h) a monitoring, inspection or regulatory function connected, even occasionally, to the exercise of official authority in the cases referred to in points (a) to (e) and (g);

(i) the protection of the data subject or the rights and freedoms of others;

(j) the enforcement of civil law claims.

2. In particular, any legislative measure referred to in paragraph 1 shall contain specific provisions at least, where relevant, as to:

(a) the purposes of the processing or categories of processing;

(b) the categories of personal data;

(c) the scope of the restrictions introduced;

(d) the safeguards to prevent abuse or unlawful access or transfer;

(e) the specification of the controller or categories of controllers;

(f) the storage periods and the applicable safeguards taking into account the nature, scope and purposes of the processing or categories of processing;

(g) the risks to the rights and freedoms of data subjects; and

(h) the right of data subjects to be informed about the restriction, unless that may be prejudicial to the purpose of the restriction.

CHAPTER IV

Controller and processor

Section 1

General obligations

Article 24

Responsibility of the controller

1. Taking into account the nature, scope, context and purposes of processing as well as the risks of varying likelihood and severity for the rights and freedoms of natural persons, the controller shall implement appropriate technical and organisational measures to ensure and to be able to demonstrate that processing is performed in accordance with this Regulation. Those measures shall be reviewed and updated where necessary.

2. Where proportionate in relation to processing activities, the measures referred to in paragraph 1 shall include the implementation of appropriate data protection policies by the controller.

3. Adherence to approved codes of conduct as referred to in Article 40 or approved certification mechanisms as referred to in Article 42 may be used as an element by which to demonstrate compliance with the obligations of the controller.

Article 25

Data protection by design and by default

1. Taking into account the state of the art, the cost of implementation and the nature, scope, context and purposes of processing as well as the risks of varying likelihood and severity for rights and freedoms of natural persons posed by the processing, the controller shall, both at the time of the determination of the means for processing and at the time of the processing itself, implement appropriate technical and organisational measures, such as pseudonymisation, which are designed to implement data-protection principles, such as data minimisation, in an effective manner and to integrate the necessary safeguards into the processing in order to meet the requirements of this Regulation and protect the rights of data subjects.

2. The controller shall implement appropriate technical and organisational measures for ensuring that, by default, only personal data which are necessary for each specific purpose of the processing are processed. That obligation applies to the amount of personal data collected, the extent of their processing, the period of their storage and their accessibility. In particular, such measures shall ensure that by default personal data are not made accessible without the individual's intervention to an indefinite number of natural persons.

3. An approved certification mechanism pursuant to Article 42 may be used as an element to demonstrate compliance with the requirements set out in paragraphs 1 and 2 of this Article.

Article 26

Joint controllers

1. Where two or more controllers jointly determine the purposes and means of processing, they shall be joint controllers. They shall in a transparent manner determine their respective responsibilities for compliance with the obligations under this Regulation, in particular as regards the exercising of the rights of the data subject and their respective duties to provide the information referred to in Articles 13 and 14, by means of an arrangement between them unless, and in so far as, the respective responsibilities of the controllers are determined by Union or Member State law to which the controllers are subject. The arrangement may designate a contact point for data subjects.

2. The arrangement referred to in paragraph 1 shall duly reflect the respective roles and relationships of the joint controllers *vis-à-vis* the data subjects. The essence of the arrangement shall be made available to the data subject.

3. Irrespective of the terms of the arrangement referred to in paragraph 1, the data subject may exercise his or her rights under this Regulation in respect of and against each of the controllers.

Article 27

Representatives of controllers or processors not established in the Union

1. Where Article 3(2) applies, the controller or the processor shall designate in writing a representative in the Union.

2. The obligation laid down in paragraph 1 of this Article shall not apply to:

(a) processing which is occasional, does not include, on a large scale, processing of special categories of data as referred to in Article 9(1) or processing of personal data relating to criminal convictions and offences referred to in Article 10, and is unlikely to result in a risk to the rights and freedoms of natural persons, taking into account the nature, context, scope and purposes of the processing; or

(b) a public authority or body.

3. The representative shall be established in one of the Member States where the data subjects, whose personal data are processed in relation to the offering of goods or services to them, or whose behaviour is monitored, are.

4. The representative shall be mandated by the controller or processor to be addressed in addition to or instead of the controller or the processor by, in particular, supervisory authorities and data subjects, on all issues related to processing, for the purposes of ensuring compliance with this Regulation.

5. The designation of a representative by the controller or processor shall be without prejudice to legal actions which could be initiated against the controller or the processor themselves.

Article 28

Processor

1. Where processing is to be carried out on behalf of a controller, the controller shall use only processors providing sufficient guarantees to implement appropriate technical and organisational measures in such a manner that processing will meet the requirements of this Regulation and ensure the protection of the rights of the data subject.

2. The processor shall not engage another processor without prior specific or general written authorisation of the controller. In the case of general written authorisation, the processor shall inform the controller of any intended changes concerning the addition or replacement of other processors, thereby giving the controller the opportunity to object to such changes.

3. Processing by a processor shall be governed by a contract or other legal act under Union or Member State law, that is binding on the processor with regard to the controller and that sets out the subject-matter and duration of the processing, the nature and purpose of the processing, the type of personal data and categories of data subjects and the obligations and rights of the controller. That contract or other legal act shall stipulate, in particular, that the processor:

(a) processes the personal data only on documented instructions from the controller, including with regard to transfers of personal data to a third country or an international organisation, unless required to do so by Union or Member State law to which the processor is subject; in such a case, the processor shall inform the controller of that legal requirement before processing, unless that law prohibits such information on important grounds of public interest;

(b) ensures that persons authorised to process the personal data have committed themselves to confidentiality or are under an appropriate statutory obligation of confidentiality;

(c) takes all measures required pursuant to Article 32;

(d) respects the conditions referred to in paragraphs 2 and 4 for engaging another processor;

(e) taking into account the nature of the processing, assists the controller by appropriate technical and organisational measures, insofar as this is possible, for the fulfilment of the controller's obligation to respond to requests for exercising the data subject's rights laid down in Chapter III;

(f) assists the controller in ensuring compliance with the obligations pursuant to Articles 32 to 36 taking into account the nature of processing and the information available to the processor;

(g) at the choice of the controller, deletes or returns all the personal data to the controller after the end of the provision of services relating to processing, and deletes existing copies unless Union or Member State law requires storage of the personal data;

(h) makes available to the controller all information necessary to demonstrate compliance with the obligations laid down in this Article and allow for and contribute to audits, including inspections, conducted by the controller or another auditor mandated by the controller.

With regard to point (h) of the first subparagraph, the processor shall immediately inform the controller if, in its opinion, an instruction infringes this Regulation or other Union or Member State data protection provisions.

4. Where a processor engages another processor for carrying out specific processing activities on behalf of the controller, the same data protection obligations as set out in the contract or other legal act between the controller and the processor as referred to in paragraph 3 shall be imposed on that other processor by way of a contract or other legal act under Union or Member State law, in particular providing sufficient guarantees to implement appropriate technical and organisational measures in such a manner that the processing will meet the requirements of this Regulation. Where that other processor fails to fulfil its data protection obligations, the initial processor shall remain fully liable to the controller for the performance of that other processor's obligations.

5. Adherence of a processor to an approved code of conduct as referred to in Article 40 or an approved certification mechanism as referred to in Article 42 may be used as an element by which to demonstrate sufficient guarantees as referred to in paragraphs 1 and 4 of this Article.

6. Without prejudice to an individual contract between the controller and the processor, the contract or the other legal act referred to in paragraphs 3 and 4 of this Article may be based, in whole or in part, on standard contractual clauses referred to in paragraphs 7 and 8 of this Article, including when they are part of a certification granted to the controller or processor pursuant to Articles 42 and 43.

7. The Commission may lay down standard contractual clauses for the matters referred to in paragraph 3 and 4 of this Article and in accordance with the examination procedure referred to in Article 93(2).

8. A supervisory authority may adopt standard contractual clauses for the matters referred to in paragraph 3 and 4 of this Article and in accordance with the consistency mechanism referred to in Article 63.

9. The contract or the other legal act referred to in paragraphs 3 and 4 shall be in writing, including in electronic form.

10. Without prejudice to Articles 82, 83 and 84, if a processor infringes this Regulation by determining the purposes and means of processing, the processor shall be considered to be a controller in respect of that processing.

Article 29

Processing under the authority of the controller or processor

The processor and any person acting under the authority of the controller or of the processor, who has access to personal data, shall not process those data except on instructions from the controller, unless required to do so by Union or Member State law.

Article 30

Records of processing activities

1. Each controller and, where applicable, the controller's representative, shall maintain a record of processing activities under its responsibility. That record shall contain all of the following information:

(a) the name and contact details of the controller and, where applicable, the joint controller, the controller's representative and the data protection officer;

(b) the purposes of the processing;

(c) a description of the categories of data subjects and of the categories of personal data;

(d) the categories of recipients to whom the personal data have been or will be disclosed including recipients in third countries or international organisations;

(e) where applicable, transfers of personal data to a third country or an international organisation, including the identification of that third country or international organisation and, in the case of transfers referred to in the second subparagraph of Article 49(1), the documentation of suitable safeguards;

(f) where possible, the envisaged time limits for erasure of the different categories of data;

(g) where possible, a general description of the technical and organisational security measures referred to in Article 32(1).

2. Each processor and, where applicable, the processor's representative shall maintain a record of all categories of processing activities carried out on behalf of a controller, containing:

(a) the name and contact details of the processor or processors and of each controller on behalf of which the processor is acting, and, where applicable, of the controller's or the processor's representative, and the data protection officer;

(b) the categories of processing carried out on behalf of each controller;

(c) where applicable, transfers of personal data to a third country or an international organisation, including the identification of that third country or international organisation and, in the case of transfers referred to in the second subparagraph of Article 49(1), the documentation of suitable safeguards;

(d) where possible, a general description of the technical and organisational security measures referred to in Article 32(1).

3. The records referred to in paragraphs 1 and 2 shall be in writing, including in electronic form.

4. The controller or the processor and, where applicable, the controller's or the processor's representative, shall make the record available to the supervisory authority on request.

5. The obligations referred to in paragraphs 1 and 2 shall not apply to an enterprise or an organisation employing fewer than 250 persons unless the processing it carries out is likely to result in a risk to the rights and freedoms of data subjects, the processing is not occasional, or the processing includes special categories of data as referred to in Article 9(1) or personal data relating to criminal convictions and offences referred to in Article 10.

Article 31

Cooperation with the supervisory authority

The controller and the processor and, where applicable, their representatives, shall cooperate, on request, with the supervisory authority in the performance of its tasks.

Section 2

Security of personal data

Article 32

Security of processing

1. Taking into account the state of the art, the costs of implementation and the nature, scope, context and purposes of processing as well as the risk of varying likelihood and severity for the rights and freedoms of natural persons, the controller and the processor shall implement appropriate technical and organisational measures to ensure a level of security appropriate to the risk, including inter alia as appropriate:

(a) the pseudonymisation and encryption of personal data;

(b) the ability to ensure the ongoing confidentiality, integrity, availability and resilience of processing systems and services;

(c) the ability to restore the availability and access to personal data in a timely manner in the event of a physical or technical incident;

(d) a process for regularly testing, assessing and evaluating the effectiveness of technical and organisational measures for ensuring the security of the processing.

2. In assessing the appropriate level of security account shall be taken in particular of the risks that are presented by processing, in particular from accidental or unlawful destruction, loss, alteration, unauthorised disclosure of, or access to personal data transmitted, stored or otherwise processed.

3. Adherence to an approved code of conduct as referred to in Article 40 or an approved certification mechanism as referred to in Article 42 may be used as an element by which to demonstrate compliance with the requirements set out in paragraph 1 of this Article.

4. The controller and processor shall take steps to ensure that any natural person acting under the authority of the controller or the processor who has access to personal data does not process them except on instructions from the controller, unless he or she is required to do so by Union or Member State law.

Article 33

Notification of a personal data breach to the supervisory authority

1. In the case of a personal data breach, the controller shall without undue delay and, where feasible, not later than 72 hours after having become aware of it, notify the personal data breach to the supervisory authority competent in accordance with Article 55, unless the personal data breach is unlikely to result in a risk to the rights and freedoms of natural persons. Where the notification to the supervisory authority is not made within 72 hours, it shall be accompanied by reasons for the delay.

2. The processor shall notify the controller without undue delay after becoming aware of a personal data breach.

3. The notification referred to in paragraph 1 shall at least:

(a) describe the nature of the personal data breach including where possible, the categories and approximate number of data subjects concerned and the categories and approximate number of personal data records concerned;

(b) communicate the name and contact details of the data protection officer or other contact point where more information can be obtained;

(c) describe the likely consequences of the personal data breach;

(d) describe the measures taken or proposed to be taken by the controller to address the personal data breach, including, where appropriate, measures to mitigate its possible adverse effects.

4. Where, and in so far as, it is not possible to provide the information at the same time, the information may be provided in phases without undue further delay.

5. The controller shall document any personal data breaches, comprising the facts relating to the personal data breach, its effects and the remedial action taken. That documentation shall enable the supervisory authority to verify compliance with this Article.

Article 34

Communication of a personal data breach to the data subject

1. When the personal data breach is likely to result in a high risk to the rights and freedoms of natural persons, the controller shall communicate the personal data breach to the data subject without undue delay.

2. The communication to the data subject referred to in paragraph 1 of this Article shall describe in clear and plain language the nature of the personal data breach and contain at least the information and measures referred to in points (b), (c) and (d) of Article 33(3).

3. The communication to the data subject referred to in paragraph 1 shall not be required if any of the following conditions are met:

(a) the controller has implemented appropriate technical and organisational protection measures, and those measures were applied to the personal data affected by the personal data breach, in particular those that render the personal data unintelligible to any person who is not authorised to access it, such as encryption;

(b) the controller has taken subsequent measures which ensure that the high risk to the rights and freedoms of data subjects referred to in paragraph 1 is no longer likely to materialise;

(c) it would involve disproportionate effort. In such a case, there shall instead be a public communication or similar measure whereby the data subjects are informed in an equally effective manner.

4. If the controller has not already communicated the personal data breach to the data subject, the supervisory authority, having considered the likelihood of the personal data breach resulting in a high risk, may require it to do so or may decide that any of the conditions referred to in paragraph 3 are met.

Section 3

Data protection impact assessment and prior consultation

Article 35

Data protection impact assessment

1. Where a type of processing in particular using new technologies, and taking into account the nature, scope, context and purposes of the processing, is likely to result in a high risk to the rights and freedoms of natural persons, the controller shall, prior to the processing, carry out an assessment of the impact of the envisaged processing operations on the protection of personal data. A single assessment may address a set of similar processing operations that present similar high risks.

2. The controller shall seek the advice of the data protection officer, where designated, when carrying out a data protection impact assessment.

3. A data protection impact assessment referred to in paragraph 1 shall in particular be required in the case of:

(a) a systematic and extensive evaluation of personal aspects relating to natural persons which is based on automated processing, including profiling, and on which decisions are based that produce legal effects concerning the natural person or similarly significantly affect the natural person;

(b) processing on a large scale of special categories of data referred to in Article 9(1), or of personal data relating to criminal convictions and offences referred to in Article 10; or

(c) a systematic monitoring of a publicly accessible area on a large scale.

4. The supervisory authority shall establish and make public a list of the kind of processing operations which are subject to the requirement for a data protection impact assessment pursuant to paragraph 1. The supervisory authority shall communicate those lists to the Board referred to in Article 68.

5. The supervisory authority may also establish and make public a list of the kind of processing operations for which no data protection impact assessment is required. The supervisory authority shall communicate those lists to the Board.

6. Prior to the adoption of the lists referred to in paragraphs 4 and 5, the competent supervisory authority shall apply the consistency mechanism referred to in Article 63 where such lists involve processing activities which are related to the offering of goods or services to data subjects or to the monitoring of their behaviour in several Member States, or may substantially affect the free movement of personal data within the Union.

7. The assessment shall contain at least:

(a) a systematic description of the envisaged processing operations and the purposes of the processing, including, where applicable, the legitimate interest pursued by the controller;

(b) an assessment of the necessity and proportionality of the processing operations in relation to the purposes;

(c) an assessment of the risks to the rights and freedoms of data subjects referred to in paragraph 1; and

(d) the measures envisaged to address the risks, including safeguards, security measures and mechanisms to ensure the protection of personal data and to demonstrate compliance with this Regulation taking into account the rights and legitimate interests of data subjects and other persons concerned.

8. Compliance with approved codes of conduct referred to in Article 40 by the relevant controllers or processors shall be taken into due account in assessing the impact of the processing operations performed by such controllers or processors, in particular for the purposes of a data protection impact assessment.

9. Where appropriate, the controller shall seek the views of data subjects or their representatives on the intended processing, without prejudice to the protection of commercial or public interests or the security of processing operations.

10. Where processing pursuant to point (c) or (e) of Article 6(1) has a legal basis in Union law or in the law of the Member State to which the controller is subject, that law regulates the specific processing operation or set of operations in question, and a data protection impact assessment has already been carried out as part of a general impact assessment in the context of the adoption of that legal basis, paragraphs 1 to 7 shall not apply unless Member States deem it to be necessary to carry out such an assessment prior to processing activities.

11. Where necessary, the controller shall carry out a review to assess if processing is performed in accordance with the data protection impact assessment at least when there is a change of the risk represented by processing operations.

Article 36

Prior consultation

1. The controller shall consult the supervisory authority prior to processing where a data protection impact assessment under Article 35 indicates that the processing would result in a high risk in the absence of measures taken by the controller to mitigate the risk.

2. Where the supervisory authority is of the opinion that the intended processing referred to in paragraph 1 would infringe this Regulation, in particular where the controller has insufficiently identified or mitigated the risk, the supervisory authority shall, within period of up to eight weeks of receipt of the request for consultation, provide written advice to the controller and, where applicable to the processor, and may use any of its powers referred to in Article 58. That period may be extended by six weeks, taking into account the complexity of the intended processing. The supervisory authority shall inform the controller and, where applicable, the processor, of any such extension within one month of receipt of the request for consultation together with the reasons for the delay. Those periods may be suspended until the supervisory authority has obtained information it has requested for the purposes of the consultation.

3. When consulting the supervisory authority pursuant to paragraph 1, the controller shall provide the supervisory authority with:

(a) where applicable, the respective responsibilities of the controller, joint controllers and processors involved in the processing, in particular for processing within a group of undertakings;

(b) the purposes and means of the intended processing;

(c) the measures and safeguards provided to protect the rights and freedoms of data subjects pursuant to this Regulation;

(d) where applicable, the contact details of the data protection officer;

(e) the data protection impact assessment provided for in Article 35; and

(f) any other information requested by the supervisory authority.

4. Member States shall consult the supervisory authority during the preparation of a proposal for a legislative measure to be adopted by a national parliament, or of a regulatory measure based on such a legislative measure, which relates to processing.

5. Notwithstanding paragraph 1, Member State law may require controllers to consult with, and obtain prior authorisation from, the supervisory authority in relation to processing by a controller for the performance of a task carried out by the controller in the public interest, including processing in relation to social protection and public health.

Section 4

Data protection officer

Article 37

Designation of the data protection officer

1. The controller and the processor shall designate a data protection officer in any case where:

(a) the processing is carried out by a public authority or body, except for courts acting in their judicial capacity;

(b) the core activities of the controller or the processor consist of processing operations which, by virtue of their nature, their scope and/or their purposes, require regular and systematic monitoring of data subjects on a large scale; or

(c) the core activities of the controller or the processor consist of processing on a large scale of special categories of data pursuant to Article 9 and personal data relating to criminal convictions and offences referred to in Article 10.

2. A group of undertakings may appoint a single data protection officer provided that a data protection officer is easily accessible from each establishment.

3. Where the controller or the processor is a public authority or body, a single data protection officer may be designated for several such authorities or bodies, taking account of their organisational structure and size.

4. In cases other than those referred to in paragraph 1, the controller or processor or associations and other bodies representing categories of controllers or processors may or, where required by Union or Member State law shall, designate a data protection officer. The data protection officer may act for such associations and other bodies representing controllers or processors.

5. The data protection officer shall be designated on the basis of professional qualities and, in particular, expert knowledge of data protection law and practices and the ability to fulfil the tasks referred to in Article 39.

6. The data protection officer may be a staff member of the controller or processor, or fulfil the tasks on the basis of a service contract.

7. The controller or the processor shall publish the contact details of the data protection officer and communicate them to the supervisory authority.

Article 38

Position of the data protection officer

1. The controller and the processor shall ensure that the data protection officer is involved, properly and in a timely manner, in all issues which relate to the protection of personal data.

2. The controller and processor shall support the data protection officer in performing the tasks referred to in Article 39 by providing resources necessary to carry out those tasks and access to personal data and processing operations, and to maintain his or her expert knowledge.

3. The controller and processor shall ensure that the data protection officer does not receive any instructions regarding the exercise of those tasks. He or she shall not be dismissed or penalised by the controller or the processor for performing his tasks. The data protection officer shall directly report to the highest management level of the controller or the processor.

4. Data subjects may contact the data protection officer with regard to all issues related to processing of their personal data and to the exercise of their rights under this Regulation.

5. The data protection officer shall be bound by secrecy or confidentiality concerning the performance of his or her tasks, in accordance with Union or Member State law.

6. The data protection officer may fulfil other tasks and duties. The controller or processor shall ensure that any such tasks and duties do not result in a conflict of interests.

Article 39

Tasks of the data protection officer

1. The data protection officer shall have at least the following tasks:

(a) to inform and advise the controller or the processor and the employees who carry out processing of their obligations pursuant to this Regulation and to other Union or Member State data protection provisions;

(b) to monitor compliance with this Regulation, with other Union or Member State data protection provisions and with the policies of the controller or processor in relation to the protection of personal data, including the assignment of responsibilities, awareness-raising and training of staff involved in processing operations, and the related audits;

(c) to provide advice where requested as regards the data protection impact assessment and monitor its performance pursuant to Article 35;

(d) to cooperate with the supervisory authority;

(e) to act as the contact point for the supervisory authority on issues relating to processing, including the prior consultation referred to in Article 36, and to consult, where appropriate, with regard to any other matter.

2. The data protection officer shall in the performance of his or her tasks have due regard to the risk associated with processing operations, taking into account the nature, scope, context and purposes of processing.

Section 5

Codes of conduct and certification

Article 40

Codes of conduct

1. The Member States, the supervisory authorities, the Board and the Commission shall encourage the drawing up of codes of conduct intended to contribute to the proper application of this Regulation, taking account of the specific features of the various processing sectors and the specific needs of micro, small and medium-sized enterprises.

2. Associations and other bodies representing categories of controllers or processors may prepare codes of conduct, or amend or extend such codes, for the purpose of specifying the application of this Regulation, such as with regard to:

(a) fair and transparent processing;

(b) the legitimate interests pursued by controllers in specific contexts;

(c) the collection of personal data;

(d) the pseudonymisation of personal data;

(e) the information provided to the public and to data subjects;

(f) the exercise of the rights of data subjects;

(g) the information provided to, and the protection of, children, and the manner in which the consent of the holders of parental responsibility over children is to be obtained;

(h) the measures and procedures referred to in Articles 24 and 25 and the measures to ensure security of processing referred to in Article 32;

(i) the notification of personal data breaches to supervisory authorities and the communication of such personal data breaches to data subjects;

(j) the transfer of personal data to third countries or international organisations; or

(k) out-of-court proceedings and other dispute resolution procedures for resolving disputes between controllers and data subjects with regard to processing, without prejudice to the rights of data subjects pursuant to Articles 77 and 79.

3. In addition to adherence by controllers or processors subject to this Regulation, codes of conduct approved pursuant to paragraph 5 of this Article and having general validity pursuant to paragraph 9 of this Article may also be adhered to by controllers or processors that are not subject to this Regulation pursuant to Article 3 in order to provide appropriate safeguards within the framework of personal data transfers to third countries or international organisations under the terms referred to in point (e) of Article 46(2). Such controllers or processors shall make binding and enforceable commitments, via contractual or other legally binding instruments, to apply those appropriate safeguards including with regard to the rights of data subjects.

4. A code of conduct referred to in paragraph 2 of this Article shall contain mechanisms which enable the body referred to in Article 41(1) to carry out the mandatory monitoring of compliance with its provisions by the controllers or processors which undertake to apply it, without prejudice to the tasks and powers of supervisory authorities competent pursuant to Article 55 or 56.

5. Associations and other bodies referred to in paragraph 2 of this Article which intend to prepare a code of conduct or to amend or extend an existing code shall submit the draft code, amendment or extension to the supervisory authority which is competent pursuant to Article 55. The supervisory authority shall provide an opinion on whether the draft code, amendment or extension complies with this Regulation and shall approve that draft code, amendment or extension if it finds that it provides sufficient appropriate safeguards.

6. Where the draft code, or amendment or extension is approved in accordance with paragraph 5, and where the code of conduct concerned does not relate to processing activities in several Member States, the supervisory authority shall register and publish the code.

7. Where a draft code of conduct relates to processing activities in several Member States, the supervisory authority which is competent pursuant to Article 55 shall, before approving the draft code, amendment or extension, submit it in the procedure referred to in Article 63 to the Board which shall provide an opinion on whether the draft code, amendment or extension complies with this Regulation or, in the situation referred to in paragraph 3 of this Article, provides appropriate safeguards.

8. Where the opinion referred to in paragraph 7 confirms that the draft code, amendment or extension complies with this Regulation, or, in the situation referred to in paragraph 3, provides appropriate safeguards, the Board shall submit its opinion to the Commission.

9. The Commission may, by way of implementing acts, decide that the approved code of conduct, amendment or extension submitted to it pursuant to paragraph 8 of this Article have general validity within the Union. Those implementing acts shall be adopted in accordance with the examination procedure set out in Article 93(2).

10. The Commission shall ensure appropriate publicity for the approved codes which have been decided as having general validity in accordance with paragraph 9.

11. The Board shall collate all approved codes of conduct, amendments and extensions in a register and shall make them publicly available by way of appropriate means.

Article 41

Monitoring of approved codes of conduct

1. Without prejudice to the tasks and powers of the competent supervisory authority under Articles 57 and 58, the monitoring of compliance with a code of conduct pursuant to Article 40 may be carried out by a body which has an appropriate level of expertise in relation to the subject-matter of the code and is accredited for that purpose by the competent supervisory authority.

2. A body as referred to in paragraph 1 may be accredited to monitor compliance with a code of conduct where that body has:

(a) demonstrated its independence and expertise in relation to the subject-matter of the code to the satisfaction of the competent supervisory authority;

(b) established procedures which allow it to assess the eligibility of controllers and processors concerned to apply the code, to monitor their compliance with its provisions and to periodically review its operation;

(c) established procedures and structures to handle complaints about infringements of the code or the manner in which the code has been, or is being, implemented by a controller or processor, and to make those procedures and structures transparent to data subjects and the public; and

(d) demonstrated to the satisfaction of the competent supervisory authority that its tasks and duties do not result in a conflict of interests.

3. The competent supervisory authority shall submit the draft criteria for accreditation of a body as referred to in paragraph 1 of this Article to the Board pursuant to the consistency mechanism referred to in Article 63.

4. Without prejudice to the tasks and powers of the competent supervisory authority and the provisions of Chapter VIII, a body as referred to in paragraph 1 of this Article shall, subject to appropriate safeguards, take appropriate action in cases of infringement of the code by a controller or processor, including suspension or exclusion of the controller or processor concerned from the code. It shall inform the competent supervisory authority of such actions and the reasons for taking them.

5. The competent supervisory authority shall revoke the accreditation of a body as referred to in paragraph 1 if the conditions for accreditation are not, or are no longer, met or where actions taken by the body infringe this Regulation.

6. This Article shall not apply to processing carried out by public authorities and bodies.

Article 42

Certification

1. The Member States, the supervisory authorities, the Board and the Commission shall encourage, in particular at Union level, the establishment of data protection certification mechanisms and of data protection seals and marks, for the purpose of demonstrating compliance with this Regulation of processing operations by controllers and processors. The specific needs of micro, small and medium-sized enterprises shall be taken into account.

2. In addition to adherence by controllers or processors subject to this Regulation, data protection certification mechanisms, seals or marks approved pursuant to paragraph 5 of this Article may be established for the purpose of demonstrating the existence of appropriate safeguards provided by controllers or processors that are not subject to this Regulation pursuant to Article 3 within the framework of personal data transfers to third countries or international organisations under the terms referred to in point (f) of Article 46(2). Such controllers or processors shall make binding and enforceable commitments, via contractual or other legally binding instruments, to apply those appropriate safeguards, including with regard to the rights of data subjects.

3. The certification shall be voluntary and available via a process that is transparent.

4. A certification pursuant to this Article does not reduce the responsibility of the controller or the processor for compliance with this Regulation and is without prejudice to the tasks and powers of the supervisory authorities which are competent pursuant to Article 55 or 56.

5. A certification pursuant to this Article shall be issued by the certification bodies referred to in Article 43 or by the competent supervisory authority, on the basis of criteria approved by that competent supervisory authority pursuant to Article 58(3) or by the Board pursuant to Article 63. Where the criteria are approved by the Board, this may result in a common certification, the European Data Protection Seal.

6. The controller or processor which submits its processing to the certification mechanism shall provide the certification body referred to in Article 43, or where applicable, the competent supervisory authority, with all information and access to its processing activities which are necessary to conduct the certification procedure.

7. Certification shall be issued to a controller or processor for a maximum period of three years and may be renewed, under the same conditions, provided that the relevant requirements continue to be met. Certification shall be withdrawn, as applicable, by the certification bodies referred to in Article 43 or by the competent supervisory authority where the requirements for the certification are not or are no longer met.

8. The Board shall collate all certification mechanisms and data protection seals and marks in a register and shall make them publicly available by any appropriate means.

Article 43

Certification bodies

1. Without prejudice to the tasks and powers of the competent supervisory authority under Articles 57 and 58, certification bodies which have an appropriate level of expertise in relation to data protection shall, after informing the supervisory authority in order to allow it to exercise its powers pursuant to point (h) of Article 58(2) where necessary, issue and renew certification. Member States shall ensure that those certification bodies are accredited by one or both of the following:

(a) the supervisory authority which is competent pursuant to Article 55 or 56;

(b) the national accreditation body named in accordance with Regulation (EC) No 765/2008 of the European Parliament and of the Council ([1]) in accordance with EN-ISO/IEC 17065/2012 and with the additional requirements established by the supervisory authority which is competent pursuant to Article 55 or 56.

2. Certification bodies referred to in paragraph 1 shall be accredited in accordance with that paragraph only where they have:

(a) demonstrated their independence and expertise in relation to the subject-matter of the certification to the satisfaction of the competent supervisory authority;

([1]) Regulation (EC) No 765/2008 of the European Parliament and of the Council of 9 July 2008 setting out the requirements for accreditation and market surveillance relating to the marketing of products and repealing Regulation (EEC) No 339/93 (OJ L 218, 13.8.2008, p. 30).

(b) undertaken to respect the criteria referred to in Article 42(5) and approved by the supervisory authority which is competent pursuant to Article 55 or 56 or by the Board pursuant to Article 63;

(c) established procedures for the issuing, periodic review and withdrawal of data protection certification, seals and marks;

(d) established procedures and structures to handle complaints about infringements of the certification or the manner in which the certification has been, or is being, implemented by the controller or processor, and to make those procedures and structures transparent to data subjects and the public; and

(e) demonstrated, to the satisfaction of the competent supervisory authority, that their tasks and duties do not result in a conflict of interests.

3. The accreditation of certification bodies as referred to in paragraphs 1 and 2 of this Article shall take place on the basis of criteria approved by the supervisory authority which is competent pursuant to Article 55 or 56 or by the Board pursuant to Article 63. In the case of accreditation pursuant to point (b) of paragraph 1 of this Article, those requirements shall complement those envisaged in Regulation (EC) No 765/2008 and the technical rules that describe the methods and procedures of the certification bodies.

4. The certification bodies referred to in paragraph 1 shall be responsible for the proper assessment leading to the certification or the withdrawal of such certification without prejudice to the responsibility of the controller or processor for compliance with this Regulation. The accreditation shall be issued for a maximum period of five years and may be renewed on the same conditions provided that the certification body meets the requirements set out in this Article.

5. The certification bodies referred to in paragraph 1 shall provide the competent supervisory authorities with the reasons for granting or withdrawing the requested certification.

6. The requirements referred to in paragraph 3 of this Article and the criteria referred to in Article 42(5) shall be made public by the supervisory authority in an easily accessible form. The supervisory authorities shall also transmit those requirements and criteria to the Board. The Board shall collate all certification mechanisms and data protection seals in a register and shall make them publicly available by any appropriate means.

7. Without prejudice to Chapter VIII, the competent supervisory authority or the national accreditation body shall revoke an accreditation of a certification body pursuant to paragraph 1 of this Article where the conditions for the accreditation are not, or are no longer, met or where actions taken by a certification body infringe this Regulation.

8. The Commission shall be empowered to adopt delegated acts in accordance with Article 92 for the purpose of specifying the requirements to be taken into account for the data protection certification mechanisms referred to in Article 42(1).

9. The Commission may adopt implementing acts laying down technical standards for certification mechanisms and data protection seals and marks, and mechanisms to promote and recognise those certification mechanisms, seals and marks. Those implementing acts shall be adopted in accordance with the examination procedure referred to in Article 93(2).

CHAPTER V

Transfers of personal data to third countries or international organisations

Article 44

General principle for transfers

Any transfer of personal data which are undergoing processing or are intended for processing after transfer to a third country or to an international organisation shall take place only if, subject to the other provisions of this Regulation, the conditions laid down in this Chapter are complied with by the controller and processor, including for onward transfers of personal data from the third country or an international organisation to another third country or to another international organisation. All provisions in this Chapter shall be applied in order to ensure that the level of protection of natural persons guaranteed by this Regulation is not undermined.

Article 45

Transfers on the basis of an adequacy decision

1. A transfer of personal data to a third country or an international organisation may take place where the Commission has decided that the third country, a territory or one or more specified sectors within that third country, or the international organisation in question ensures an adequate level of protection. Such a transfer shall not require any specific authorisation.

2. When assessing the adequacy of the level of protection, the Commission shall, in particular, take account of the following elements:

(a) the rule of law, respect for human rights and fundamental freedoms, relevant legislation, both general and sectoral, including concerning public security, defence, national security and criminal law and the access of public authorities to personal data, as well as the implementation of such legislation, data protection rules, professional rules and security measures, including rules for the onward transfer of personal data to another third country or international organisation which are complied with in that country or international organisation, case-law, as well as effective and enforceable data subject rights and effective administrative and judicial redress for the data subjects whose personal data are being transferred;

(b) the existence and effective functioning of one or more independent supervisory authorities in the third country or to which an international organisation is subject, with responsibility for ensuring and enforcing compliance with the data protection rules, including adequate enforcement powers, for assisting and advising the data subjects in exercising their rights and for cooperation with the supervisory authorities of the Member States; and

(c) the international commitments the third country or international organisation concerned has entered into, or other obligations arising from legally binding conventions or instruments as well as from its participation in multilateral or regional systems, in particular in relation to the protection of personal data.

3. The Commission, after assessing the adequacy of the level of protection, may decide, by means of implementing act, that a third country, a territory or one or more specified sectors within a third country, or an international organisation ensures an adequate level of protection within the meaning of paragraph 2 of this Article. The implementing act shall provide for a mechanism for a periodic review, at least every four years, which shall take into account all relevant developments in the third country or international organisation. The implementing act shall specify its territorial and sectoral application and, where applicable, identify the supervisory authority or authorities referred to in point (b) of paragraph 2 of this Article. The implementing act shall be adopted in accordance with the examination procedure referred to in Article 93(2).

4. The Commission shall, on an ongoing basis, monitor developments in third countries and international organisations that could affect the functioning of decisions adopted pursuant to paragraph 3 of this Article and decisions adopted on the basis of Article 25(6) of Directive 95/46/EC.

5. The Commission shall, where available information reveals, in particular following the review referred to in paragraph 3 of this Article, that a third country, a territory or one or more specified sectors within a third country, or an international organisation no longer ensures an adequate level of protection within the meaning of paragraph 2 of this Article, to the extent necessary, repeal, amend or suspend the decision referred to in paragraph 3 of this Article by means of implementing acts without retro-active effect. Those implementing acts shall be adopted in accordance with the examination procedure referred to in Article 93(2).

On duly justified imperative grounds of urgency, the Commission shall adopt immediately applicable implementing acts in accordance with the procedure referred to in Article 93(3).

6. The Commission shall enter into consultations with the third country or international organisation with a view to remedying the situation giving rise to the decision made pursuant to paragraph 5.

7. A decision pursuant to paragraph 5 of this Article is without prejudice to transfers of personal data to the third country, a territory or one or more specified sectors within that third country, or the international organisation in question pursuant to Articles 46 to 49.

8. The Commission shall publish in the *Official Journal of the European Union* and on its website a list of the third countries, territories and specified sectors within a third country and international organisations for which it has decided that an adequate level of protection is or is no longer ensured.

9. Decisions adopted by the Commission on the basis of Article 25(6) of Directive 95/46/EC shall remain in force until amended, replaced or repealed by a Commission Decision adopted in accordance with paragraph 3 or 5 of this Article.

Article 46

Transfers subject to appropriate safeguards

1. In the absence of a decision pursuant to Article 45(3), a controller or processor may transfer personal data to a third country or an international organisation only if the controller or processor has provided appropriate safeguards, and on condition that enforceable data subject rights and effective legal remedies for data subjects are available.

2. The appropriate safeguards referred to in paragraph 1 may be provided for, without requiring any specific authorisation from a supervisory authority, by:

(a) a legally binding and enforceable instrument between public authorities or bodies;

(b) binding corporate rules in accordance with Article 47;

(c) standard data protection clauses adopted by the Commission in accordance with the examination procedure referred to in Article 93(2);

(d) standard data protection clauses adopted by a supervisory authority and approved by the Commission pursuant to the examination procedure referred to in Article 93(2);

(e) an approved code of conduct pursuant to Article 40 together with binding and enforceable commitments of the controller or processor in the third country to apply the appropriate safeguards, including as regards data subjects' rights; or

(f) an approved certification mechanism pursuant to Article 42 together with binding and enforceable commitments of the controller or processor in the third country to apply the appropriate safeguards, including as regards data subjects' rights.

3. Subject to the authorisation from the competent supervisory authority, the appropriate safeguards referred to in paragraph 1 may also be provided for, in particular, by:

(a) contractual clauses between the controller or processor and the controller, processor or the recipient of the personal data in the third country or international organisation; or

(b) provisions to be inserted into administrative arrangements between public authorities or bodies which include enforceable and effective data subject rights.

4. The supervisory authority shall apply the consistency mechanism referred to in Article 63 in the cases referred to in paragraph 3 of this Article.

5. Authorisations by a Member State or supervisory authority on the basis of Article 26(2) of Directive 95/46/EC shall remain valid until amended, replaced or repealed, if necessary, by that supervisory authority. Decisions adopted by the Commission on the basis of Article 26(4) of Directive 95/46/EC shall remain in force until amended, replaced or repealed, if necessary, by a Commission Decision adopted in accordance with paragraph 2 of this Article.

Article 47

Binding corporate rules

1. The competent supervisory authority shall approve binding corporate rules in accordance with the consistency mechanism set out in Article 63, provided that they:

(a) are legally binding and apply to and are enforced by every member concerned of the group of undertakings, or group of enterprises engaged in a joint economic activity, including their employees;

(b) expressly confer enforceable rights on data subjects with regard to the processing of their personal data; and

(c) fulfil the requirements laid down in paragraph 2.

2. The binding corporate rules referred to in paragraph 1 shall specify at least:

(a) the structure and contact details of the group of undertakings, or group of enterprises engaged in a joint economic activity and of each of its members;

(b) the data transfers or set of transfers, including the categories of personal data, the type of processing and its purposes, the type of data subjects affected and the identification of the third country or countries in question;

(c) their legally binding nature, both internally and externally;

(d) the application of the general data protection principles, in particular purpose limitation, data minimisation, limited storage periods, data quality, data protection by design and by default, legal basis for processing, processing of special categories of personal data, measures to ensure data security, and the requirements in respect of onward transfers to bodies not bound by the binding corporate rules;

(e) the rights of data subjects in regard to processing and the means to exercise those rights, including the right not to be subject to decisions based solely on automated processing, including profiling in accordance with Article 22, the right to lodge a complaint with the competent supervisory authority and before the competent courts of the Member States in accordance with Article 79, and to obtain redress and, where appropriate, compensation for a breach of the binding corporate rules;

(f) the acceptance by the controller or processor established on the territory of a Member State of liability for any breaches of the binding corporate rules by any member concerned not established in the Union; the controller or the processor shall be exempt from that liability, in whole or in part, only if it proves that that member is not responsible for the event giving rise to the damage;

(g) how the information on the binding corporate rules, in particular on the provisions referred to in points (d), (e) and (f) of this paragraph is provided to the data subjects in addition to Articles 13 and 14;

(h) the tasks of any data protection officer designated in accordance with Article 37 or any other person or entity in charge of the monitoring compliance with the binding corporate rules within the group of undertakings, or group of enterprises engaged in a joint economic activity, as well as monitoring training and complaint-handling;

(i) the complaint procedures;

(j) the mechanisms within the group of undertakings, or group of enterprises engaged in a joint economic activity for ensuring the verification of compliance with the binding corporate rules. Such mechanisms shall include data protection audits and methods for ensuring corrective actions to protect the rights of the data subject. Results of such verification should be communicated to the person or entity referred to in point (h) and to the board of the controlling undertaking of a group of undertakings, or of the group of enterprises engaged in a joint economic activity, and should be available upon request to the competent supervisory authority;

(k) the mechanisms for reporting and recording changes to the rules and reporting those changes to the supervisory authority;

(l) the cooperation mechanism with the supervisory authority to ensure compliance by any member of the group of undertakings, or group of enterprises engaged in a joint economic activity, in particular by making available to the supervisory authority the results of verifications of the measures referred to in point (j);

(m) the mechanisms for reporting to the competent supervisory authority any legal requirements to which a member of the group of undertakings, or group of enterprises engaged in a joint economic activity is subject in a third country which are likely to have a substantial adverse effect on the guarantees provided by the binding corporate rules; and

(n) the appropriate data protection training to personnel having permanent or regular access to personal data.

3. The Commission may specify the format and procedures for the exchange of information between controllers, processors and supervisory authorities for binding corporate rules within the meaning of this Article. Those implementing acts shall be adopted in accordance with the examination procedure set out in Article 93(2).

Article 48

Transfers or disclosures not authorised by Union law

Any judgment of a court or tribunal and any decision of an administrative authority of a third country requiring a controller or processor to transfer or disclose personal data may only be recognised or enforceable in any manner if based on an international agreement, such as a mutual legal assistance treaty, in force between the requesting third country and the Union or a Member State, without prejudice to other grounds for transfer pursuant to this Chapter.

Article 49

Derogations for specific situations

1. In the absence of an adequacy decision pursuant to Article 45(3), or of appropriate safeguards pursuant to Article 46, including binding corporate rules, a transfer or a set of transfers of personal data to a third country or an international organisation shall take place only on one of the following conditions:

(a) the data subject has explicitly consented to the proposed transfer, after having been informed of the possible risks of such transfers for the data subject due to the absence of an adequacy decision and appropriate safeguards;

(b) the transfer is necessary for the performance of a contract between the data subject and the controller or the implementation of pre-contractual measures taken at the data subject's request;

(c) the transfer is necessary for the conclusion or performance of a contract concluded in the interest of the data subject between the controller and another natural or legal person;

(d) the transfer is necessary for important reasons of public interest;

(e) the transfer is necessary for the establishment, exercise or defence of legal claims;

(f) the transfer is necessary in order to protect the vital interests of the data subject or of other persons, where the data subject is physically or legally incapable of giving consent;

(g) the transfer is made from a register which according to Union or Member State law is intended to provide information to the public and which is open to consultation either by the public in general or by any person who can demonstrate a legitimate interest, but only to the extent that the conditions laid down by Union or Member State law for consultation are fulfilled in the particular case.

Where a transfer could not be based on a provision in Article 45 or 46, including the provisions on binding corporate rules, and none of the derogations for a specific situation referred to in the first subparagraph of this paragraph is applicable, a transfer to a third country or an international organisation may take place only if the transfer is not repetitive, concerns only a limited number of data subjects, is necessary for the purposes of compelling legitimate interests pursued by the controller which are not overridden by the interests or rights and freedoms of the data subject, and the controller has assessed all the circumstances surrounding the data transfer and has on the basis of that assessment provided suitable safeguards with regard to the protection of personal data. The controller shall inform the supervisory authority of the transfer. The controller shall, in addition to providing the information referred to in Articles 13 and 14, inform the data subject of the transfer and on the compelling legitimate interests pursued.

2. A transfer pursuant to point (g) of the first subparagraph of paragraph 1 shall not involve the entirety of the personal data or entire categories of the personal data contained in the register. Where the register is intended for consultation by persons having a legitimate interest, the transfer shall be made only at the request of those persons or if they are to be the recipients.

3. Points (a), (b) and (c) of the first subparagraph of paragraph 1 and the second subparagraph thereof shall not apply to activities carried out by public authorities in the exercise of their public powers.

4. The public interest referred to in point (d) of the first subparagraph of paragraph 1 shall be recognised in Union law or in the law of the Member State to which the controller is subject.

5. In the absence of an adequacy decision, Union or Member State law may, for important reasons of public interest, expressly set limits to the transfer of specific categories of personal data to a third country or an international organisation. Member States shall notify such provisions to the Commission.

6. The controller or processor shall document the assessment as well as the suitable safeguards referred to in the second subparagraph of paragraph 1 of this Article in the records referred to in Article 30.

Article 50

International cooperation for the protection of personal data

In relation to third countries and international organisations, the Commission and supervisory authorities shall take appropriate steps to:

(a) develop international cooperation mechanisms to facilitate the effective enforcement of legislation for the protection of personal data;

(b) provide international mutual assistance in the enforcement of legislation for the protection of personal data, including through notification, complaint referral, investigative assistance and information exchange, subject to appropriate safeguards for the protection of personal data and other fundamental rights and freedoms;

(c) engage relevant stakeholders in discussion and activities aimed at furthering international cooperation in the enforcement of legislation for the protection of personal data;

(d) promote the exchange and documentation of personal data protection legislation and practice, including on jurisdictional conflicts with third countries.

CHAPTER VI

Independent supervisory authorities

Section 1

Independent status

Article 51

Supervisory authority

1. Each Member State shall provide for one or more independent public authorities to be responsible for monitoring the application of this Regulation, in order to protect the fundamental rights and freedoms of natural persons in relation to processing and to facilitate the free flow of personal data within the Union ('supervisory authority').

2. Each supervisory authority shall contribute to the consistent application of this Regulation throughout the Union. For that purpose, the supervisory authorities shall cooperate with each other and the Commission in accordance with Chapter VII.

3. Where more than one supervisory authority is established in a Member State, that Member State shall designate the supervisory authority which is to represent those authorities in the Board and shall set out the mechanism to ensure compliance by the other authorities with the rules relating to the consistency mechanism referred to in Article 63.

4. Each Member State shall notify to the Commission the provisions of its law which it adopts pursuant to this Chapter, by 25 May 2018 and, without delay, any subsequent amendment affecting them.

Article 52

Independence

1. Each supervisory authority shall act with complete independence in performing its tasks and exercising its powers in accordance with this Regulation.

2. The member or members of each supervisory authority shall, in the performance of their tasks and exercise of their powers in accordance with this Regulation, remain free from external influence, whether direct or indirect, and shall neither seek nor take instructions from anybody.

3. Member or members of each supervisory authority shall refrain from any action incompatible with their duties and shall not, during their term of office, engage in any incompatible occupation, whether gainful or not.

4. Each Member State shall ensure that each supervisory authority is provided with the human, technical and financial resources, premises and infrastructure necessary for the effective performance of its tasks and exercise of its powers, including those to be carried out in the context of mutual assistance, cooperation and participation in the Board.

5. Each Member State shall ensure that each supervisory authority chooses and has its own staff which shall be subject to the exclusive direction of the member or members of the supervisory authority concerned.

6. Each Member State shall ensure that each supervisory authority is subject to financial control which does not affect its independence and that it has separate, public annual budgets, which may be part of the overall state or national budget.

Article 53

General conditions for the members of the supervisory authority

1. Member States shall provide for each member of their supervisory authorities to be appointed by means of a transparent procedure by:

— their parliament;

— their government;

— their head of State; or

— an independent body entrusted with the appointment under Member State law.

2. Each member shall have the qualifications, experience and skills, in particular in the area of the protection of personal data, required to perform its duties and exercise its powers.

3. The duties of a member shall end in the event of the expiry of the term of office, resignation or compulsory retirement, in accordance with the law of the Member State concerned.

4. A member shall be dismissed only in cases of serious misconduct or if the member no longer fulfils the conditions required for the performance of the duties.

Article 54

Rules on the establishment of the supervisory authority

1. Each Member State shall provide by law for all of the following:

(a) the establishment of each supervisory authority;

(b) the qualifications and eligibility conditions required to be appointed as member of each supervisory authority;

(c) the rules and procedures for the appointment of the member or members of each supervisory authority;

(d) the duration of the term of the member or members of each supervisory authority of no less than four years, except for the first appointment after 24 May 2016, part of which may take place for a shorter period where that is necessary to protect the independence of the supervisory authority by means of a staggered appointment procedure;

(e) whether and, if so, for how many terms the member or members of each supervisory authority is eligible for reappointment;

(f) the conditions governing the obligations of the member or members and staff of each supervisory authority, prohibitions on actions, occupations and benefits incompatible therewith during and after the term of office and rules governing the cessation of employment.

2. The member or members and the staff of each supervisory authority shall, in accordance with Union or Member State law, be subject to a duty of professional secrecy both during and after their term of office, with regard to any confidential information which has come to their knowledge in the course of the performance of their tasks or exercise of their powers. During their term of office, that duty of professional secrecy shall in particular apply to reporting by natural persons of infringements of this Regulation.

Section 2

Competence, tasks and powers

Article 55

Competence

1. Each supervisory authority shall be competent for the performance of the tasks assigned to and the exercise of the powers conferred on it in accordance with this Regulation on the territory of its own Member State.

2. Where processing is carried out by public authorities or private bodies acting on the basis of point (c) or (e) of Article 6(1), the supervisory authority of the Member State concerned shall be competent. In such cases Article 56 does not apply.

3. Supervisory authorities shall not be competent to supervise processing operations of courts acting in their judicial capacity.

Article 56

Competence of the lead supervisory authority

1. Without prejudice to Article 55, the supervisory authority of the main establishment or of the single establishment of the controller or processor shall be competent to act as lead supervisory authority for the cross-border processing carried out by that controller or processor in accordance with the procedure provided in Article 60.

2. By derogation from paragraph 1, each supervisory authority shall be competent to handle a complaint lodged with it or a possible infringement of this Regulation, if the subject matter relates only to an establishment in its Member State or substantially affects data subjects only in its Member State.

3. In the cases referred to in paragraph 2 of this Article, the supervisory authority shall inform the lead supervisory authority without delay on that matter. Within a period of three weeks after being informed the lead supervisory authority shall decide whether or not it will handle the case in accordance with the procedure provided in Article 60, taking into account whether or not there is an establishment of the controller or processor in the Member State of which the supervisory authority informed it.

4. Where the lead supervisory authority decides to handle the case, the procedure provided in Article 60 shall apply. The supervisory authority which informed the lead supervisory authority may submit to the lead supervisory authority a draft for a decision. The lead supervisory authority shall take utmost account of that draft when preparing the draft decision referred to in Article 60(3).

5. Where the lead supervisory authority decides not to handle the case, the supervisory authority which informed the lead supervisory authority shall handle it according to Articles 61 and 62.

6. The lead supervisory authority shall be the sole interlocutor of the controller or processor for the cross-border processing carried out by that controller or processor.

Article 57

Tasks

1. Without prejudice to other tasks set out under this Regulation, each supervisory authority shall on its territory:

(a) monitor and enforce the application of this Regulation;

(b) promote public awareness and understanding of the risks, rules, safeguards and rights in relation to processing. Activities addressed specifically to children shall receive specific attention;

(c) advise, in accordance with Member State law, the national parliament, the government, and other institutions and bodies on legislative and administrative measures relating to the protection of natural persons' rights and freedoms with regard to processing;

(d) promote the awareness of controllers and processors of their obligations under this Regulation;

(e) upon request, provide information to any data subject concerning the exercise of their rights under this Regulation and, if appropriate, cooperate with the supervisory authorities in other Member States to that end;

(f) handle complaints lodged by a data subject, or by a body, organisation or association in accordance with Article 80, and investigate, to the extent appropriate, the subject matter of the complaint and inform the complainant of the progress and the outcome of the investigation within a reasonable period, in particular if further investigation or coordination with another supervisory authority is necessary;

(g) cooperate with, including sharing information and provide mutual assistance to, other supervisory authorities with a view to ensuring the consistency of application and enforcement of this Regulation;

(h) conduct investigations on the application of this Regulation, including on the basis of information received from another supervisory authority or other public authority;

(i) monitor relevant developments, insofar as they have an impact on the protection of personal data, in particular the development of information and communication technologies and commercial practices;

(j) adopt standard contractual clauses referred to in Article 28(8) and in point (d) of Article 46(2);

(k) establish and maintain a list in relation to the requirement for data protection impact assessment pursuant to Article 35(4);

(l) give advice on the processing operations referred to in Article 36(2);

(m) encourage the drawing up of codes of conduct pursuant to Article 40(1) and provide an opinion and approve such codes of conduct which provide sufficient safeguards, pursuant to Article 40(5);

(n) encourage the establishment of data protection certification mechanisms and of data protection seals and marks pursuant to Article 42(1), and approve the criteria of certification pursuant to Article 42(5);

(o) where applicable, carry out a periodic review of certifications issued in accordance with Article 42(7);

(p) draft and publish the criteria for accreditation of a body for monitoring codes of conduct pursuant to Article 41 and of a certification body pursuant to Article 43;

(q) conduct the accreditation of a body for monitoring codes of conduct pursuant to Article 41 and of a certification body pursuant to Article 43;

(r) authorise contractual clauses and provisions referred to in Article 46(3);

(s) approve binding corporate rules pursuant to Article 47;

(t) contribute to the activities of the Board;

(u) keep internal records of infringements of this Regulation and of measures taken in accordance with Article 58(2); and

(v) fulfil any other tasks related to the protection of personal data.

2. Each supervisory authority shall facilitate the submission of complaints referred to in point (f) of paragraph 1 by measures such as a complaint submission form which can also be completed electronically, without excluding other means of communication.

3. The performance of the tasks of each supervisory authority shall be free of charge for the data subject and, where applicable, for the data protection officer.

4. Where requests are manifestly unfounded or excessive, in particular because of their repetitive character, the supervisory authority may charge a reasonable fee based on administrative costs, or refuse to act on the request. The supervisory authority shall bear the burden of demonstrating the manifestly unfounded or excessive character of the request.

Article 58

Powers

1. Each supervisory authority shall have all of the following investigative powers:

(a) to order the controller and the processor, and, where applicable, the controller's or the processor's representative to provide any information it requires for the performance of its tasks;

(b) to carry out investigations in the form of data protection audits;

(c) to carry out a review on certifications issued pursuant to Article 42(7);

(d) to notify the controller or the processor of an alleged infringement of this Regulation;

(e) to obtain, from the controller and the processor, access to all personal data and to all information necessary for the performance of its tasks;

(f) to obtain access to any premises of the controller and the processor, including to any data processing equipment and means, in accordance with Union or Member State procedural law.

2. Each supervisory authority shall have all of the following corrective powers:

(a) to issue warnings to a controller or processor that intended processing operations are likely to infringe provisions of this Regulation;

(b) to issue reprimands to a controller or a processor where processing operations have infringed provisions of this Regulation;

(c) to order the controller or the processor to comply with the data subject's requests to exercise his or her rights pursuant to this Regulation;

(d) to order the controller or processor to bring processing operations into compliance with the provisions of this Regulation, where appropriate, in a specified manner and within a specified period;

(e) to order the controller to communicate a personal data breach to the data subject;

(f) to impose a temporary or definitive limitation including a ban on processing;

(g) to order the rectification or erasure of personal data or restriction of processing pursuant to Articles 16, 17 and 18 and the notification of such actions to recipients to whom the personal data have been disclosed pursuant to Article 17(2) and Article 19;

(h) to withdraw a certification or to order the certification body to withdraw a certification issued pursuant to Articles 42 and 43, or to order the certification body not to issue certification if the requirements for the certification are not or are no longer met;

(i) to impose an administrative fine pursuant to Article 83, in addition to, or instead of measures referred to in this paragraph, depending on the circumstances of each individual case;

(j) to order the suspension of data flows to a recipient in a third country or to an international organisation.

3. Each supervisory authority shall have all of the following authorisation and advisory powers:

(a) to advise the controller in accordance with the prior consultation procedure referred to in Article 36;

(b) to issue, on its own initiative or on request, opinions to the national parliament, the Member State government or, in accordance with Member State law, to other institutions and bodies as well as to the public on any issue related to the protection of personal data;

(c) to authorise processing referred to in Article 36(5), if the law of the Member State requires such prior authorisation;

(d) to issue an opinion and approve draft codes of conduct pursuant to Article 40(5);

(e) to accredit certification bodies pursuant to Article 43;

(f) to issue certifications and approve criteria of certification in accordance with Article 42(5);

(g) to adopt standard data protection clauses referred to in Article 28(8) and in point (d) of Article 46(2);

(h) to authorise contractual clauses referred to in point (a) of Article 46(3);

(i) to authorise administrative arrangements referred to in point (b) of Article 46(3);

(j) to approve binding corporate rules pursuant to Article 47.

4. The exercise of the powers conferred on the supervisory authority pursuant to this Article shall be subject to appropriate safeguards, including effective judicial remedy and due process, set out in Union and Member State law in accordance with the Charter.

5. Each Member State shall provide by law that its supervisory authority shall have the power to bring infringements of this Regulation to the attention of the judicial authorities and where appropriate, to commence or engage otherwise in legal proceedings, in order to enforce the provisions of this Regulation.

6. Each Member State may provide by law that its supervisory authority shall have additional powers to those referred to in paragraphs 1, 2 and 3. The exercise of those powers shall not impair the effective operation of Chapter VII.

Article 59

Activity reports

Each supervisory authority shall draw up an annual report on its activities, which may include a list of types of infringement notified and types of measures taken in accordance with Article 58(2). Those reports shall be transmitted to the national parliament, the government and other authorities as designated by Member State law. They shall be made available to the public, to the Commission and to the Board.

CHAPTER VII

Cooperation and consistency

Section 1

Cooperation

Article 60

Cooperation between the lead supervisory authority and the other supervisory authorities concerned

1. The lead supervisory authority shall cooperate with the other supervisory authorities concerned in accordance with this Article in an endeavour to reach consensus. The lead supervisory authority and the supervisory authorities concerned shall exchange all relevant information with each other.

2. The lead supervisory authority may request at any time other supervisory authorities concerned to provide mutual assistance pursuant to Article 61 and may conduct joint operations pursuant to Article 62, in particular for carrying out investigations or for monitoring the implementation of a measure concerning a controller or processor established in another Member State.

3. The lead supervisory authority shall, without delay, communicate the relevant information on the matter to the other supervisory authorities concerned. It shall without delay submit a draft decision to the other supervisory authorities concerned for their opinion and take due account of their views.

4. Where any of the other supervisory authorities concerned within a period of four weeks after having been consulted in accordance with paragraph 3 of this Article, expresses a relevant and reasoned objection to the draft decision, the lead supervisory authority shall, if it does not follow the relevant and reasoned objection or is of the opinion that the objection is not relevant or reasoned, submit the matter to the consistency mechanism referred to in Article 63.

5. Where the lead supervisory authority intends to follow the relevant and reasoned objection made, it shall submit to the other supervisory authorities concerned a revised draft decision for their opinion. That revised draft decision shall be subject to the procedure referred to in paragraph 4 within a period of two weeks.

6. Where none of the other supervisory authorities concerned has objected to the draft decision submitted by the lead supervisory authority within the period referred to in paragraphs 4 and 5, the lead supervisory authority and the supervisory authorities concerned shall be deemed to be in agreement with that draft decision and shall be bound by it.

7. The lead supervisory authority shall adopt and notify the decision to the main establishment or single establishment of the controller or processor, as the case may be and inform the other supervisory authorities concerned and the Board of the decision in question, including a summary of the relevant facts and grounds. The supervisory authority with which a complaint has been lodged shall inform the complainant on the decision.

8. By derogation from paragraph 7, where a complaint is dismissed or rejected, the supervisory authority with which the complaint was lodged shall adopt the decision and notify it to the complainant and shall inform the controller thereof.

9. Where the lead supervisory authority and the supervisory authorities concerned agree to dismiss or reject parts of a complaint and to act on other parts of that complaint, a separate decision shall be adopted for each of those parts of the matter. The lead supervisory authority shall adopt the decision for the part concerning actions in relation to the controller, shall notify it to the main establishment or single establishment of the controller or processor on the territory of its Member State and shall inform the complainant thereof, while the supervisory authority of the complainant shall adopt the decision for the part concerning dismissal or rejection of that complaint, and shall notify it to that complainant and shall inform the controller or processor thereof.

10. After being notified of the decision of the lead supervisory authority pursuant to paragraphs 7 and 9, the controller or processor shall take the necessary measures to ensure compliance with the decision as regards processing activities in the context of all its establishments in the Union. The controller or processor shall notify the measures taken for complying with the decision to the lead supervisory authority, which shall inform the other supervisory authorities concerned.

11. Where, in exceptional circumstances, a supervisory authority concerned has reasons to consider that there is an urgent need to act in order to protect the interests of data subjects, the urgency procedure referred to in Article 66 shall apply.

12. The lead supervisory authority and the other supervisory authorities concerned shall supply the information required under this Article to each other by electronic means, using a standardised format.

Article 61

Mutual assistance

1. Supervisory authorities shall provide each other with relevant information and mutual assistance in order to implement and apply this Regulation in a consistent manner, and shall put in place measures for effective cooperation with one another. Mutual assistance shall cover, in particular, information requests and supervisory measures, such as requests to carry out prior authorisations and consultations, inspections and investigations.

2. Each supervisory authority shall take all appropriate measures required to reply to a request of another supervisory authority without undue delay and no later than one month after receiving the request. Such measures may include, in particular, the transmission of relevant information on the conduct of an investigation.

3. Requests for assistance shall contain all the necessary information, including the purpose of and reasons for the request. Information exchanged shall be used only for the purpose for which it was requested.

4. The requested supervisory authority shall not refuse to comply with the request unless:

(a) it is not competent for the subject-matter of the request or for the measures it is requested to execute; or

(b) compliance with the request would infringe this Regulation or Union or Member State law to which the supervisory authority receiving the request is subject.

5. The requested supervisory authority shall inform the requesting supervisory authority of the results or, as the case may be, of the progress of the measures taken in order to respond to the request. The requested supervisory authority shall provide reasons for any refusal to comply with a request pursuant to paragraph 4.

6. Requested supervisory authorities shall, as a rule, supply the information requested by other supervisory authorities by electronic means, using a standardised format.

7. Requested supervisory authorities shall not charge a fee for any action taken by them pursuant to a request for mutual assistance. Supervisory authorities may agree on rules to indemnify each other for specific expenditure arising from the provision of mutual assistance in exceptional circumstances.

8. Where a supervisory authority does not provide the information referred to in paragraph 5 of this Article within one month of receiving the request of another supervisory authority, the requesting supervisory authority may adopt a provisional measure on the territory of its Member State in accordance with Article 55(1). In that case, the urgent need to act under Article 66(1) shall be presumed to be met and require an urgent binding decision from the Board pursuant to Article 66(2).

9. The Commission may, by means of implementing acts, specify the format and procedures for mutual assistance referred to in this Article and the arrangements for the exchange of information by electronic means between supervisory authorities, and between supervisory authorities and the Board, in particular the standardised format referred to in paragraph 6 of this Article. Those implementing acts shall be adopted in accordance with the examination procedure referred to in Article 93(2).

Article 62

Joint operations of supervisory authorities

1. The supervisory authorities shall, where appropriate, conduct joint operations including joint investigations and joint enforcement measures in which members or staff of the supervisory authorities of other Member States are involved.

2. Where the controller or processor has establishments in several Member States or where a significant number of data subjects in more than one Member State are likely to be substantially affected by processing operations, a supervisory authority of each of those Member States shall have the right to participate in joint operations. The supervisory authority which is competent pursuant to Article 56(1) or (4) shall invite the supervisory authority of each of those Member States to take part in the joint operations and shall respond without delay to the request of a supervisory authority to participate.

3. A supervisory authority may, in accordance with Member State law, and with the seconding supervisory authority's authorisation, confer powers, including investigative powers on the seconding supervisory authority's members or staff involved in joint operations or, in so far as the law of the Member State of the host supervisory authority permits, allow the seconding supervisory authority's members or staff to exercise their investigative powers in accordance with the law of the Member State of the seconding supervisory authority. Such investigative powers may be exercised only under the guidance and in the presence of members or staff of the host supervisory authority. The seconding supervisory authority's members or staff shall be subject to the Member State law of the host supervisory authority.

4. Where, in accordance with paragraph 1, staff of a seconding supervisory authority operate in another Member State, the Member State of the host supervisory authority shall assume responsibility for their actions, including liability, for any damage caused by them during their operations, in accordance with the law of the Member State in whose territory they are operating.

5. The Member State in whose territory the damage was caused shall make good such damage under the conditions applicable to damage caused by its own staff. The Member State of the seconding supervisory authority whose staff has caused damage to any person in the territory of another Member State shall reimburse that other Member State in full any sums it has paid to the persons entitled on their behalf.

6. Without prejudice to the exercise of its rights *vis-à-vis* third parties and with the exception of paragraph 5, each Member State shall refrain, in the case provided for in paragraph 1, from requesting reimbursement from another Member State in relation to damage referred to in paragraph 4.

7. Where a joint operation is intended and a supervisory authority does not, within one month, comply with the obligation laid down in the second sentence of paragraph 2 of this Article, the other supervisory authorities may adopt a provisional measure on the territory of its Member State in accordance with Article 55. In that case, the urgent need to act under Article 66(1) shall be presumed to be met and require an opinion or an urgent binding decision from the Board pursuant to Article 66(2).

Section 2

Consistency

Article 63

Consistency mechanism

In order to contribute to the consistent application of this Regulation throughout the Union, the supervisory authorities shall cooperate with each other and, where relevant, with the Commission, through the consistency mechanism as set out in this Section.

Article 64

Opinion of the Board

1. The Board shall issue an opinion where a competent supervisory authority intends to adopt any of the measures below. To that end, the competent supervisory authority shall communicate the draft decision to the Board, when it:

(a) aims to adopt a list of the processing operations subject to the requirement for a data protection impact assessment pursuant to Article 35(4);

(b) concerns a matter pursuant to Article 40(7) whether a draft code of conduct or an amendment or extension to a code of conduct complies with this Regulation;

(c) aims to approve the criteria for accreditation of a body pursuant to Article 41(3) or a certification body pursuant to Article 43(3);

(d) aims to determine standard data protection clauses referred to in point (d) of Article 46(2) and in Article 28(8);

(e) aims to authorise contractual clauses referred to in point (a) of Article 46(3); or

(f) aims to approve binding corporate rules within the meaning of Article 47.

2. Any supervisory authority, the Chair of the Board or the Commission may request that any matter of general application or producing effects in more than one Member State be examined by the Board with a view to obtaining an opinion, in particular where a competent supervisory authority does not comply with the obligations for mutual assistance in accordance with Article 61 or for joint operations in accordance with Article 62.

3. In the cases referred to in paragraphs 1 and 2, the Board shall issue an opinion on the matter submitted to it provided that it has not already issued an opinion on the same matter. That opinion shall be adopted within eight weeks by simple majority of the members of the Board. That period may be extended by a further six weeks, taking into account the complexity of the subject matter. Regarding the draft decision referred to in paragraph 1 circulated to the members of the Board in accordance with paragraph 5, a member which has not objected within a reasonable period indicated by the Chair, shall be deemed to be in agreement with the draft decision.

4. Supervisory authorities and the Commission shall, without undue delay, communicate by electronic means to the Board, using a standardised format any relevant information, including as the case may be a summary of the facts, the draft decision, the grounds which make the enactment of such measure necessary, and the views of other supervisory authorities concerned.

5. The Chair of the Board shall, without undue, delay inform by electronic means:

(a) the members of the Board and the Commission of any relevant information which has been communicated to it using a standardised format. The secretariat of the Board shall, where necessary, provide translations of relevant information; and

(b) the supervisory authority referred to, as the case may be, in paragraphs 1 and 2, and the Commission of the opinion and make it public.

6. The competent supervisory authority shall not adopt its draft decision referred to in paragraph 1 within the period referred to in paragraph 3.

7. The supervisory authority referred to in paragraph 1 shall take utmost account of the opinion of the Board and shall, within two weeks after receiving the opinion, communicate to the Chair of the Board by electronic means whether it will maintain or amend its draft decision and, if any, the amended draft decision, using a standardised format.

8. Where the supervisory authority concerned informs the Chair of the Board within the period referred to in paragraph 7 of this Article that it does not intend to follow the opinion of the Board, in whole or in part, providing the relevant grounds, Article 65(1) shall apply.

Article 65

Dispute resolution by the Board

1. In order to ensure the correct and consistent application of this Regulation in individual cases, the Board shall adopt a binding decision in the following cases:

(a) where, in a case referred to in Article 60(4), a supervisory authority concerned has raised a relevant and reasoned objection to a draft decision of the lead authority or the lead authority has rejected such an objection as being not relevant or reasoned. The binding decision shall concern all the matters which are the subject of the relevant and reasoned objection, in particular whether there is an infringement of this Regulation;

(b) where there are conflicting views on which of the supervisory authorities concerned is competent for the main establishment;

(c) where a competent supervisory authority does not request the opinion of the Board in the cases referred to in Article 64(1), or does not follow the opinion of the Board issued under Article 64. In that case, any supervisory authority concerned or the Commission may communicate the matter to the Board.

2. The decision referred to in paragraph 1 shall be adopted within one month from the referral of the subject-matter by a two-thirds majority of the members of the Board. That period may be extended by a further month on account of the complexity of the subject-matter. The decision referred to in paragraph 1 shall be reasoned and addressed to the lead supervisory authority and all the supervisory authorities concerned and binding on them.

3. Where the Board has been unable to adopt a decision within the periods referred to in paragraph 2, it shall adopt its decision within two weeks following the expiration of the second month referred to in paragraph 2 by a simple majority of the members of the Board. Where the members of the Board are split, the decision shall by adopted by the vote of its Chair.

4. The supervisory authorities concerned shall not adopt a decision on the subject matter submitted to the Board under paragraph 1 during the periods referred to in paragraphs 2 and 3.

5. The Chair of the Board shall notify, without undue delay, the decision referred to in paragraph 1 to the supervisory authorities concerned. It shall inform the Commission thereof. The decision shall be published on the website of the Board without delay after the supervisory authority has notified the final decision referred to in paragraph 6.

6. The lead supervisory authority or, as the case may be, the supervisory authority with which the complaint has been lodged shall adopt its final decision on the basis of the decision referred to in paragraph 1 of this Article, without undue delay and at the latest by one month after the Board has notified its decision. The lead supervisory authority or, as the case may be, the supervisory authority with which the complaint has been lodged, shall inform the Board of the date when its final decision is notified respectively to the controller or the processor and to the data subject. The final decision of the supervisory authorities concerned shall be adopted under the terms of Article 60(7), (8) and (9). The final decision shall refer to the decision referred to in paragraph 1 of this Article and shall specify that the decision referred to in that paragraph will be published on the website of the Board in accordance with paragraph 5 of this Article. The final decision shall attach the decision referred to in paragraph 1 of this Article.

Article 66

Urgency procedure

1. In exceptional circumstances, where a supervisory authority concerned considers that there is an urgent need to act in order to protect the rights and freedoms of data subjects, it may, by way of derogation from the consistency mechanism referred to in Articles 63, 64 and 65 or the procedure referred to in Article 60, immediately adopt provisional measures intended to produce legal effects on its own territory with a specified period of validity which shall not exceed three months. The supervisory authority shall, without delay, communicate those measures and the reasons for adopting them to the other supervisory authorities concerned, to the Board and to the Commission.

2. Where a supervisory authority has taken a measure pursuant to paragraph 1 and considers that final measures need urgently be adopted, it may request an urgent opinion or an urgent binding decision from the Board, giving reasons for requesting such opinion or decision.

3. Any supervisory authority may request an urgent opinion or an urgent binding decision, as the case may be, from the Board where a competent supervisory authority has not taken an appropriate measure in a situation where there is an urgent need to act, in order to protect the rights and freedoms of data subjects, giving reasons for requesting such opinion or decision, including for the urgent need to act.

4. By derogation from Article 64(3) and Article 65(2), an urgent opinion or an urgent binding decision referred to in paragraphs 2 and 3 of this Article shall be adopted within two weeks by simple majority of the members of the Board.

Article 67

Exchange of information

The Commission may adopt implementing acts of general scope in order to specify the arrangements for the exchange of information by electronic means between supervisory authorities, and between supervisory authorities and the Board, in particular the standardised format referred to in Article 64.

Those implementing acts shall be adopted in accordance with the examination procedure referred to in Article 93(2).

Section 3

European data protection board

Article 68

European Data Protection Board

1. The European Data Protection Board (the 'Board') is hereby established as a body of the Union and shall have legal personality.

2. The Board shall be represented by its Chair.

3. The Board shall be composed of the head of one supervisory authority of each Member State and of the European Data Protection Supervisor, or their respective representatives.

4. Where in a Member State more than one supervisory authority is responsible for monitoring the application of the provisions pursuant to this Regulation, a joint representative shall be appointed in accordance with that Member State's law.

5. The Commission shall have the right to participate in the activities and meetings of the Board without voting right. The Commission shall designate a representative. The Chair of the Board shall communicate to the Commission the activities of the Board.

6. In the cases referred to in Article 65, the European Data Protection Supervisor shall have voting rights only on decisions which concern principles and rules applicable to the Union institutions, bodies, offices and agencies which correspond in substance to those of this Regulation.

Article 69

Independence

1. The Board shall act independently when performing its tasks or exercising its powers pursuant to Articles 70 and 71.

2. Without prejudice to requests by the Commission referred to in point (b) of Article 70(1) and in Article 70(2), the Board shall, in the performance of its tasks or the exercise of its powers, neither seek nor take instructions from anybody.

Article 70

Tasks of the Board

1. The Board shall ensure the consistent application of this Regulation. To that end, the Board shall, on its own initiative or, where relevant, at the request of the Commission, in particular:

(a) monitor and ensure the correct application of this Regulation in the cases provided for in Articles 64 and 65 without prejudice to the tasks of national supervisory authorities;

(b) advise the Commission on any issue related to the protection of personal data in the Union, including on any proposed amendment of this Regulation;

(c) advise the Commission on the format and procedures for the exchange of information between controllers, processors and supervisory authorities for binding corporate rules;

(d) issue guidelines, recommendations, and best practices on procedures for erasing links, copies or replications of personal data from publicly available communication services as referred to in Article 17(2);

(e) examine, on its own initiative, on request of one of its members or on request of the Commission, any question covering the application of this Regulation and issue guidelines, recommendations and best practices in order to encourage consistent application of this Regulation;

(f) issue guidelines, recommendations and best practices in accordance with point (e) of this paragraph for further specifying the criteria and conditions for decisions based on profiling pursuant to Article 22(2);

(g) issue guidelines, recommendations and best practices in accordance with point (e) of this paragraph for establishing the personal data breaches and determining the undue delay referred to in Article 33(1) and (2) and for the particular circumstances in which a controller or a processor is required to notify the personal data breach;

(h) issue guidelines, recommendations and best practices in accordance with point (e) of this paragraph as to the circumstances in which a personal data breach is likely to result in a high risk to the rights and freedoms of the natural persons referred to in Article 34(1).

(i) issue guidelines, recommendations and best practices in accordance with point (e) of this paragraph for the purpose of further specifying the criteria and requirements for personal data transfers based on binding corporate rules adhered to by controllers and binding corporate rules adhered to by processors and on further necessary requirements to ensure the protection of personal data of the data subjects concerned referred to in Article 47;

(j) issue guidelines, recommendations and best practices in accordance with point (e) of this paragraph for the purpose of further specifying the criteria and requirements for the personal data transfers on the basis of Article 49(1);

(k) draw up guidelines for supervisory authorities concerning the application of measures referred to in Article 58(1), (2) and (3) and the setting of administrative fines pursuant to Article 83;

(l) review the practical application of the guidelines, recommendations and best practices referred to in points (e) and (f);

(m) issue guidelines, recommendations and best practices in accordance with point (e) of this paragraph for establishing common procedures for reporting by natural persons of infringements of this Regulation pursuant to Article 54(2);

(n) encourage the drawing-up of codes of conduct and the establishment of data protection certification mechanisms and data protection seals and marks pursuant to Articles 40 and 42;

(o) carry out the accreditation of certification bodies and its periodic review pursuant to Article 43 and maintain a public register of accredited bodies pursuant to Article 43(6) and of the accredited controllers or processors established in third countries pursuant to Article 42(7);

(p) specify the requirements referred to in Article 43(3) with a view to the accreditation of certification bodies under Article 42;

(q) provide the Commission with an opinion on the certification requirements referred to in Article 43(8);

(r) provide the Commission with an opinion on the icons referred to in Article 12(7);

(s) provide the Commission with an opinion for the assessment of the adequacy of the level of protection in a third country or international organisation, including for the assessment whether a third country, a territory or one or more specified sectors within that third country, or an international organisation no longer ensures an adequate level of protection. To that end, the Commission shall provide the Board with all necessary documentation, including correspondence with the government of the third country, with regard to that third country, territory or specified sector, or with the international organisation.

(t) issue opinions on draft decisions of supervisory authorities pursuant to the consistency mechanism referred to in Article 64(1), on matters submitted pursuant to Article 64(2) and to issue binding decisions pursuant to Article 65, including in cases referred to in Article 66;

(u) promote the cooperation and the effective bilateral and multilateral exchange of information and best practices between the supervisory authorities;

(v) promote common training programmes and facilitate personnel exchanges between the supervisory authorities and, where appropriate, with the supervisory authorities of third countries or with international organisations;

(w) promote the exchange of knowledge and documentation on data protection legislation and practice with data protection supervisory authorities worldwide.

(x) issue opinions on codes of conduct drawn up at Union level pursuant to Article 40(9); and

(y) maintain a publicly accessible electronic register of decisions taken by supervisory authorities and courts on issues handled in the consistency mechanism.

2. Where the Commission requests advice from the Board, it may indicate a time limit, taking into account the urgency of the matter.

3. The Board shall forward its opinions, guidelines, recommendations, and best practices to the Commission and to the committee referred to in Article 93 and make them public.

4. The Board shall, where appropriate, consult interested parties and give them the opportunity to comment within a reasonable period. The Board shall, without prejudice to Article 76, make the results of the consultation procedure publicly available.

Article 71

Reports

1. The Board shall draw up an annual report regarding the protection of natural persons with regard to processing in the Union and, where relevant, in third countries and international organisations. The report shall be made public and be transmitted to the European Parliament, to the Council and to the Commission.

2. The annual report shall include a review of the practical application of the guidelines, recommendations and best practices referred to in point (l) of Article 70(1) as well as of the binding decisions referred to in Article 65.

Article 72

Procedure

1. The Board shall take decisions by a simple majority of its members, unless otherwise provided for in this Regulation.

2. The Board shall adopt its own rules of procedure by a two-thirds majority of its members and organise its own operational arrangements.

Article 73

Chair

1. The Board shall elect a chair and two deputy chairs from amongst its members by simple majority.

2. The term of office of the Chair and of the deputy chairs shall be five years and be renewable once.

Article 74

Tasks of the Chair

1. The Chair shall have the following tasks:

(a) to convene the meetings of the Board and prepare its agenda;

(b) to notify decisions adopted by the Board pursuant to Article 65 to the lead supervisory authority and the supervisory authorities concerned;

(c) to ensure the timely performance of the tasks of the Board, in particular in relation to the consistency mechanism referred to in Article 63.

2. The Board shall lay down the allocation of tasks between the Chair and the deputy chairs in its rules of procedure.

Article 75

Secretariat

1. The Board shall have a secretariat, which shall be provided by the European Data Protection Supervisor.

2. The secretariat shall perform its tasks exclusively under the instructions of the Chair of the Board.

3. The staff of the European Data Protection Supervisor involved in carrying out the tasks conferred on the Board by this Regulation shall be subject to separate reporting lines from the staff involved in carrying out tasks conferred on the European Data Protection Supervisor.

4. Where appropriate, the Board and the European Data Protection Supervisor shall establish and publish a Memorandum of Understanding implementing this Article, determining the terms of their cooperation, and applicable to the staff of the European Data Protection Supervisor involved in carrying out the tasks conferred on the Board by this Regulation.

5. The secretariat shall provide analytical, administrative and logistical support to the Board.

6. The secretariat shall be responsible in particular for:

(a) the day-to-day business of the Board;

(b) communication between the members of the Board, its Chair and the Commission;

(c) communication with other institutions and the public;

(d) the use of electronic means for the internal and external communication;

(e) the translation of relevant information;

(f) the preparation and follow-up of the meetings of the Board;

(g) the preparation, drafting and publication of opinions, decisions on the settlement of disputes between supervisory authorities and other texts adopted by the Board.

Article 76

Confidentiality

1. The discussions of the Board shall be confidential where the Board deems it necessary, as provided for in its rules of procedure.

2. Access to documents submitted to members of the Board, experts and representatives of third parties shall be governed by Regulation (EC) No 1049/2001 of the European Parliament and of the Council [1].

CHAPTER VIII

Remedies, liability and penalties

Article 77

Right to lodge a complaint with a supervisory authority

1. Without prejudice to any other administrative or judicial remedy, every data subject shall have the right to lodge a complaint with a supervisory authority, in particular in the Member State of his or her habitual residence, place of work or place of the alleged infringement if the data subject considers that the processing of personal data relating to him or her infringes this Regulation.

2. The supervisory authority with which the complaint has been lodged shall inform the complainant on the progress and the outcome of the complaint including the possibility of a judicial remedy pursuant to Article 78.

Article 78

Right to an effective judicial remedy against a supervisory authority

1. Without prejudice to any other administrative or non-judicial remedy, each natural or legal person shall have the right to an effective judicial remedy against a legally binding decision of a supervisory authority concerning them.

2. Without prejudice to any other administrative or non-judicial remedy, each data subject shall have the right to a an effective judicial remedy where the supervisory authority which is competent pursuant to Articles 55 and 56 does not handle a complaint or does not inform the data subject within three months on the progress or outcome of the complaint lodged pursuant to Article 77.

3. Proceedings against a supervisory authority shall be brought before the courts of the Member State where the supervisory authority is established.

4. Where proceedings are brought against a decision of a supervisory authority which was preceded by an opinion or a decision of the Board in the consistency mechanism, the supervisory authority shall forward that opinion or decision to the court.

Article 79

Right to an effective judicial remedy against a controller or processor

1. Without prejudice to any available administrative or non-judicial remedy, including the right to lodge a complaint with a supervisory authority pursuant to Article 77, each data subject shall have the right to an effective judicial remedy where he or she considers that his or her rights under this Regulation have been infringed as a result of the processing of his or her personal data in non-compliance with this Regulation.

2. Proceedings against a controller or a processor shall be brought before the courts of the Member State where the controller or processor has an establishment. Alternatively, such proceedings may be brought before the courts of the Member State where the data subject has his or her habitual residence, unless the controller or processor is a public authority of a Member State acting in the exercise of its public powers.

---

(1) Regulation (EC) No 1049/2001 of the European Parliament and of the Council of 30 May 2001 regarding public access to European Parliament, Council and Commission documents (OJ L 145, 31.5.2001, p. 43).

Article 80

Representation of data subjects

1. The data subject shall have the right to mandate a not-for-profit body, organisation or association which has been properly constituted in accordance with the law of a Member State, has statutory objectives which are in the public interest, and is active in the field of the protection of data subjects' rights and freedoms with regard to the protection of their personal data to lodge the complaint on his or her behalf, to exercise the rights referred to in Articles 77, 78 and 79 on his or her behalf, and to exercise the right to receive compensation referred to in Article 82 on his or her behalf where provided for by Member State law.

2. Member States may provide that any body, organisation or association referred to in paragraph 1 of this Article, independently of a data subject's mandate, has the right to lodge, in that Member State, a complaint with the supervisory authority which is competent pursuant to Article 77 and to exercise the rights referred to in Articles 78 and 79 if it considers that the rights of a data subject under this Regulation have been infringed as a result of the processing.

Article 81

Suspension of proceedings

1. Where a competent court of a Member State has information on proceedings, concerning the same subject matter as regards processing by the same controller or processor, that are pending in a court in another Member State, it shall contact that court in the other Member State to confirm the existence of such proceedings.

2. Where proceedings concerning the same subject matter as regards processing of the same controller or processor are pending in a court in another Member State, any competent court other than the court first seized may suspend its proceedings.

3. Where those proceedings are pending at first instance, any court other than the court first seized may also, on the application of one of the parties, decline jurisdiction if the court first seized has jurisdiction over the actions in question and its law permits the consolidation thereof.

Article 82

Right to compensation and liability

1. Any person who has suffered material or non-material damage as a result of an infringement of this Regulation shall have the right to receive compensation from the controller or processor for the damage suffered.

2. Any controller involved in processing shall be liable for the damage caused by processing which infringes this Regulation. A processor shall be liable for the damage caused by processing only where it has not complied with obligations of this Regulation specifically directed to processors or where it has acted outside or contrary to lawful instructions of the controller.

3. A controller or processor shall be exempt from liability under paragraph 2 if it proves that it is not in any way responsible for the event giving rise to the damage.

4. Where more than one controller or processor, or both a controller and a processor, are involved in the same processing and where they are, under paragraphs 2 and 3, responsible for any damage caused by processing, each controller or processor shall be held liable for the entire damage in order to ensure effective compensation of the data subject.

5. Where a controller or processor has, in accordance with paragraph 4, paid full compensation for the damage suffered, that controller or processor shall be entitled to claim back from the other controllers or processors involved in the same processing that part of the compensation corresponding to their part of responsibility for the damage, in accordance with the conditions set out in paragraph 2.

6. Court proceedings for exercising the right to receive compensation shall be brought before the courts competent under the law of the Member State referred to in Article 79(2).

Article 83

General conditions for imposing administrative fines

1. Each supervisory authority shall ensure that the imposition of administrative fines pursuant to this Article in respect of infringements of this Regulation referred to in paragraphs 4, 5 and 6 shall in each individual case be effective, proportionate and dissuasive.

2. Administrative fines shall, depending on the circumstances of each individual case, be imposed in addition to, or instead of, measures referred to in points (a) to (h) and (j) of Article 58(2). When deciding whether to impose an administrative fine and deciding on the amount of the administrative fine in each individual case due regard shall be given to the following:

(a) the nature, gravity and duration of the infringement taking into account the nature scope or purpose of the processing concerned as well as the number of data subjects affected and the level of damage suffered by them;

(b) the intentional or negligent character of the infringement;

(c) any action taken by the controller or processor to mitigate the damage suffered by data subjects;

(d) the degree of responsibility of the controller or processor taking into account technical and organisational measures implemented by them pursuant to Articles 25 and 32;

(e) any relevant previous infringements by the controller or processor;

(f) the degree of cooperation with the supervisory authority, in order to remedy the infringement and mitigate the possible adverse effects of the infringement;

(g) the categories of personal data affected by the infringement;

(h) the manner in which the infringement became known to the supervisory authority, in particular whether, and if so to what extent, the controller or processor notified the infringement;

(i) where measures referred to in Article 58(2) have previously been ordered against the controller or processor concerned with regard to the same subject-matter, compliance with those measures;

(j) adherence to approved codes of conduct pursuant to Article 40 or approved certification mechanisms pursuant to Article 42; and

(k) any other aggravating or mitigating factor applicable to the circumstances of the case, such as financial benefits gained, or losses avoided, directly or indirectly, from the infringement.

3. If a controller or processor intentionally or negligently, for the same or linked processing operations, infringes several provisions of this Regulation, the total amount of the administrative fine shall not exceed the amount specified for the gravest infringement.

4. Infringements of the following provisions shall, in accordance with paragraph 2, be subject to administrative fines up to 10 000 000 EUR, or in the case of an undertaking, up to 2 % of the total worldwide annual turnover of the preceding financial year, whichever is higher:

(a) the obligations of the controller and the processor pursuant to Articles 8, 11, 25 to 39 and 42 and 43;

(b) the obligations of the certification body pursuant to Articles 42 and 43;

(c) the obligations of the monitoring body pursuant to Article 41(4).

5. Infringements of the following provisions shall, in accordance with paragraph 2, be subject to administrative fines up to 20 000 000 EUR, or in the case of an undertaking, up to 4 % of the total worldwide annual turnover of the preceding financial year, whichever is higher:

(a) the basic principles for processing, including conditions for consent, pursuant to Articles 5, 6, 7 and 9;

(b) the data subjects' rights pursuant to Articles 12 to 22;

(c) the transfers of personal data to a recipient in a third country or an international organisation pursuant to Articles 44 to 49;

(d) any obligations pursuant to Member State law adopted under Chapter IX;

(e) non-compliance with an order or a temporary or definitive limitation on processing or the suspension of data flows by the supervisory authority pursuant to Article 58(2) or failure to provide access in violation of Article 58(1).

6. Non-compliance with an order by the supervisory authority as referred to in Article 58(2) shall, in accordance with paragraph 2 of this Article, be subject to administrative fines up to 20 000 000 EUR, or in the case of an undertaking, up to 4 % of the total worldwide annual turnover of the preceding financial year, whichever is higher.

7. Without prejudice to the corrective powers of supervisory authorities pursuant to Article 58(2), each Member State may lay down the rules on whether and to what extent administrative fines may be imposed on public authorities and bodies established in that Member State.

8. The exercise by the supervisory authority of its powers under this Article shall be subject to appropriate procedural safeguards in accordance with Union and Member State law, including effective judicial remedy and due process.

9. Where the legal system of the Member State does not provide for administrative fines, this Article may be applied in such a manner that the fine is initiated by the competent supervisory authority and imposed by competent national courts, while ensuring that those legal remedies are effective and have an equivalent effect to the administrative fines imposed by supervisory authorities. In any event, the fines imposed shall be effective, proportionate and dissuasive. Those Member States shall notify to the Commission the provisions of their laws which they adopt pursuant to this paragraph by 25 May 2018 and, without delay, any subsequent amendment law or amendment affecting them.

Article 84

Penalties

1. Member States shall lay down the rules on other penalties applicable to infringements of this Regulation in particular for infringements which are not subject to administrative fines pursuant to Article 83, and shall take all measures necessary to ensure that they are implemented. Such penalties shall be effective, proportionate and dissuasive.

2. Each Member State shall notify to the Commission the provisions of its law which it adopts pursuant to paragraph 1, by 25 May 2018 and, without delay, any subsequent amendment affecting them.

CHAPTER IX

Provisions relating to specific processing situations

Article 85

Processing and freedom of expression and information

1. Member States shall by law reconcile the right to the protection of personal data pursuant to this Regulation with the right to freedom of expression and information, including processing for journalistic purposes and the purposes of academic, artistic or literary expression.

2. For processing carried out for journalistic purposes or the purpose of academic artistic or literary expression, Member States shall provide for exemptions or derogations from Chapter II (principles), Chapter III (rights of the data subject), Chapter IV (controller and processor), Chapter V (transfer of personal data to third countries or international organisations), Chapter VI (independent supervisory authorities), Chapter VII (cooperation and consistency) and Chapter IX (specific data processing situations) if they are necessary to reconcile the right to the protection of personal data with the freedom of expression and information.

3. Each Member State shall notify to the Commission the provisions of its law which it has adopted pursuant to paragraph 2 and, without delay, any subsequent amendment law or amendment affecting them.

Article 86

Processing and public access to official documents

Personal data in official documents held by a public authority or a public body or a private body for the performance of a task carried out in the public interest may be disclosed by the authority or body in accordance with Union or Member State law to which the public authority or body is subject in order to reconcile public access to official documents with the right to the protection of personal data pursuant to this Regulation.

Article 87

Processing of the national identification number

Member States may further determine the specific conditions for the processing of a national identification number or any other identifier of general application. In that case the national identification number or any other identifier of general application shall be used only under appropriate safeguards for the rights and freedoms of the data subject pursuant to this Regulation.

Article 88

Processing in the context of employment

1. Member States may, by law or by collective agreements, provide for more specific rules to ensure the protection of the rights and freedoms in respect of the processing of employees' personal data in the employment context, in particular for the purposes of the recruitment, the performance of the contract of employment, including discharge of obligations laid down by law or by collective agreements, management, planning and organisation of work, equality and diversity in the workplace, health and safety at work, protection of employer's or customer's property and for the purposes of the exercise and enjoyment, on an individual or collective basis, of rights and benefits related to employment, and for the purpose of the termination of the employment relationship.

2. Those rules shall include suitable and specific measures to safeguard the data subject's human dignity, legitimate interests and fundamental rights, with particular regard to the transparency of processing, the transfer of personal data within a group of undertakings, or a group of enterprises engaged in a joint economic activity and monitoring systems at the work place.

3. Each Member State shall notify to the Commission those provisions of its law which it adopts pursuant to paragraph 1, by 25 May 2018 and, without delay, any subsequent amendment affecting them.

Article 89

Safeguards and derogations relating to processing for archiving purposes in the public interest, scientific or historical research purposes or statistical purposes

1. Processing for archiving purposes in the public interest, scientific or historical research purposes or statistical purposes, shall be subject to appropriate safeguards, in accordance with this Regulation, for the rights and freedoms of the data subject. Those safeguards shall ensure that technical and organisational measures are in place in particular in

order to ensure respect for the principle of data minimisation. Those measures may include pseudonymisation provided that those purposes can be fulfilled in that manner. Where those purposes can be fulfilled by further processing which does not permit or no longer permits the identification of data subjects, those purposes shall be fulfilled in that manner.

2. Where personal data are processed for scientific or historical research purposes or statistical purposes, Union or Member State law may provide for derogations from the rights referred to in Articles 15, 16, 18 and 21 subject to the conditions and safeguards referred to in paragraph 1 of this Article in so far as such rights are likely to render impossible or seriously impair the achievement of the specific purposes, and such derogations are necessary for the fulfilment of those purposes.

3. Where personal data are processed for archiving purposes in the public interest, Union or Member State law may provide for derogations from the rights referred to in Articles 15, 16, 18, 19, 20 and 21 subject to the conditions and safeguards referred to in paragraph 1 of this Article in so far as such rights are likely to render impossible or seriously impair the achievement of the specific purposes, and such derogations are necessary for the fulfilment of those purposes.

4. Where processing referred to in paragraphs 2 and 3 serves at the same time another purpose, the derogations shall apply only to processing for the purposes referred to in those paragraphs.

Article 90

Obligations of secrecy

1. Member States may adopt specific rules to set out the powers of the supervisory authorities laid down in points (e) and (f) of Article 58(1) in relation to controllers or processors that are subject, under Union or Member State law or rules established by national competent bodies, to an obligation of professional secrecy or other equivalent obligations of secrecy where this is necessary and proportionate to reconcile the right of the protection of personal data with the obligation of secrecy. Those rules shall apply only with regard to personal data which the controller or processor has received as a result of or has obtained in an activity covered by that obligation of secrecy.

2. Each Member State shall notify to the Commission the rules adopted pursuant to paragraph 1, by 25 May 2018 and, without delay, any subsequent amendment affecting them.

Article 91

Existing data protection rules of churches and religious associations

1. Where in a Member State, churches and religious associations or communities apply, at the time of entry into force of this Regulation, comprehensive rules relating to the protection of natural persons with regard to processing, such rules may continue to apply, provided that they are brought into line with this Regulation.

2. Churches and religious associations which apply comprehensive rules in accordance with paragraph 1 of this Article shall be subject to the supervision of an independent supervisory authority, which may be specific, provided that it fulfils the conditions laid down in Chapter VI of this Regulation.

CHAPTER X

Delegated acts and implementing acts

Article 92

Exercise of the delegation

1. The power to adopt delegated acts is conferred on the Commission subject to the conditions laid down in this Article.

2. The delegation of power referred to in Article 12(8) and Article 43(8) shall be conferred on the Commission for an indeterminate period of time from 24 May 2016.

3. The delegation of power referred to in Article 12(8) and Article 43(8) may be revoked at any time by the European Parliament or by the Council. A decision of revocation shall put an end to the delegation of power specified in that decision. It shall take effect the day following that of its publication in the *Official Journal of the European Union* or at a later date specified therein. It shall not affect the validity of any delegated acts already in force.

4. As soon as it adopts a delegated act, the Commission shall notify it simultaneously to the European Parliament and to the Council.

5. A delegated act adopted pursuant to Article 12(8) and Article 43(8) shall enter into force only if no objection has been expressed by either the European Parliament or the Council within a period of three months of notification of that act to the European Parliament and the Council or if, before the expiry of that period, the European Parliament and the Council have both informed the Commission that they will not object. That period shall be extended by three months at the initiative of the European Parliament or of the Council.

Article 93

Committee procedure

1. The Commission shall be assisted by a committee. That committee shall be a committee within the meaning of Regulation (EU) No 182/2011.

2. Where reference is made to this paragraph, Article 5 of Regulation (EU) No 182/2011 shall apply.

3. Where reference is made to this paragraph, Article 8 of Regulation (EU) No 182/2011, in conjunction with Article 5 thereof, shall apply.

CHAPTER XI

Final provisions

Article 94

Repeal of Directive 95/46/EC

1. Directive 95/46/EC is repealed with effect from 25 May 2018.

2. References to the repealed Directive shall be construed as references to this Regulation. References to the Working Party on the Protection of Individuals with regard to the Processing of Personal Data established by Article 29 of Directive 95/46/EC shall be construed as references to the European Data Protection Board established by this Regulation.

Article 95

Relationship with Directive 2002/58/EC

This Regulation shall not impose additional obligations on natural or legal persons in relation to processing in connection with the provision of publicly available electronic communications services in public communication networks in the Union in relation to matters for which they are subject to specific obligations with the same objective set out in Directive 2002/58/EC.

Article 96

Relationship with previously concluded Agreements

International agreements involving the transfer of personal data to third countries or international organisations which were concluded by Member States prior to 24 May 2016, and which comply with Union law as applicable prior to that date, shall remain in force until amended, replaced or revoked.

Article 97

Commission reports

1. By 25 May 2020 and every four years thereafter, the Commission shall submit a report on the evaluation and review of this Regulation to the European Parliament and to the Council. The reports shall be made public.

2. In the context of the evaluations and reviews referred to in paragraph 1, the Commission shall examine, in particular, the application and functioning of:

(a) Chapter V on the transfer of personal data to third countries or international organisations with particular regard to decisions adopted pursuant to Article 45(3) of this Regulation and decisions adopted on the basis of Article 25(6) of Directive 95/46/EC;

(b) Chapter VII on cooperation and consistency.

3. For the purpose of paragraph 1, the Commission may request information from Member States and supervisory authorities.

4. In carrying out the evaluations and reviews referred to in paragraphs 1 and 2, the Commission shall take into account the positions and findings of the European Parliament, of the Council, and of other relevant bodies or sources.

5. The Commission shall, if necessary, submit appropriate proposals to amend this Regulation, in particular taking into account of developments in information technology and in the light of the state of progress in the information society.

Article 98

Review of other Union legal acts on data protection

The Commission shall, if appropriate, submit legislative proposals with a view to amending other Union legal acts on the protection of personal data, in order to ensure uniform and consistent protection of natural persons with regard to processing. This shall in particular concern the rules relating to the protection of natural persons with regard to processing by Union institutions, bodies, offices and agencies and on the free movement of such data.

Article 99

Entry into force and application

1. This Regulation shall enter into force on the twentieth day following that of its publication in the *Official Journal of the European Union*.

2. It shall apply from 25 May 2018.

This Regulation shall be binding in its entirety and directly applicable in all Member States.

Done at Brussels, 27 April 2016.

For the European Parliament	For the Council
The President	*The President*
M. SCHULZ	J.A. HENNIS-PLASSCHAERT
