# Supplementary material for: Tick-Borne Encephalitis Virus Vaccination among Tourists in a High-Prevalence Area (Italy, 2023): A Cross-Sectional Study
Source: Trop Med Infect Dis. 2023 Nov 2;8(11):491. doi: 10.3390/tropicalmed8110491 (PMC10674593; doi:10.3390/tropicalmed8110491)
Supplement: Supplementary file 1 [file tropicalmed-08-00491-s001.zip › Tables S1-S3.pdf]

Table S1. STROBE statement, giving a checklist of items that should be included in reports of cross-sectional studies.

	Item No.	Recommendation	Page
**Title and abstract**	1	(a) Indicate the study's design with a commonly used term in the title or the abstract	1
		(b) Provide in the abstract an informative and balanced summary of what was done and what was found	1
		**Introduction**	
Background/rationale	2	Explain the scientific background and rationale for the investigation being reported	1–3
Objectives	3	State the specific objectives, including any prespecified hypotheses	2–3
		**Methods**	
Study design	4	Present key elements of study design early in the paper	3
Setting	5	Describe the setting, locations, and relevant dates, including periods of recruitment, exposure, follow-up, and data collection	3
Participants	6	(a) Give the eligibility criteria and the sources and methods of selection of participants	3–4
Variables	7	Clearly define all outcomes, exposures, predictors, potential confounders, and effect modifiers. Give diagnostic criteria, if applicable	3–4
Data sources/measurement	8	For each variable of interest, give sources of data and details of methods of assessment (measurement). Describe comparability of assessment methods if there is more than one group	4–5
Bias	9	Describe any efforts to address potential sources of bias	4–5
Study size	10	Explain how the study size was arrived at	3
Quantitative variables	11	Explain how quantitative variables were handled in the analyses. If applicable, describe which groupings were chosen and why	5
Statistical methods	12	(a) Describe all statistical methods, including those used to control for confounding	5–6
		(b) Describe any methods used to examine subgroups and interactions	5
		(c) Explain how missing data were addressed	5
		(d) If applicable, describe analytical methods, taking account of sampling strategy	5
		(e) Describe any sensitivity analyses	-
		**Results**	
Participants	13	(a) Report numbers of individuals at each stage of study—e.g., numbers potentially eligible, examined for eligibility, confirmed eligible, included in the study, completing follow-up, and analyzed	6
		(b) Give reasons for non-participation at each stage	6
		(c) Consider the use of a flow diagram	6
Descriptive data	14	(a) Give characteristics of study participants (e.g., demographic, clinical, and social) and information on exposures and potential confounders	6–7
		(b) Indicate the number of participants with missing data for each variable of interest	6–7

Outcome data	15	Report numbers of outcome events or summary measures	9
Main results	16	(a) Give unadjusted estimates and, if applicable, confounder-adjusted estimates and their precision (e.g., 95% confidence interval). Make clear which confounders were adjusted for and why they were included	10–13
		(b) Report category boundaries when continuous variables were categorized	10–13
		(c) If relevant, consider translating estimates of relative risk into absolute risk for a meaningful time period	-
Other analyses	17	Report other analyses done—e.g., analyses of subgroups and interactions, and sensitivity analyses	Appendix A Tables A5–A8, Appendix A Figures A3 and A4
**Discussion**			
Key results	18	Summarize key results with reference to study objectives	14
Limitations	19	Discuss the limitations of the study, taking into account sources of potential bias or imprecision. Discuss both the direction and magnitude of any potential bias	16–17
Interpretation	20	Give a cautious overall interpretation of results, considering objectives, limitations, multiplicity of analyses, results from similar studies, and other relevant evidence	14–16
Generalizability	21	Discuss the generalizability (external validity) of the study results	15–16
**Other information**			
Funding	22	Give the source of funding and the role of the funders for the present study and, if applicable, for the original study on which the present article is based	18

Table S2. Authors' translation of the informed consent.

Informed Consent. Estimated participant: the present survey has been developed and shared with the aim of assessing the knowledge, attitudes, and practices of the people traveling to the Dolomites area since 2017 regarding their acceptance of the vaccine against tick-borne encephalitis. Alongside vaccination practices, preventive practices against tick bites will also be ascertained. The present survey has only scientific aims. No economic or similar compensation is guaranteed for the participants.
While we thank you for your cooperation, we stress that web-based surveys must fulfill the requirements represented by the "Helsinki Protocol" and EU Regulation 2016/679.<br>In order to fulfill the requirements of the Helsinki Protocol, we are requesting to formally share your consent. Without your consent, the survey will not continue. Even after your consent, you can leave the present survey at any moment until the sharing of the questionnaire (button "share module" at the end of the questionnaire. Moreover, we stress that the questionnaire will be registered in an anonymous form and in no way could be associated with the compiler, as we will not retain any specific, individual information (e.g., signature, personal address, etc.). All requested personal data are generic ones, and are functional to the demographic analyses (gender, age, etc.).
According to EU Regulation 2016/279 (GDPR), we also state that:<br>(1) the data controller and processor, as well as the person responsible for their retention during the analyses, will be Dr. **********, whom you can ask about the process through his personal email (*********). Collected data are generic ones, with the SOLE SCIENTIFIC AIMS that have been previously reported. Please be aware that all personal data must be shared with criminal law authorities without previous personal consent in those cases that are specifically reported by the current legal framework; without a specific request, retrieved data will not be shared with third parties.

(2) After the completion of the questionnaire, we cannot identify the compiler in any way; since the questionnaire is totally anonymous by design, we cannot perform any modification or correction to the data collected, or their removal as well.<br>Data will be retained only for the time strictly required for the aforementioned analyses.

Table S3. Authors' translation of the questionnaire.

**Section 1. Your Personal Experience with the Dolomites Area**			
Are you 18 years old?	(YES) (NO) (NO ANSWER)		
Have you any previous knowledge of tick-borne encephalitis?	(YES) (NO) (NO ANSWER)		
Have you traveled to the Dolomites area since 2017?	(YES) (NO) (NO ANSWER)		
**Section 2. To your knowledge (please mark the correct answer)**	**TRUE**	**FALSE**	**DON'T KNOW**
Q01. Ticks feeding on cats and dogs may cause human disease.	( )	( )	( )
Q02. Ticks prefer to live in damp, shady areas.	( )	( )	( )
Q03. The majority of ticks that are found in Italy may spread severe human diseases.	( )	( )	( )
Q04. Tick-borne human diseases are more frequently diagnosed between May and September.	( )	( )	( )
Q05. The available vaccines protect against all tick-borne human diseases.	( )	( )	( )
Q06. Bites from ticks removed within 24 h of their first bite do not require any treatment.	( )	( )	( )
Q07. All tick-borne human diseases manifest themselves within 2 days of the first bite.	( )	( )	( )
Q08. Tick-borne human diseases may always be treated with antibiotics.	( )	( )	( )
Q09. Tick bites are frequently complicated by local infections.	( )	( )	( )
Q10. Local treatment with alcohol on the bite site is appropriate for tick removal.	( )	( )	( )
Q11. After tick removal, the bite site should be checked for the tick head.	( )	( )	( )
Q12. The milk of animals affected by tick-borne disease may harbor the pathogens.	( )	( )	( )
**Section 3. Your Previous Experiences with Tick-borne Encephalitis Virus Vaccine**			
Did someone recommend that you consider the uptake of the TBE vaccine?			
General Practitioner	( )		
Occupational Physician	( )		
Vaccination Service of the Local Health Unit	( )		
Emergency Department Professionals	( )		
Others	( )		
Have you previously received the TBE vaccine?			
Yes	( )		
No, I did not know about the TBE vaccine	( )		
No, I am not at risk	( )		
No, I did know about the TBE vaccine but I had not enough time	( )		
No, I have doubts about vaccines	( )		
No, I have fears about vaccine side effects	( )		
No, it is too expensive	( )		
No, the vaccine was not available	( )		
**In case of previous TBE vaccination, please check the statement most accurately describing your understanding of vaccines and immunizations**			
Vaccines protect those who cannot be vaccinated against infectious diseases	( )		
Vaccines avoid complications from several infectious diseases	( )		
Vaccines avoid the spreading of infectious diseases in the general population	( )		
Vaccines help avoid contracting the diseases they target	( )		

**In general, how would you rate your attitude toward vaccines and vaccination?**	1. Totally against<br>2. Against<br>3. Neutral<br>4. Favorable<br>5. Totally favorable
**Section 3. Your previous experiences with tick bites**	
**Have you noticed a tick bite received during 2022?**	(YES) (NO) (NO ANSWER)
**In case of a tick bite, the tick was removed by:**<br>Any HCW<br>Friends/Relatives<br>Yourself	 ( )<br>( )<br>( )
In case of a tick bite, have you received any antibiotic treatment?	(YES) (NO) (NO ANSWER)
In case of a tick bite, has there been any laboratory follow-up?	(YES) (NO) (NO ANSWER)
In case of a tick bite, have you received a diagnosis of Lyme disease?	(YES) (NO) (NO ANSWER)
In case of a tick bite, have you received a diagnosis of TBEV infection?	(YES) (NO) (NO ANSWER)
In case of a tick bite, have you received a diagnosis of skin infection on the tick bite?	(YES) (NO) (NO ANSWER)
Were any of your friends or any member of your family ever diagnosed with TBE?	(YES) (NO) (NO ANSWER)
**Section 4. Preventive measures you put in place when going outdoors in mountain ranges**	
Use of repellent	( ) never<br>( ) rarely<br>( ) sometimes<br>( ) often<br>( ) always
Wear light-colored clothing	( ) never<br>( ) rarely<br>( ) sometimes<br>( ) often<br>( ) always
Wear long sleeves and pants	( ) never<br>( ) rarely<br>( ) sometimes<br>( ) often<br>( ) always
Tuck pants into socks or boots	( ) never<br>( ) rarely<br>( ) sometimes<br>( ) often<br>( ) always
Perform a body check	( ) never<br>( ) rarely<br>( ) sometimes<br>( ) often<br>( ) always
Wear a hat	( ) never<br>( ) rarely<br>( ) sometimes<br>( ) often<br>( ) always
Avoid typical tick habitats	( ) never<br>( ) rarely

	( ) sometimes<br>( ) often<br>( ) always
**Section 5. Your understanding of tick bites**	
According to your knowledge and personal experience, which one(s) of the following signs and symptoms appearing within 30 days from the event could be associated with a tick bite?	
Fever ≥ 38 °C	1—totally disagree<br>2—disagree<br>3—neutral<br>4—agree<br>5—totally agree
Headache	1—totally disagree<br>2—disagree<br>3—neutral<br>4—agree<br>5—totally agree
Neck stiffness	1—totally disagree<br>2—disagree<br>3—neutral<br>4—agree<br>5—totally agree
Difficulty in breathing	1—totally disagree<br>2—disagree<br>3—neutral<br>4—agree<br>5—totally agree
Fatigue	1—totally disagree<br>2—disagree<br>3—neutral<br>4—agree<br>5—totally agree
Muscle Ache	1—totally disagree<br>2—disagree<br>3—neutral<br>4—agree<br>5—totally agree
Facial paralysis	1—totally disagree<br>2—disagree<br>3—neutral<br>4—agree<br>5—totally agree
Donut-shaped red rash	1—totally disagree<br>2—disagree<br>3—neutral<br>4—agree<br>5—totally agree
Black skin lesions on the neck/back	1—totally disagree<br>2—disagree<br>3—neutral<br>4—agree

	5—totally agree
Enlarged lymph nodes	1—totally disagree<br>2—disagree<br>3—neutral<br>4—agree<br>5—totally agree
According to your knowledge and personal experience, which one(s) of the following conditions could be associated with a tick bite, even months or years after the event?	
Joint pain	1—totally disagree<br>2—disagree<br>3—neutral<br>4—agree<br>5—totally agree
Amnesia	1—totally disagree<br>2—disagree<br>3—neutral<br>4—agree<br>5—totally agree
Drowsiness/Confusion	1—totally disagree<br>2—disagree<br>3—neutral<br>4—agree<br>5—totally agree
Mood changes	1—totally disagree<br>2—disagree<br>3—neutral<br>4—agree<br>5—totally agree
Persisting/relapsing headache	1—totally disagree<br>2—disagree<br>3—neutral<br>4—agree<br>5—totally agree
Cardiac disorders	1—totally disagree<br>2—disagree<br>3—neutral<br>4—agree<br>5—totally agree
Pneumonia	1—totally disagree<br>2—disagree<br>3—neutral<br>4—agree<br>5—totally agree
Depression	1—totally disagree<br>2—disagree<br>3—neutral<br>4—agree<br>5—totally agree
Muscle Weakness/Muscle Pain	1—totally disagree<br>2—disagree<br>3—neutral

	4—agree<br>5—totally agree
**Section 6. Your perception of the risk associated with tick-borne encephalitis**	
According to your current understanding, TBEV infections can be acknowledged:	
in terms of their occurrence, as:	(1) Extremely infrequent<br>(2) Infrequent<br>(3) Neither frequent nor infrequent<br>(4) Frequent<br>Very frequent
in terms of their severity, as:	(1) Not at all severe<br>(2) Of low severity<br>(3) Neither severe nor indolent<br>(4) Severe<br>(5) Very severe
According to your current understanding, the side effects of TBEV vaccination can be acknowledged:	
in terms of their occurrence, as:	(1) Extremely infrequent<br>(2) Infrequent<br>(3) Neither frequent nor infrequent<br>(4) Frequent<br>(6) Very frequent
in terms of their severity, as:	(1) Not at all severe<br>(2) Of low severity<br>(3) Neither severe nor indolent<br>(4) Severe<br>(5) Very severe
**Section 7: Finally, please provide some general information about you:**	
How old are you?	( ) < 20 years<br>( ) 20–29 years<br>( ) 30–39 years<br>( ) 40–49 years<br>( ) 50–59 years<br>( ) 60–69 years<br>( ) 70 years or more
You identify yourself as	[Male] [Female]<br>[No Answer]
Do you live in …	
Northern Italy, Autonomous Province of Trento, the Province of Belluno, or the Province of Gorizia	( )
Other provinces from Northern Italy (Piedmont, Liguria, Lombardy, Emilia-Romagna, Autonomous Province of Bolzano, Veneto (excluding the province of Belluno), Friuli-Venezia-Giulia (excluding the province of Gorizia))	( )
Central Italy (Tuscany, Umbria, Marche, Latium)	( )
Southern Italy (Campania, Abruzzo, Apulia, Molise, Basilicata, Calabria)	( )
Major Islands (Sicily, Sardinia)	( )
Do you have any pets?	[yes] [no] [no answer]

Do you work in a healthcare setting?	[yes] [no] [no answer]
Do you work in an agricultural setting?	[yes] [no] [no answer]
Your highest educational achievement	
Primary/Secondary school	( ) Up to 8 years of formal education
High school	( ) 9 to 14 years of formal education
University	( ) more than 14 years of formal education
None/Not declared	( )
Your main information source on vaccines and vaccinations	
Conventional media (television, radio, newspapers)	( )
Internet, official websites	( )
Internet: social media, personal blogs, forum	( )
Friends, members of the family	( )
Professional courses	( )
General Practitioner/Pediatrician	( )
Occupational Physician	( )
